# Regioselectivity and diastereoselectivity of three-component reaction of α-amino acid, dialkyl acetylenedicarboxylates and 2-arylidene-1,3-indanediones

**DOI:** 10.1038/s41598-017-12361-z

**Published:** 2017-09-29

**Authors:** Liang Chen, Jing Sun, Ying Huang, Yu Zhang, Chao-Guo Yan

**Affiliations:** College of Chemistry & Chemical Engineering, Yangzhou University, Yangzhou, 225002 China

## Abstract

The 1,3-dipolar cycloaddition of active azomethine ylide, which were generated *in situ* from addition reaction of α-amino acids with dialkyl acetylenedicarboxylates, with 2-arylidene-1,3-indanediones showed versatile regioselectivity and diastereoselectivity. The reaction of sarcosine and glycine afforded one kind of functionalized spiro[indene-2,3′-pyrrolidines]. The other primary α-amino acids such as alanine, phenylalanine and leucine gave another kind of regioisomeric spiro[indene-2,3′-pyrrolidines]. The cyclic α-amino acids resulted in the corresponding spiro[indene-2,2′-pyrrolizines] and [indene-2,6’-pyrrolo[1,2-c]thiazoles].

## Introduction

**Figure 1:**
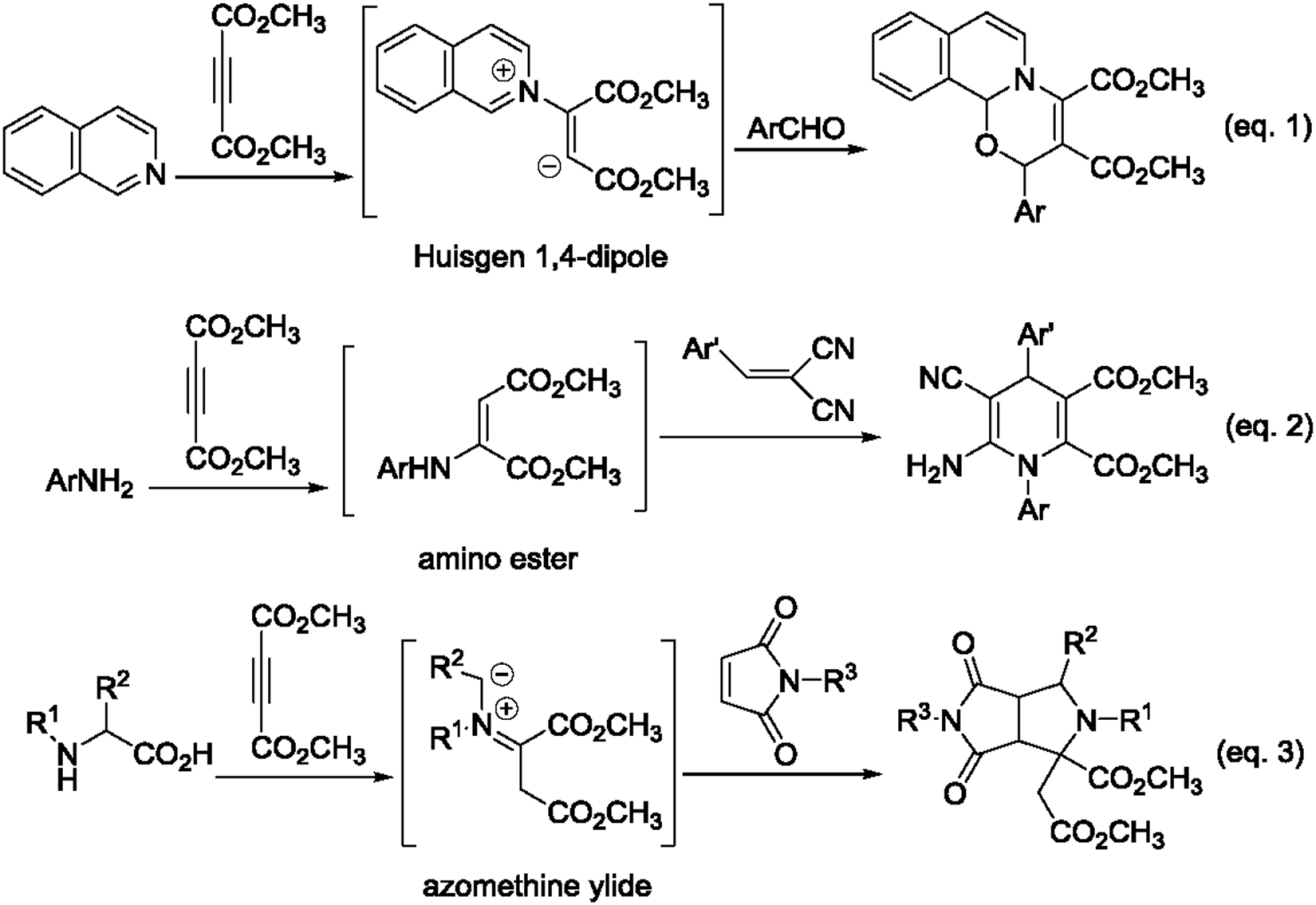
Generation of active intermediates via reaction of electron-deficient alkynes and N-nucleophiles.

The tertiary amine such as pyridine, quinolone, isoquinoline promoted nucleophilic addition reaction of electron-deficient alkynes have attracted much research efforts in the past decades^1–3^. Traditionally, the addition reaction of aromatic nitrogen-containing heterocycles such as pyridine, quinolone and isoquinoline to election-deficient alkynes including dialkyl acetylenedicarboxylates resulted in active Huisgen 1,4-dipoles, which can be trapped by various reagents including dienophiles to accomplish diverse carbon-carbon bond formation reactions and various heterocyclic constructions (Fig. 1, eq. 1)^4–13^. In this decades, the similar active enamines derived from addition reaction of aromatic amines to electron-deficient alkynes were also widely recognized as useful synthon for the synthesis of various *N-* and *N*, *O*-heterocycles (Fig. 1, eq. 2)^14–16^. These kind of active enamines have the similar structural characters to that of the widely employed *β-*enaminone and *β-*enmino esters and showed versatile applications in construction of nitrogen-containing heterocycles^17–36^. Recently, we have found that the reaction of *α*-amino acids with dialkyl acetylenedicarboxylates afforded a new kind of azomethine ylide, which could reacted with some typical dienophiles such as *N*-substituted maleimides and 3-methyleneoxindoles to give diverse pyrrolo[3,4-*c*]pyrrole and spiro[indoline-3,3′-pyrrolidine] derivatives (Fig. 1, eq. 3)^37,38^. Comparing with the fruitful chemistry of active Huisgen 1,4-dipoles and the enamino esters, the reactivity of this kind of active 1,3-dipoles is still not broadly investigated. In order to develop the synthetic applications of this new kind azomethine ylides for diverse nitrogen-containing heterocyclic systems and continue our aim to explore more efficient and sustainable synthetic methodology for biologically important spiro compounds^39–44^, herein we wish to report the interesting regioselectivity and diastereoselectivity of three-component reaction of α-amino acids, dialkyl acetylenedicarboxylates and arylideneindane-1,3-diones.

## Results and Discussions

**Figure 2:**
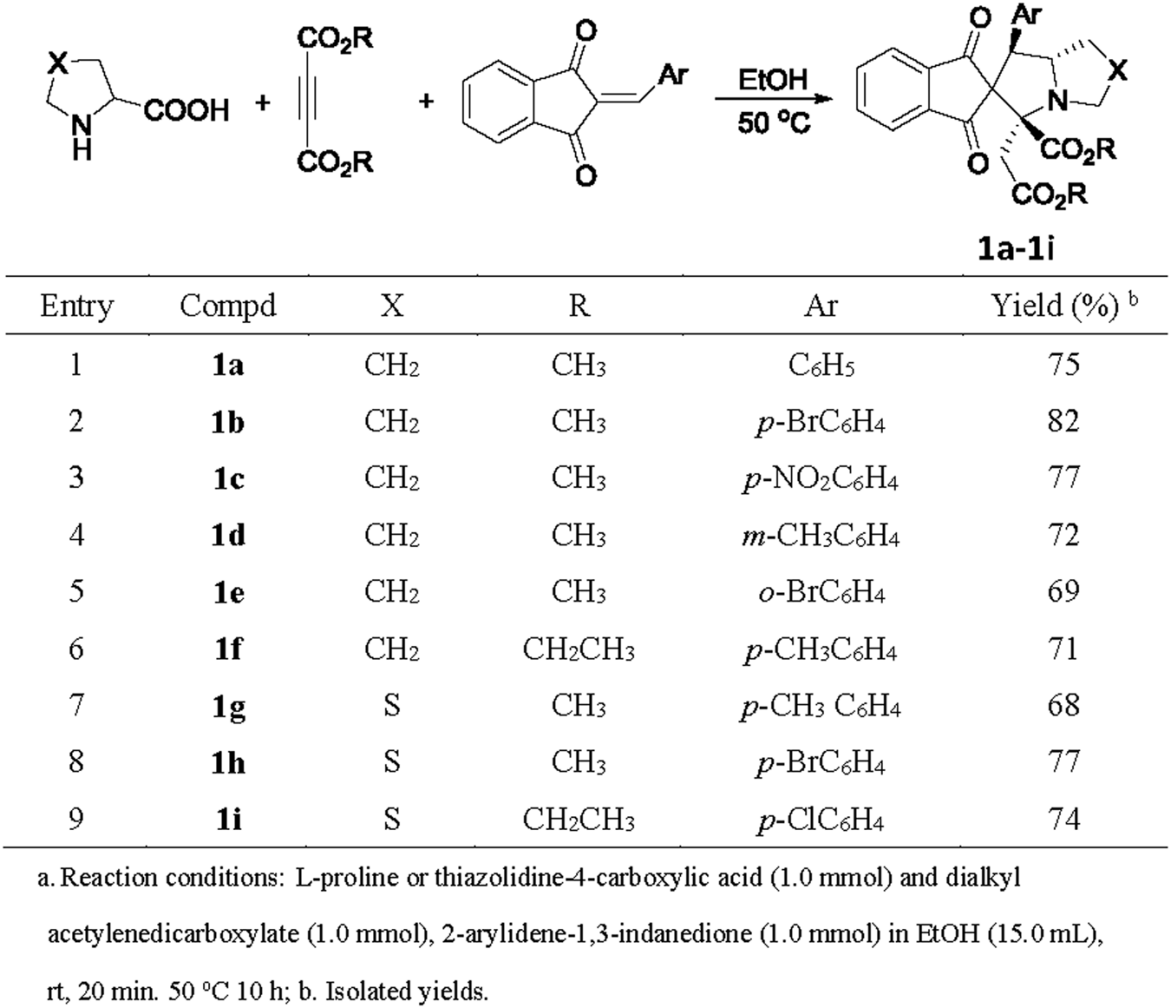
Synthesis of spiro[indene-2,2′-pyrrolizines]**1a-1i** ^**a**^.

**Figure 3:**
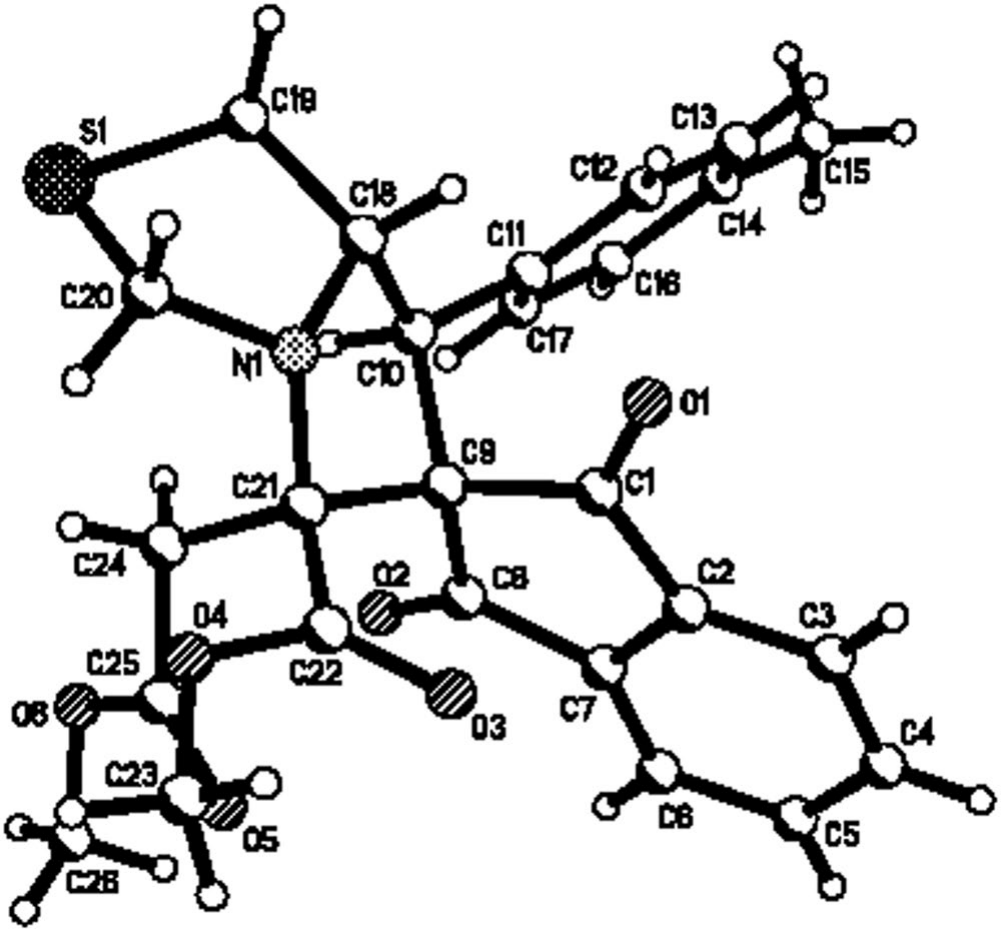
Single crystal structure of compound **1g**.

According to our previously established reaction procedure for the 1,3-dipolar cycloaddition of azomehine ylides with maleimides or 3-phenacylideneoxindolines, the three-component reaction of L-proline, dialkyl acetylenedicarboxylate and 2-arylidene-1,3-indanedione was carried out in ethanol at 50 °C for ten hours. After workup, the expected spiro[indene-2,2′-pyrrolizines] **1a-1f** were predominately produced in 69–78% yields (Fig. 2, entries 1–6). The similar reaction with thiazolidine-4-carboxylic acid also proceeded smoothly to give corresponding spiro [indene-2,6′-pyrrolo[1,2-c]thiazoles] **1g-1i** in good yields (Fig. 2, entries 7–9). The structures of obtained spiro compounds **1a-1i** were fully characterized by HRMS, IR, ^1^H and ^13^C NMR spectra. Because the obtained compounds have three chiral carbon atoms, several diastereoisomers might be formed in the reaction. We were pleased to find that only one diastereoisomer were predominately formed in the reaction by TLC monitoring and the spectroscopy. The single crystal structure of the spiro compound **1g** was determined by X-ray diffraction method (Fig. 3). It can be seen that the aryl group and the methoxycarbonyl group exist in *cis*-position of the newly-formed pyrrolidine ring. The methoxycarbonylmethyl group stands at *trans*-configuration. On the basis of the spectroscopy and single crystal structure, we could conclude that the spiro compounds **1a-1i** are all belonging to this kind of configuration and this 1,3-dipolar cycloaddition reaction showed very high disatereoselectivity.

**Figure 4:**
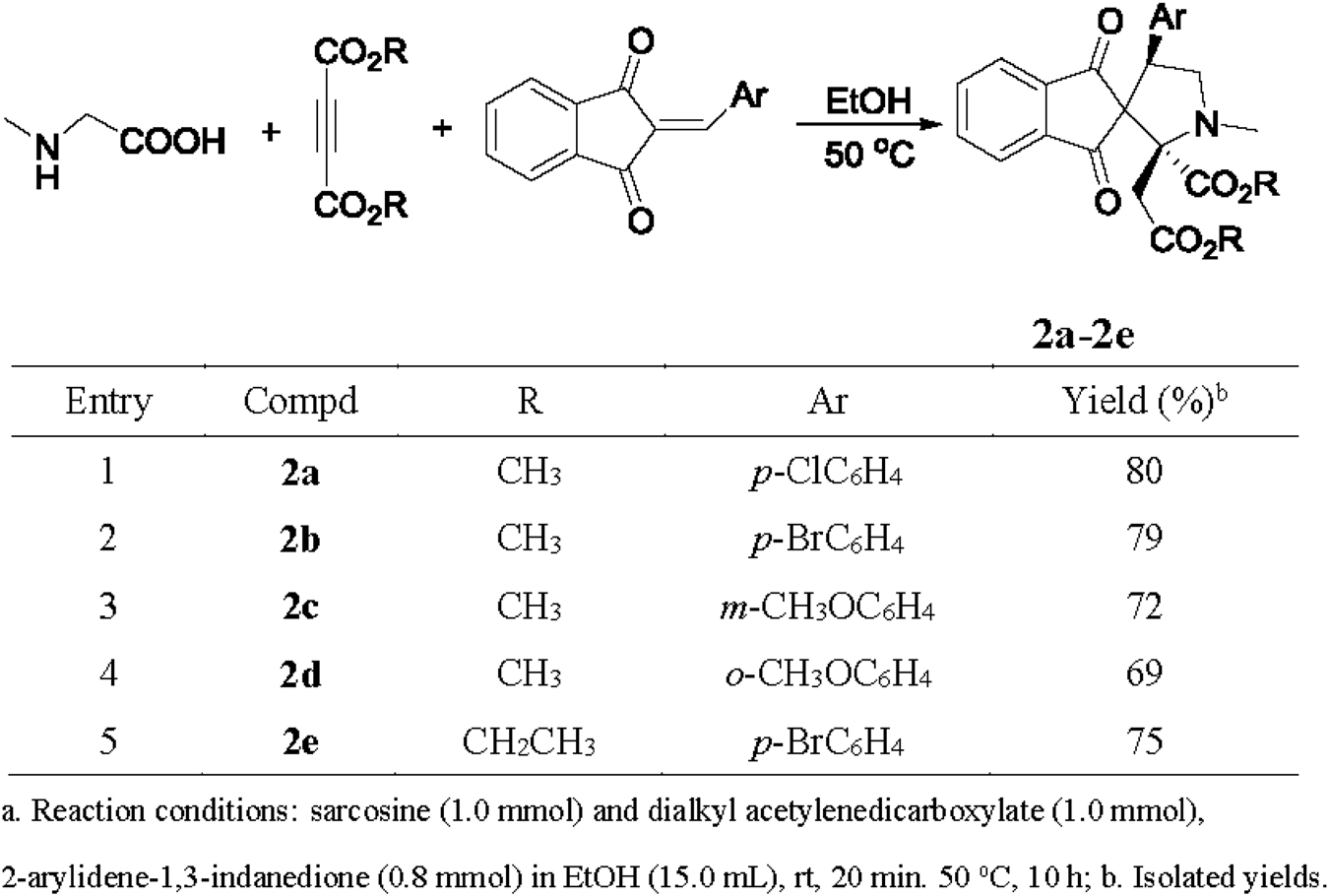
Synthesis of spiro[indene-2,3′-pyrrolidines] **2a-2e** ^**a**^.

**Figure 5:**
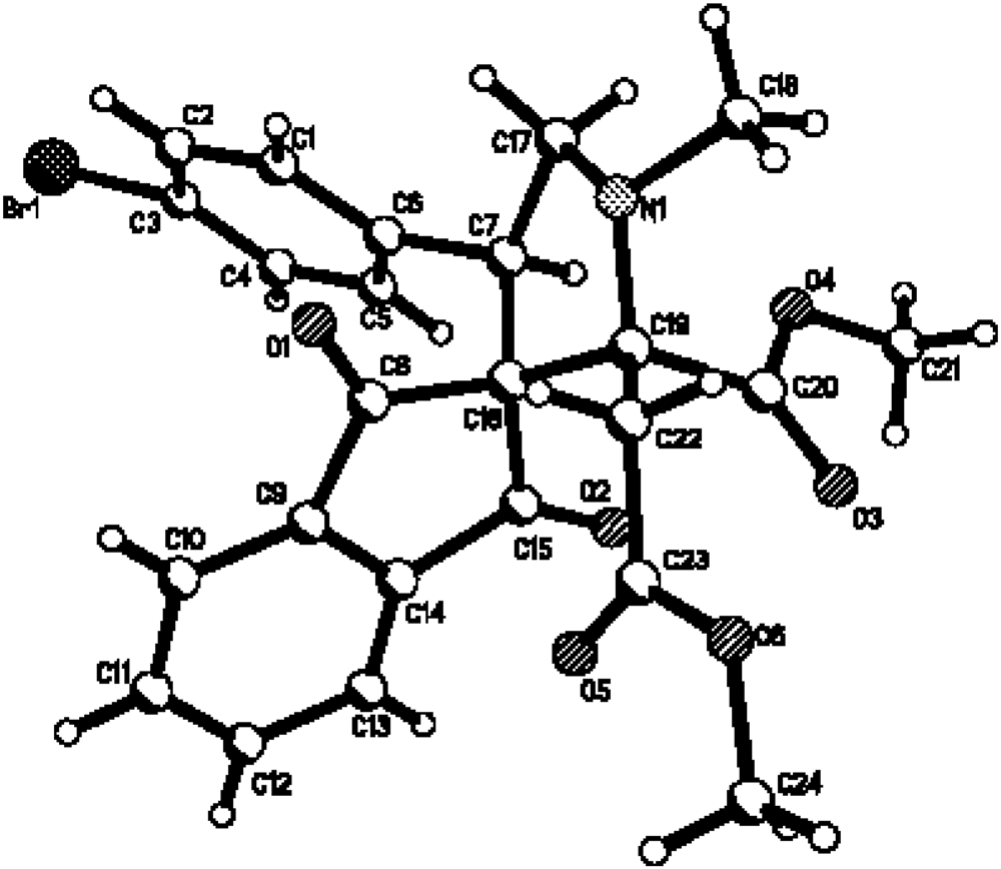
Single crystal structure of compound **2b**.

Under same reaction conditions, sarcosine was also employed in the reaction to give functionalized spiro[indene-2,3′-pyrrolidines] **2a-2e** in satisfactory yields (Fig. 4). The five compounds **2a-2e** displayed very similar^1^H NMR spectra, in which only one set of the signs for the characteristic groups in the molecule was observed. This clearly showed only one diastereroisomer existing in the obtained products **2a-2e**. As for an example, the compound **2a** showed three singlets at 3.95, 3. 36, 2.51 ppm for the two methoxy groups and one methylamino group. The CH_2_ unit in the newly-formed pyrrolidine ring reveals two diasterotopic doublets at 2.99, 2.91 ppm with *J* = 16.4 Hz. The chain CH_2_ group connecting to ester scaffold also gives two mixed peaks at 4.39–4.35 and 3.79–3.75 ppm. The single crystal structures of the compounds **2b** (Fig. 5) and **2c** (SI, Fig. S1) indicated that the aryl group and the methoxycarbonylmethyl group exist at *cis*-position in the newly-formed pyrrolidine ring. However, the methoxycarbonyl group stands at opposite direction. This result indicated the configuration of the compound **2a-2e** is different to that of the above prepared spiro[indene-2,2′-pyrrolizines]**1a-1i**.

**Figure 6:**
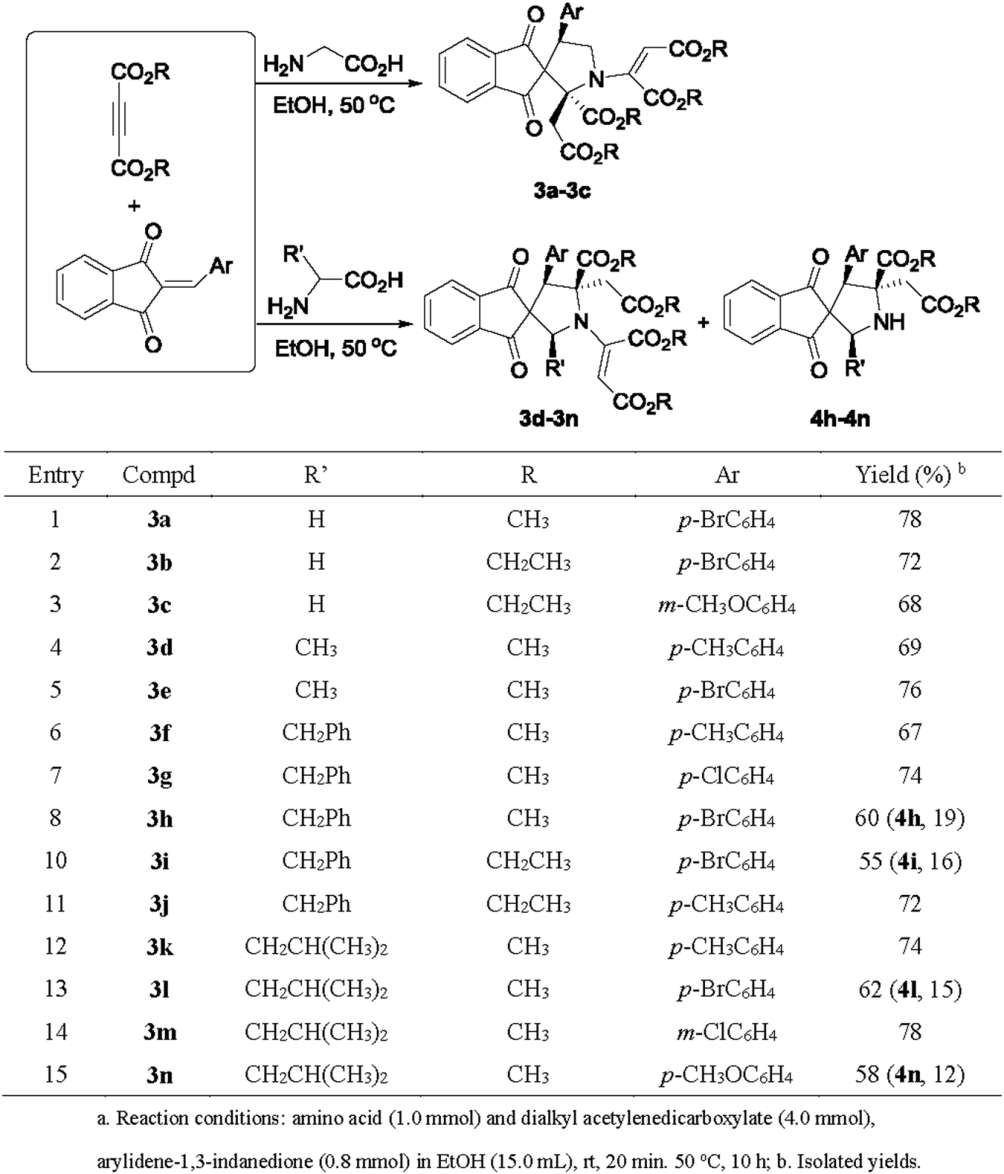
Synthesis of spiro[indene-2,3′-pyrrolidines] **3a-3n** ^**a**^.

**Figure 7:**
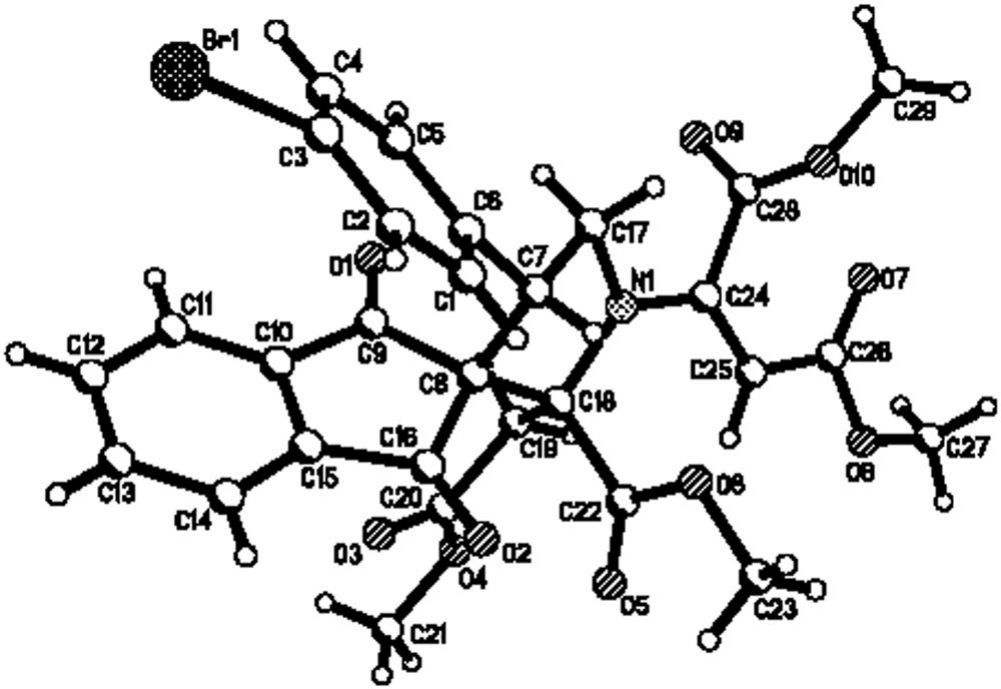
Single crystal structure of compound **3a**.

**Figure 8:**
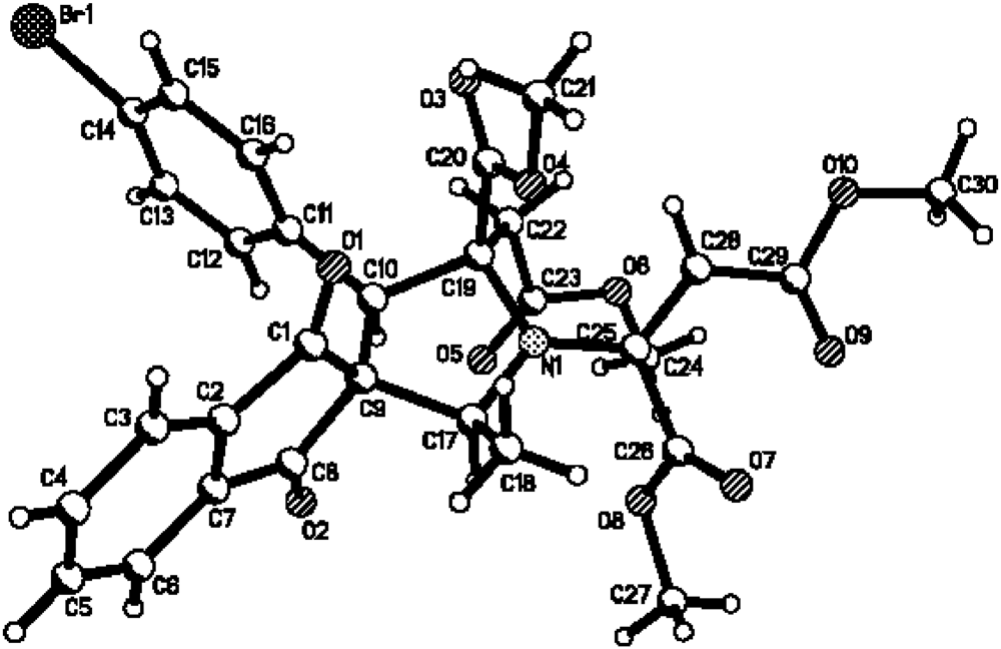
Single crystal structure of compound **3e**.

When the chain amino acids were employed in the three-component reactions, versatile products with interesting regioselectivity and diastereoselectivity were successfully obtained. At first, the reaction with glycine gave the expected spiro[indene-2,3′-pyrrolidines] **3a-3c** in good yields (Fig. 6). The spectroscopy and single crystal structures indicated that an additional moiety of dialkyl maleat was connected to the nitrogen-atom of pyrrolidine, which obviously came from the addition of free amino group of initially formed pyrroline ring to second molecule of dialkyl acetylenedicarboxylate. The same phenomena is also happened in our previously reported reactions. The single crystal structures of **3a** (Fig. 7) and **3c** (SI, Fig. S2) showed that the aryl group and the alkoxycarbonylmethyl group exist *cis*-position in the newly-formed ring of pyrrolidine. Thus, the spiro compounds **3a-3c** have the same configuration to that of the above mentioned products **2a-2e**. Secondly, the similar reactions with other primary amino acids including alanine, phenylalanine and leucine afforded spiro[indene-2,3′-pyrrolidines] **3d-3n**. It should be pointed out that the reactions of phenylalanine and leucine also gave the spiro[indene-2,3′-pyrrolidines] **4 h**, **4i**, **4 l** and **4n** as minor products. This result might be due to the steric hindrance of more substituents in these reactions. Although the spectroscopy of compounds **3d-3n** are very similar to that of compounds **3a-3c**, the single crystal structures of compounds **3e** (Fig. 8), **3h** and **3m** (Si, Figs S3, S4) clearly revealed that they are belonging to two kinds of structural isomers and diastereoisomers. In the compounds **3a-3c**, the aryl group and the alkoxycarbonyl group *exist* in 2,4-*trans*-position and the 1,3-dipolar cycloaddition was finished in so-called head-to-tail reaction pattern. In the compounds **3d-3n**, the aryl group and the alkoxycarbonyl group exist in 2,3-*cis*-position. Here, the alkyl groups play an important role for the regioselectivity and diasteroselectivity in the 1,3-dipolar cycloaddition.

**Figure 9:**
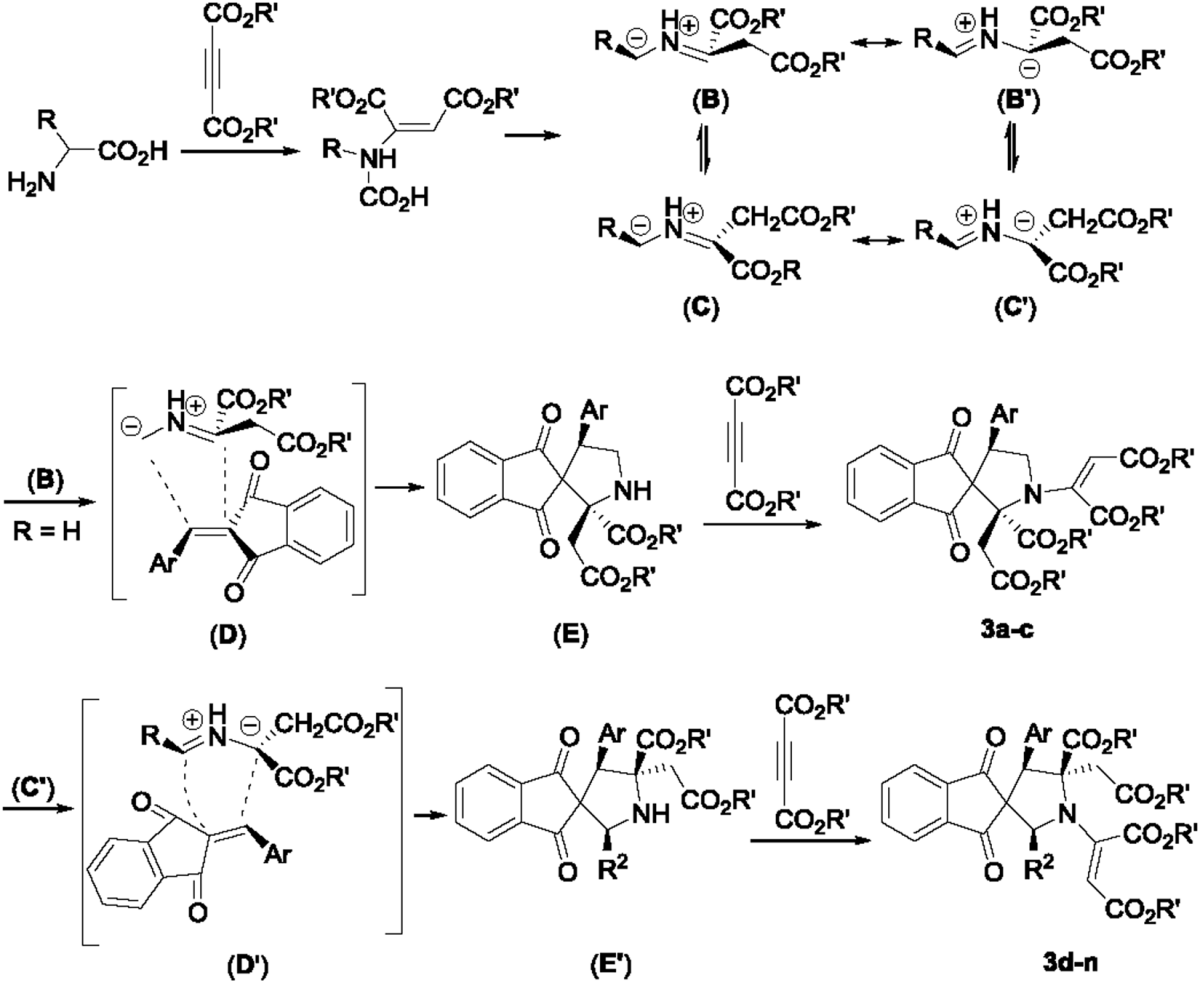
Proposed formation mechanism for spiro[indene-2,3′-pyrrolidines] **3a-3n**.

At present, the exact addition mechanism and the controlling the effect on stereochemistry is not very clear. For explaining the reaction formation, a plausible 1,3-dipolar cycloaddition reaction process was proposed in Fig. 9. At first, the amino group of amino acid added to the triple bond of the acetylenedicarboxylate to give the adduct (**A**), which was transferred to a 1,3-dipolar azomethine ylide (**B**) by elimination of carbon dioxide. The azomethine ylide (**B**) not only has different conformations (such as **C**), but also has several resonance forms (**B**′), which enabled the 1,3-dipolar cycloaddition with variable regioselectivity and diastereoselectivity. Because of the two stronger electron-withdrawing ester groups in the molecule, a isomeric azomethine ylide (**B′**) could be formed by prototropic shift of the imine. It was widely accepted that 1,3-diploe with stable *anti*-conformation reacted with the dipolarophiles according to the *endo*-approach manner in the concerted 1,3-dipolar cycloaddition reaction^46,47^. When glycine was used in the reaction (R=H), azomethine ylide (**B**) added to 2-arylidene-1,3-indanedione through the transition state (**D**) to give the spiro[indene-2,3′-pyrrolidine] (**E**). Then, it reacted with another molecular acetylenedicarboxyalte to give the spiro compounds **3a-3c**. When the substituted amino acids were used in the reaction, the cycloaddition of the azomethine ylide (**C′**) to 2-arylidene-1,3-indanedione according to another transition state (**D**′) afforded structurally isomeric spiro[indene-2,3′-pyrrolidines] (**E′**), which could be separated as the stable products **4h-4n** in some cases. At last, reaction of (**E′**) with excess of acetylenedicarboxylate resulted in the spiro compounds **3d-3n**. The azomethine ylides generated from sarcosine and the cyclic amino acids also proceeded with the similar 1,3-dipolar cycloaddition reaction process.

## Conclusion

In summary, we have investigated the three-component reaction of α-amino acid, dialkyl acetylenedicarboxylate and 2-arylidene-1,3-indanedione and developed new applications of the new kind of azomethine ylide generated *in situ* from addition reaction of α-amino acid with dialkyl acetylenedicarboxylate. This reaction provided an regioselective and diastereoselective protocol for the synthesis of diverse spiro[indene-2,3′-pyrrolidines], spiro[indene-2,2′-pyrrolizines] and spiro[indene-2,6′-pyrrolo[1,2-c]thiazoles]. This domino 1,3-dipolar cycloaddition has the advantages of using readily available reagents, mild reaction condition, high diastereoselectivity and interesting molecular diversity, which would be found high potential applications in the heterocyclic synthesis.

## Methods

### Materials

All reactions were performed in atmosphere unless noted. All reagents were commercially available and use as supplied without further purification. NMR spectra were collected on either an Agilent DD2400 MHz spectrometer or a Bruker AV-600 MHz spectrometer with internal standard tetramethylsilane (TMS) and signals as internal references, and the chemical shifts (δ) were expressed in ppm. High-resolution Mass (ESI) spectra were obtained with Bruker Micro-TOF spectrometer. The Fourier transform infrared (FTIR) samples were prepared as thin films on KBr plates, and spectra were recorded on a Bruker Tensor 27 spectrometer and are reported in terms of frequency of absorption (cm^−1^). X-ray data were collected on a Bruker Smart APEX-2 CCD diffractometer.

### General procedure for the three-component reaction of secondary α-amino acids with dialkyl acetylenedicarboxylate and 2-arylidene-1,3-indanediones

A mixture of L-proline or thiazolidine-4-carboxylic acid, or sarcosine (1.0 mmol) and dialkyl acetylenedicarboxylate (1.0 mmol) in ethanol (15.0 mL) was stirred at room temperature for twenty minutes. Then, 2-arylidene-1,3-indanedione (0.8 mmol) was added. The mixture was stirred at about 50 °C for ten hours. The solvent was removed at reduced pressure by rotator evaporation, the residue was subjected to preparative thin-layer chromatography (20 × 30 cm^2^ Silica gel GF254) with a mixture of light petroleum and ethyl acetate (V/V = 2.5:1) as eluent to give the pure products **1a-1i** and **2a-2e**.

### General procedure for the three-component reaction of primary α-amino acids with dialkyl acetylenedicarboxylate and 2-arylidene-1,3-indanediones

A mixture of glycine (or alanine, phenylalanine, leucine, 1.0 mmol) and dialkyl acetylenedicarboxylate (4.0 mmol) in ethanol (15.0 mL) was stirred at room temperature for twenty minutes. Then, 2-arylidene-1,3-indanedione (0.8 mmol) was added. The mixture was stirred at about 50 °C for ten hours. The solvent was removed at reduced pressure by rotator evaporation, the residue was subjected to preparative thin-layer chromatography (20 × 30 cm^2^ Silica gel GF254) with a mixture of light petroleum and ethyl acetate (V/V = 2.5:1) as eluent to give the pure product **3a-3n**.

### Accession codes

Crystallographic data **1g** (CCDC 1518094), **2b** (CCDC 1518095), **2c** (CCDC 1518096), **3a** (CCDC 1518097), **3c** (CCDC 1518098), **3e** (CCDC 1518099), **3h** (CCDC 1518100), **3m** (CCDC 1518101) have been deposited at the Cambridge Crystallographic Database Centre (http://www.ccdc.cam.ac.uk).

## Electronic supplementary material

Supplementary Information
