# Supplementary material for: Regioselectivity and diastereoselectivity of three-component reaction of α-amino acid, dialkyl acetylenedicarboxylates and 2-arylidene-1,3-indanediones
Source: Sci Rep. 2017 Sep 29;7:12418. doi: 10.1038/s41598-017-12361-z (PMC5622111; doi:10.1038/s41598-017-12361-z)

Regioselectivity and diastereoselectivity of three-component reaction of α-amino acid, dialkyl acetylenedicarboxylates and 2-arylidene-1,3-indanediones

Liang Chen, Jing Sun*, Ying Huang, Yu Zhang, Chao-Guo Yan*

Supporting Information

Single crystal sctructural figures (Fig.s1-s4) **2**

^{1}H NMR, ^{13}C NMR and HRMS spectra **3-34**

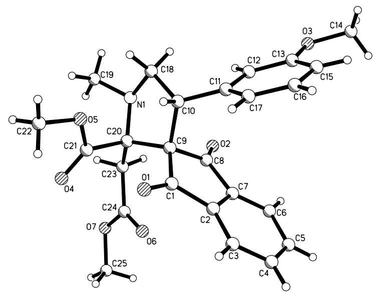

Fig. s1 Single crystal structure of compound **2c**

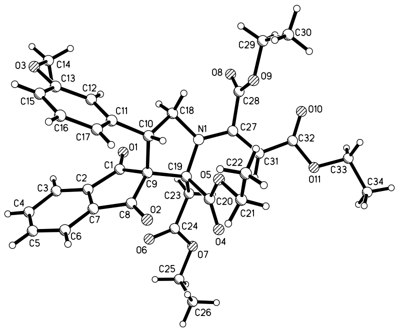

Fig. s2 Single crystal structure of compound **3c**

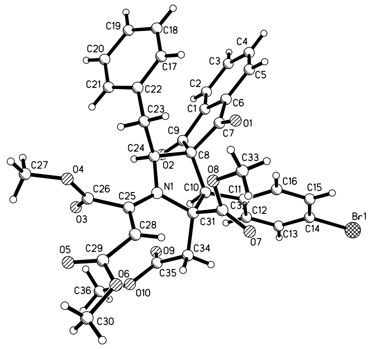

Fig. s3 Single crystal structure of compound **3h**

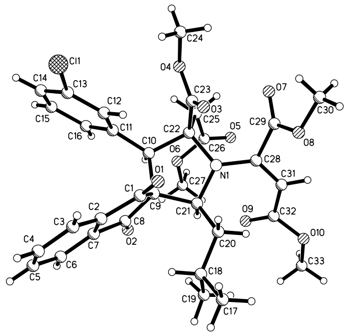

Fig. s4 Single crystal structure of compound **3m**

Methyl 3'-(2-methoxy-2-oxoethyl)-1,3-dioxo-1'-phenyl-1,3,5',6',7',7a'-hexahydro-1'H,3'H-spiro[indene-2,2'-pyrrolizine]-3'-carboxylate (1a): white solid, 75%, m.p. 158-160℃; ^{1}H NMR (400 MHz, $CDCl_3$) δ: 7.75 (d, *J* = 7.2 Hz, 1H, ArH), 7.62-7.55 (m, 2H, ArH), 7.53-7.50 (m, 1H, ArH), 7.04-6.99 (m, 2H, ArH), 6.95-6.93 (m, 3H, ArH), 4.46 (s, 1H, CH), 4.21 (d, *J* = 9.6Hz, 1H, CH), 3.93 (s, 3H, OCH_3), 3.33 (s, 3H, OCH_3), 3.09-3.02 (m, 1H, CH), 2.95 (d, *J* = 16.0Hz, 1H, CH), 2.90 (brs, 1H, CH), 2.86 (brs, 1H, CH), 2.27 (brs, 1H, CH), 2.19-2.12 (m, 1H, CH), 1.85 (brs, 1H, CH), 1.79-1.75 (m, 1H, CH); ^{13}C NMR (100 MHz, $CDCl_3$) δ: 202.4, 196.4, 170.1, 170.0, 141.1, 140.3, 134.0, 133.7, 127.9, 127.5, 127.1, 126.6, 121.7, 121.5, 72.6, 66.7, 56.2, 51.4, 50.2, 45.8, 39.0, 29.4, 28.8, 28.7; IR(KBr) υ: 3008, 2945, 2830, 1727, 1701, 1596, 1456, 1334, 1208, 1180, 1051, 1023, 1009, 976, 888, 779 cm^{-1}; MS (*m/z*): HRMS (ESI) Calcd. for $C_{26}H_{26}NO_6$ ($[M+H]^+$): 448.1755, found: 448.1772.

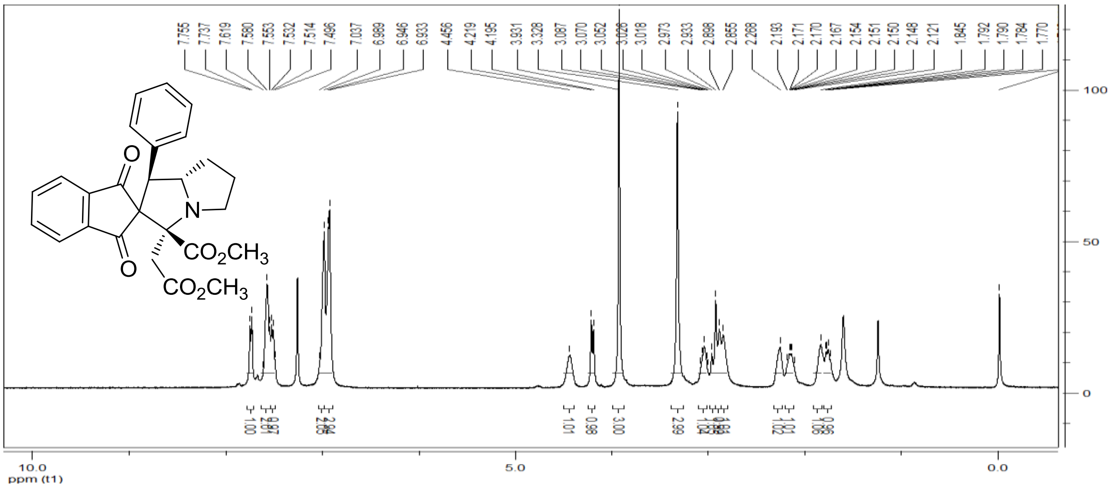

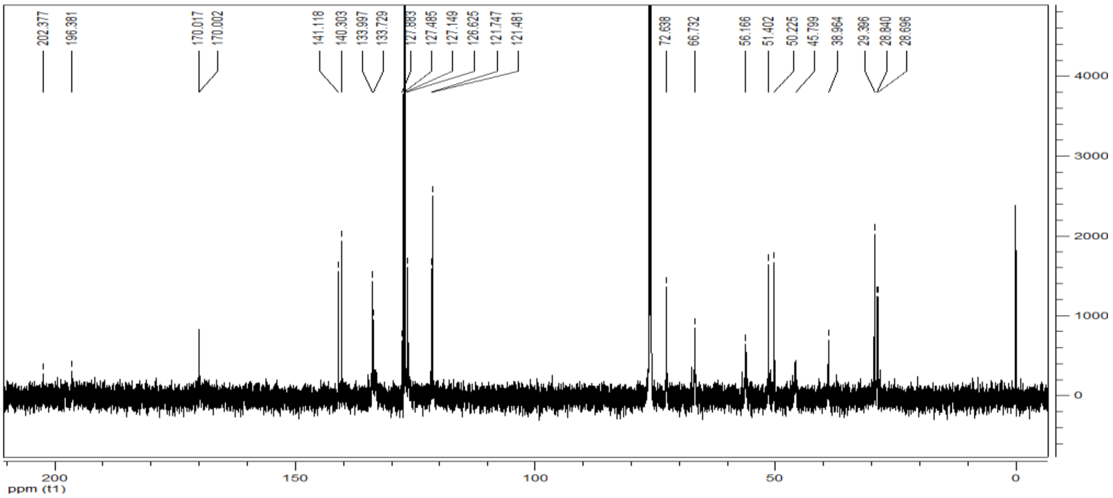

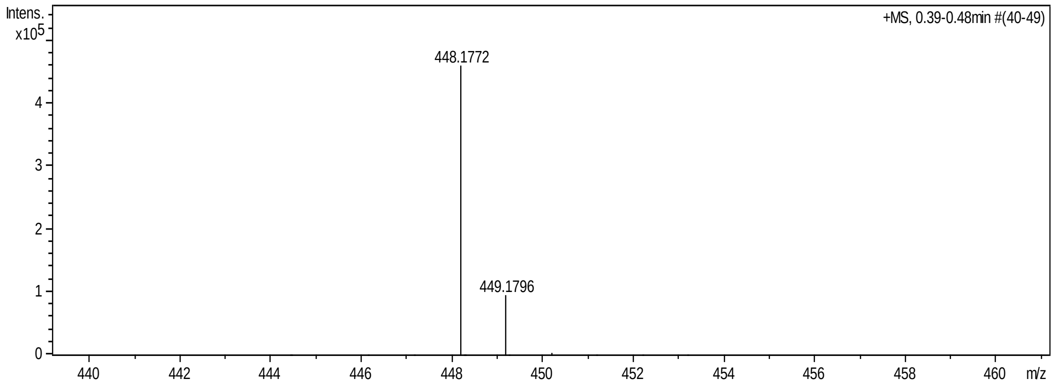

Methyl 1'-(4-bromophenyl)-3'-(2-methoxy-2-oxoethyl)-1,3-dioxo-1,1',3,3',5',6',7',7a'-octahydrospiro[indene-2,2'-pyrrolizine]-3'-carboxylate (1b): white solid, 82%, m.p. 138-140℃; ^{1}H NMR (600 MHz, $CDCl_3$) δ: 7.77 (d, J = 7.8 Hz, 1H, ArH), 7.65-7.62 (m, 2H, ArH), 7.60-7.58 (m, 1H, ArH), 7.09 (d, J = 8.4 Hz, 2H, ArH), 6.89 (d, J = 8.4 Hz, 2H, ArH), 4.41 (brs, 1H, CH), 4.15 (d, J = 10.2 Hz, 1H, CH), 3.91 (s, 3H, OCH_3), 3.34 (s, 3H, OCH_3), 3.06 (brs, 1H, CH), 2.92-2.90 (m, 3H, CH, CH_2), 2.30-2.26 (m, 1H, CH), 2.17-2.11 (m, 1H, CH), 1.87-1.82 (m, 1H, CH), 1.78-1.71 (m, 1H, CH); ^{13}C NMR (150 MHz, $CDCl_3$) δ: 198.8, 197.3, 171.4, 171.0, 142.1, 141.2, 135.4, 135.1, 133.6, 131.3, 130.2, 122.8, 122.6, 121.6, 73.6, 73.4, 67.9, 56.2, 52.4, 51.2, 46.8, 40.0, 30.3, 29.8; IR(KBr) υ: 2943, 2857, 1730, 1592, 1486, 1445, 1350, 1264, 1220, 1179, 1125, 1080, 1001, 895, 856, 826, 760, 737 cm^{-1}; MS (m/z): HRMS (ESI) Calcd. for $C_{26}H_{25}BrNO_6$ ($[M+H]^+$): 526.0860, found: 526.0887.

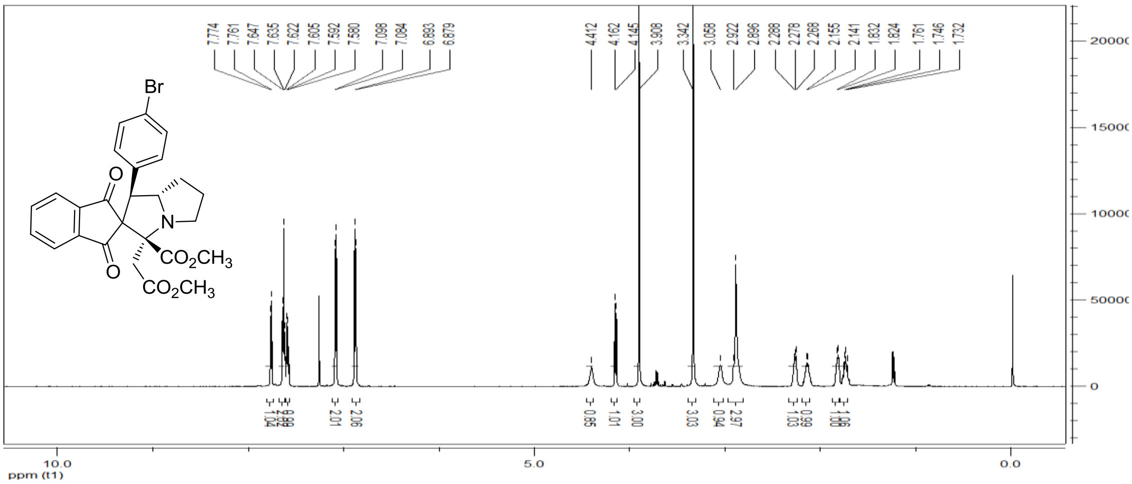

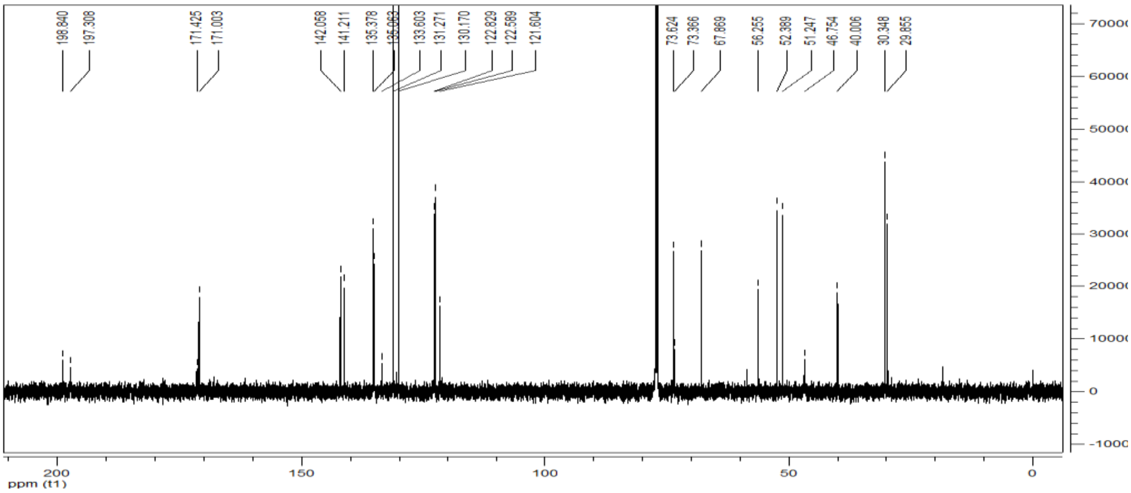

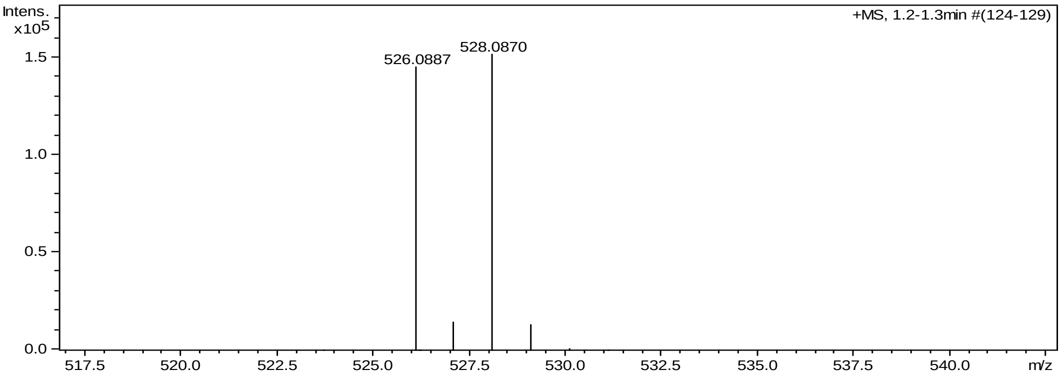

Methyl 3'-(2-methoxy-2-oxoethyl)-1'-(4-nitrophenyl)-1,3-dioxo-1,1',3,3',5',6',7',7a'-octahydrospiro[indene-2,2'-pyrrolizine]-3'-carboxylate (1c): white solid, 77%, m.p. 174-176℃; ^{1}H NMR (400 MHz, $CDCl_3$) δ: 7.84 (d, J = 8.4 Hz, 2H, ArH), 7.78 (d, J = 7.6 Hz, 1H, ArH), 7.65-7.62 (m, 2H, ArH), 7.60-7.56 (m, 1H, ArH), 7.21 (d, J = 8.4 Hz, 2H, ArH), 4.48 (brs, 1H, CH), 4.30 (d, J = 9.6 Hz, 1H, CH), 3.91 (s, 3H, OCH_3), 3.36 (s, 3H, OCH_3), 3.05 (s, 1H, CH), 2.89 (brs, 3H, CH, CH_2), 2.35-2.27 (m, 1H, CH), 2.22-2.13 (m, 1H, CH), 1.87-1.73 (m, 2H, CH_2); ^{13}C NMR (100 MHz, $CDCl_3$) δ: 194.2, 170.9, 170.8, 141.9, 141.0, 135.6, 135.3, 135.3, 129.5, 123.2, 122.8, 122.7, 73.6, 67.9, 56.1, 52.4, 51.3, 46.6, 39.9, 39.9, 30.3, 29.8; IR(KBr) υ: 3073, 2953, 2860, 1712, 1599, 1521, 1439, 1349, 1264, 1218, 1177, 1118, 1049, 1007, 973, 899, 854, 798, 766, 731 cm^{-1}; MS (m/z): HRMS (ESI) Calcd. for $C_{26}H_{25}N_2O_8$($[M+H]^+$): 493.1605, found: 493.1612.

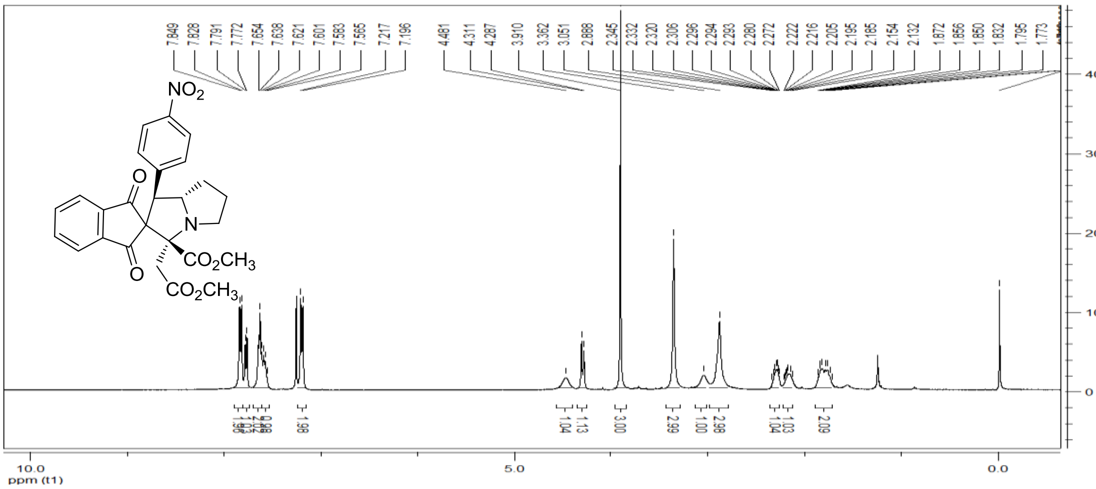

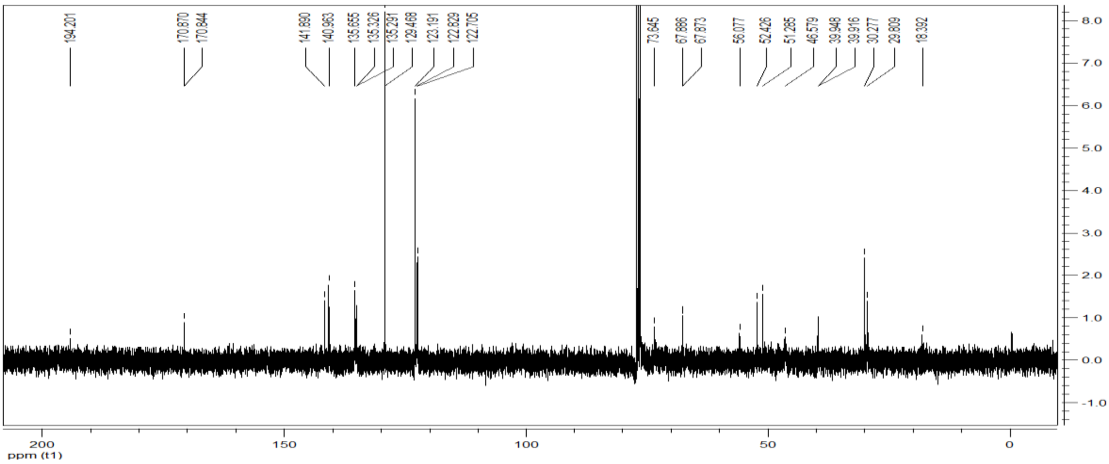

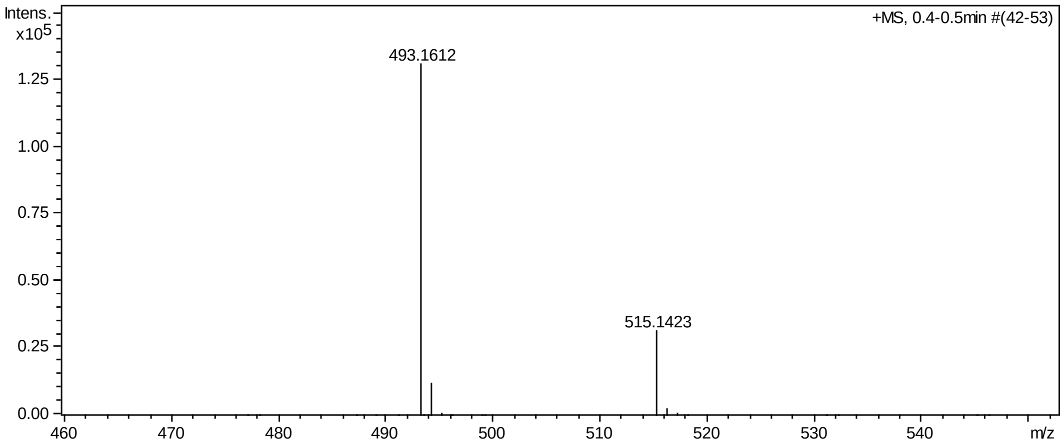

Methyl 3'-(2-methoxy-2-oxoethyl)-1,3-dioxo-1'-(m-tolyl)-1,1',3,3',5',6',7',7a'-octahydrospiro[indene-2,2'-pyrrolizine]-3'-carboxylate (1d): white solid, 72%, m.p. 154-156℃; ^{1}H NMR (600 MHz, $CDCl_3$) δ: 7.75 (d, *J* = 7.8 Hz, 1H, ArH), 7.58-7.57 (m, 2H, ArH), 7.52-7.51 (m, 1H, ArH), 6.85-6.84 (m, 2H, ArH), 6.76 (s, 1H, ArH), 6.72 (d, *J* = 6.0 Hz, 1H, ArH), 4.42 (s, 1H, CH), 4.16 (d, *J* = 9.6 Hz, 1H, CH), 3.93 (s, 3H, OCH_3), 3.33 (s, 3H, OCH_3), 3.05 (brs, 1H, CH), 2.96 (d, *J* = 16.2 Hz, 1H, CH), 2.89-2.82 (m, 2H, CH_2), 2.26 (brs, 1H, CH), 2.16-2.15 (m, 1H, CH), 2.06 (s, 3H, CH_3), 1.85 (brs, 1H, CH), 1.77-1.76 (m, 1H, CH); ^{13}C NMR (100 MHz, $CDCl_3$) δ: 199.0, 197.6, 171.8, 171.1, 142.2, 141.4, 137.6, 134.8, 134.6, 129.4, 128.2, 128.0, 125.3, 122.6, 122.3, 73.5, 67.5, 57.3, 52.3, 51.1, 46.6, 40.0, 30.4, 29.9, 21.0; IR(KBr) υ: 3005, 2952, 2868, 1738, 1703, 1596, 1435, 1356, 1257, 1208, 1174, 1091, 1051, 1009, 976, 888, 848, 779, 734 cm^{-1}; MS (*m/z*): HRMS (ESI) Calcd. for $C_{27}H_{28}NO_6$ ($[M+H]^+$): 462.1911, found: 462.1909.

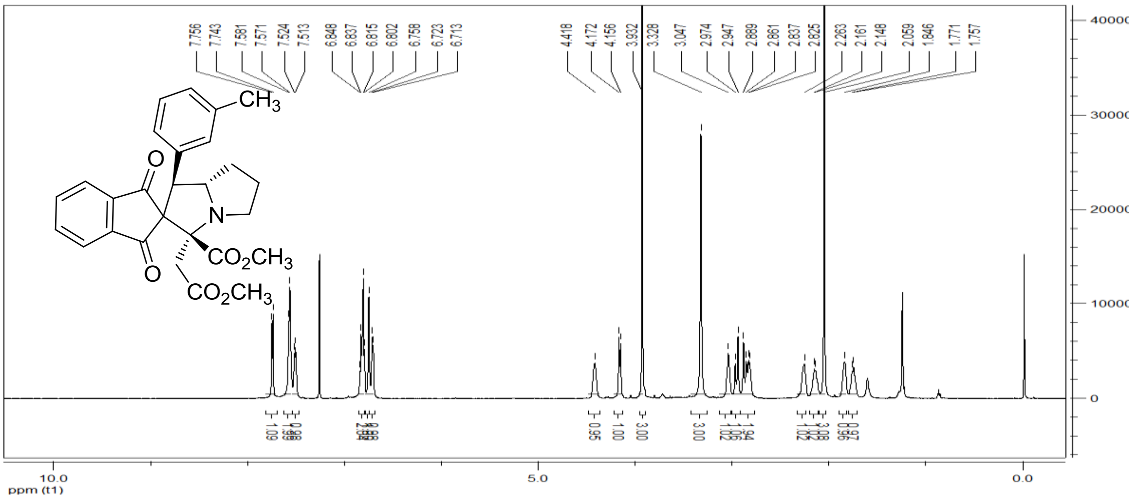

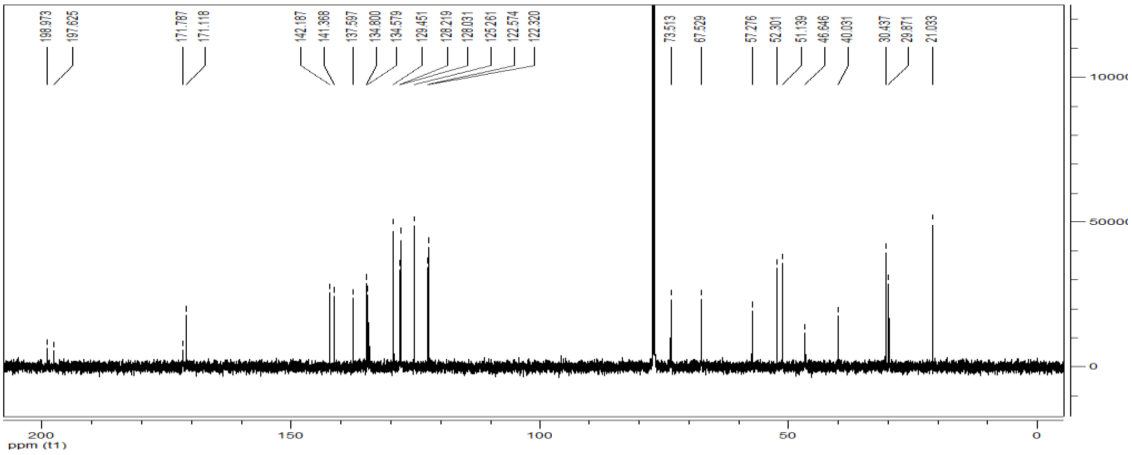

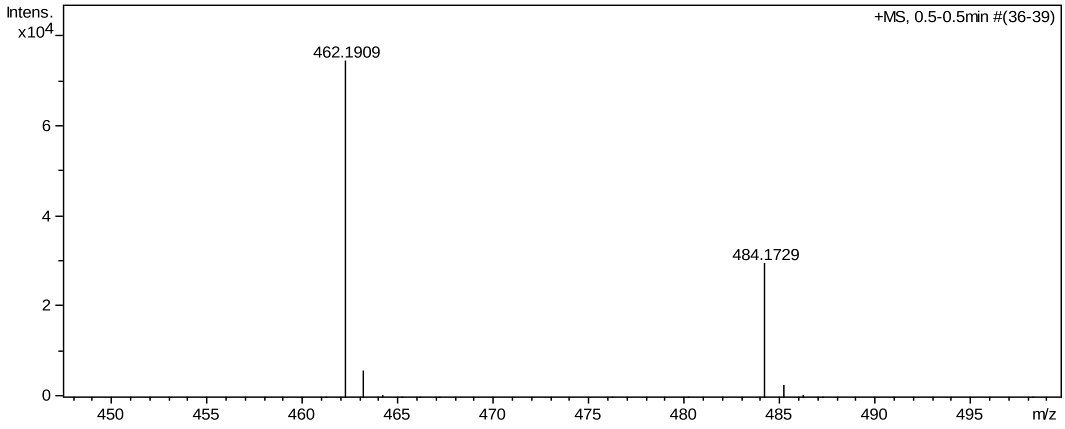

Methyl 1'-(2-bromophenyl)-3'-(2-methoxy-2-oxoethyl)-1,3-dioxo-1,1',3,3',5',6',7',7a'-octahydrospiro[indene-2,2'-pyrrolizine]-3'-carboxylate (1e): white solid, 69%, m.p. 172-174℃; ^{1}H NMR (400 MHz, $CDCl_3$) δ: 7.80 (d, *J* = 7.6 Hz, 1H, ArH), 7.62-7.57 (m, 2H, ArH), 7.54-7.50 (m, 1H, ArH), 7.25-7.20 (m, 2H, ArH), 7.01 (t, *J* = 7.6 Hz, 1H, ArH), 6.78 (t, *J* = 7.2 Hz, 1H, ArH), 5.13 (d, *J* = 9.2 Hz, 1H, CH), 4.26 (brs, 1H, CH), 3.99 (s, 3H, OCH_3), 3.34 (s, 3H, OCH_3), 3.08-3.04 (m, 2H, CH_2), 2.96-2.92 (m, 1H, CH), 2.85-2.81 (m, 1H, CH), 2.30-2.24 (m, 1H, CH), 2.17-2.08 (m, 1H, CH), 1.86-1.74 (m, 2H, CH_2); ^{13}C NMR (100 MHz, $CDCl_3$) δ: 199.4, 199.1, 171.1, 171.0, 141.7, 141.1, 134.9, 134.5, 133.1, 129.3, 128.6, 127.0, 126.5, 122.8, 122.4, 73.8, 69.7, 54.2, 52.4, 51.1, 46.9, 39.8, 39.8, 30.2, 29.5; IR(KBr) υ: 2948, 2869, 1738, 1704, 1594, 1466, 1435, 1356, 1257, 1208, 1171, 1052, 1016, 975, 895, 750 cm^{-1}; MS (*m/z*): HRMS (ESI) Calcd. for $C_{26}H_{25}BrNO_6$($[M+H]^+$): 526.0860, found: 526.0887.

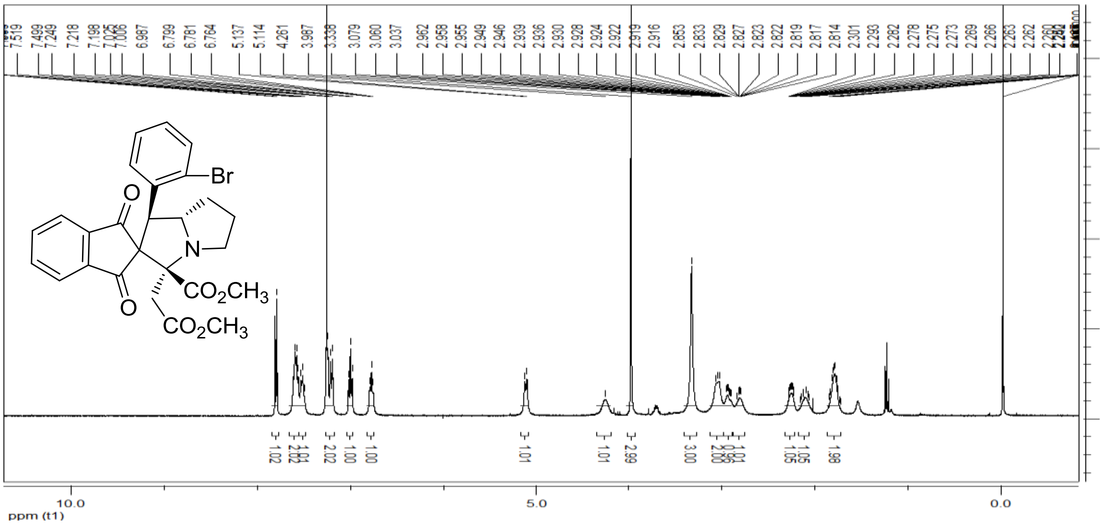

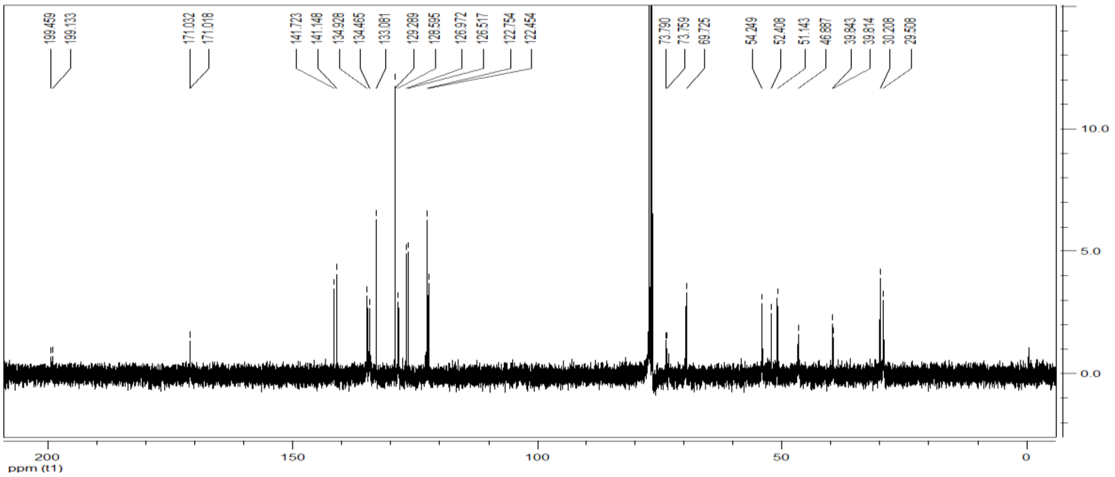

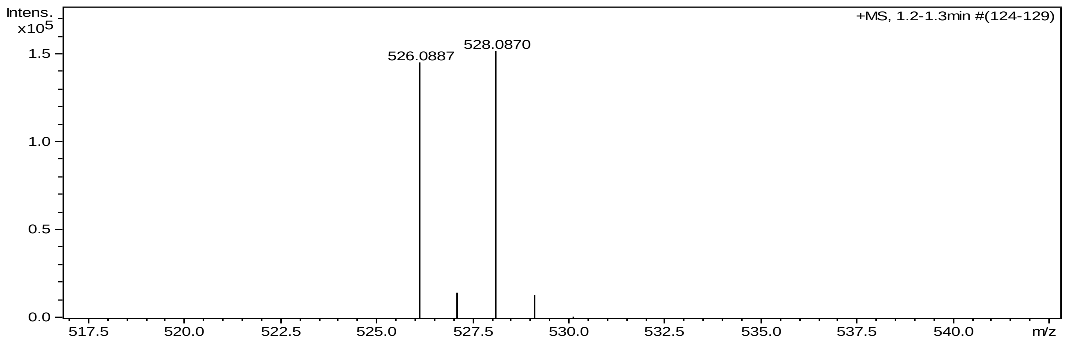

Ethyl 3'-(2-ethoxy-2-oxoethyl)-1,3-dioxo-1'-(p-tolyl)-1,1',3,3',5',6',7',7a'-octahydrospiro-[indene-2,2'-pyrrolizine]-3'-carboxylate (1f): white solid, 71%, m.p. 134-136℃; ^{1}H NMR (600 MHz, $CDCl_3$) δ: 7.74 (d, *J* = 7.2 Hz, 1H, ArH), 7.61-7.56 (m, 2H, ArH), 7.53-7.51 (m, 1H, ArH), 6.88 (d, *J* = 7.8 Hz, 2H, ArH), 6.75 (d, *J* = 7.8 Hz, 2H, ArH), 4.46-4.40 (m, 2H, CH_2), 4.39-4.35 (m, 1H, CH), 4.22 (d, *J* = 9.6 Hz, 1H, CH), 3.84-3.73 (m, 2H, CH_2), 3.05 (brs, 1H, CH), 2.93-2.86 (m, 2H, CH_2), 2.31-2.23 (m, 1H, CH), 2.17-2.14 (m, 1H, CH), 2.07 (s, 3H, CH_3), 1.83-1.75 (m, 2H, CH_2), 1.59 (brs, 1H, CH), 1.38 (t, *J* = 7.2 Hz, 3H, CH_3), 0.99 (t, *J* = 6.6 Hz, 3H, CH_3); ^{13}C NMR (100 MHz, $CDCl_3$) δ: 170.6, 142.3, 141.4, 137.1, 134.8, 134.4, 128.7, 128.3, 122.6, 122.4, 73.4, 67.8, 61.5, 60.0, 56.9, 40.4, 30.3, 29.8, 29.6, 20.7, 14.0, 13.8; IR(KBr) υ: 2972, 2931, 2869, 1736, 1594, 1518, 1459, 1341, 1256, 1203, 1032, 950, 899, 786, 659 cm^{-1}; MS (*m*/*z*): HRMS (ESI) Calcd. for $C_{29}H_{32}NO_6$($[M+H]^+$): 490.2224, found: 490.2238.

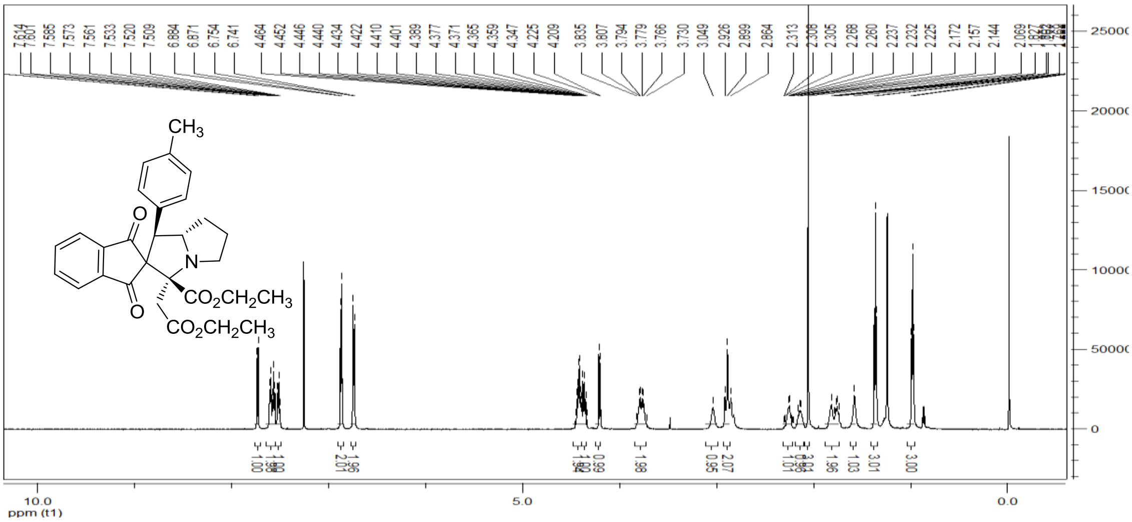

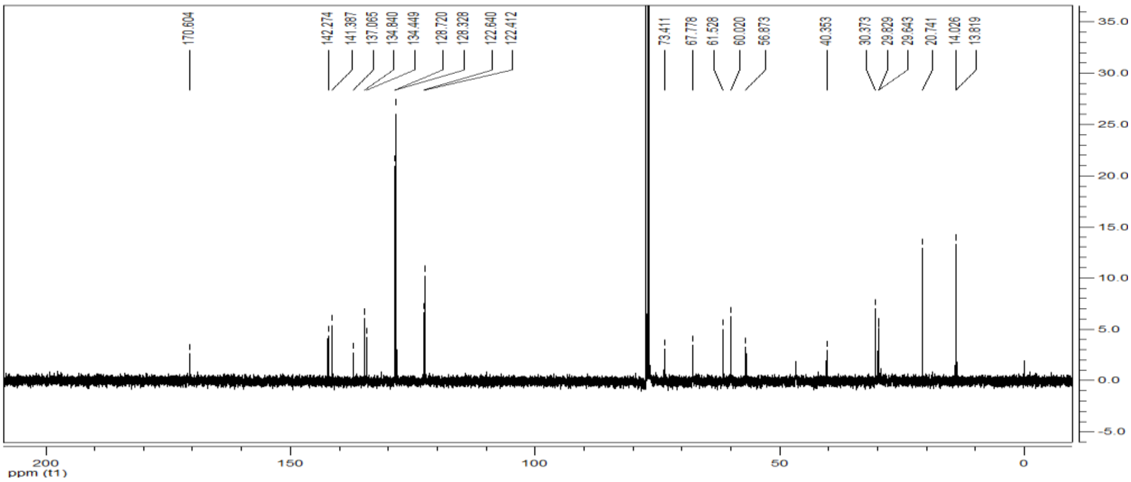

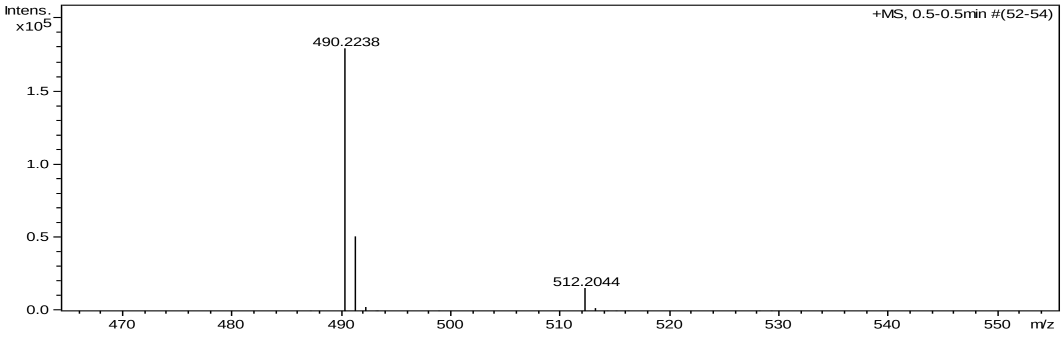

Methyl 5'-(2-methoxy-2-oxoethyl)-1,3-dioxo-7'-(p-tolyl)-1,3,3',5',7',7a'-hexahydro-1'H-spiro [indene-2,6'-pyrrolo[1,2-c]thiazole]-5'-carboxylate (1g): white solid, 68%, m.p. 142-144℃; ^{1}H NMR (400 MHz, $CDCl_3$) δ: 7.82 (d, J = 7.2 Hz, 1H, ArH), 7.76 (d, J = 7.2 Hz, 1H, ArH), 7.69-7.62 (m, 2H, ArH), 7.01 (d, J = 8.0 Hz, 2H, ArH), 6.87 (d, J = 8.0 Hz, 2H, ArH), 4.90-4.86 (m, 1H, CH), 4.55 (d, J = 12.0 Hz, 1H, CH), 4.22 (d, J = 12.0 Hz, 1H, CH), 3.65 (s, 3H, OCH_3), 3.60 (d, J = 8.8 Hz, 1H, CH), 3.57 (s, 1H, CH), 3.54 (s, 3H, OCH_3), 3.45 (d, J = 17.6 Hz, 1H, CH), 3.12-3.07 (m, 1H, CH), 2.72 (d, J = 11.6 Hz, 1H, CH), 2.13 (s, 3H, CH_3); ^{13}C NMR (150 MHz, $CDCl_3$) δ: 199.0, 198.8, 171.7, 171.1, 142.9, 141.4, 137.7, 135.8, 135.0, 129.9, 129.2, 129.0, 123.0, 122.7, 75.6, 70.0, 55.3, 54.3, 52.6, 51.9, 37.5, 35.3, 20.9; IR(KBr) υ: 3011, 2928, 2866, 1743, 1706, 1592, 1511, 1443, 1399, 1349, 1293, 1233, 1174, 1090, 992, 942, 898, 842, 786, 741 cm^{-1}; MS (m/z): HRMS (ESI) Calcd. for $C_{26}H_{25}NNaO_6S$ ($[M+Na]^+$): 502.1295, found: 502.1295.

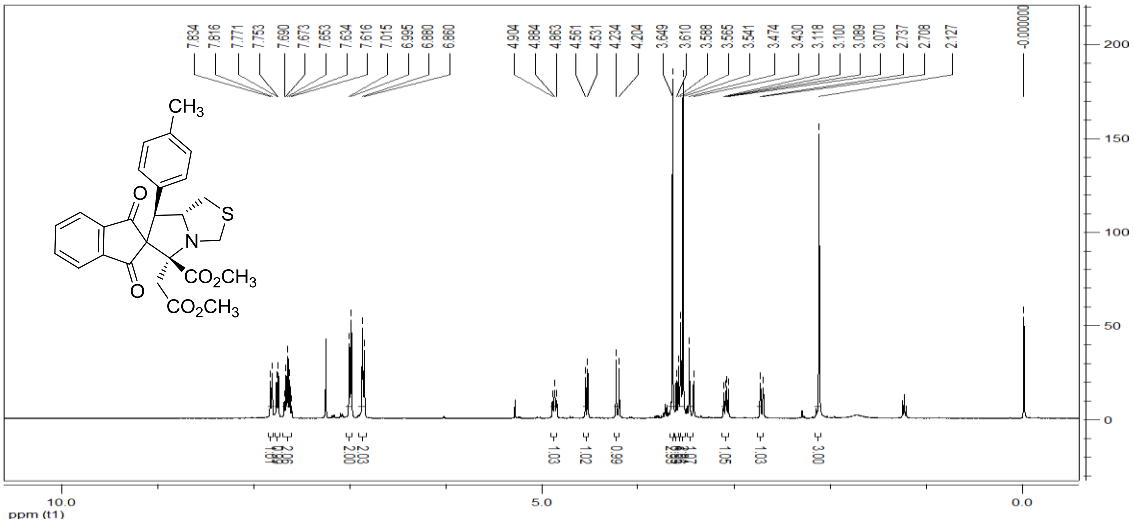

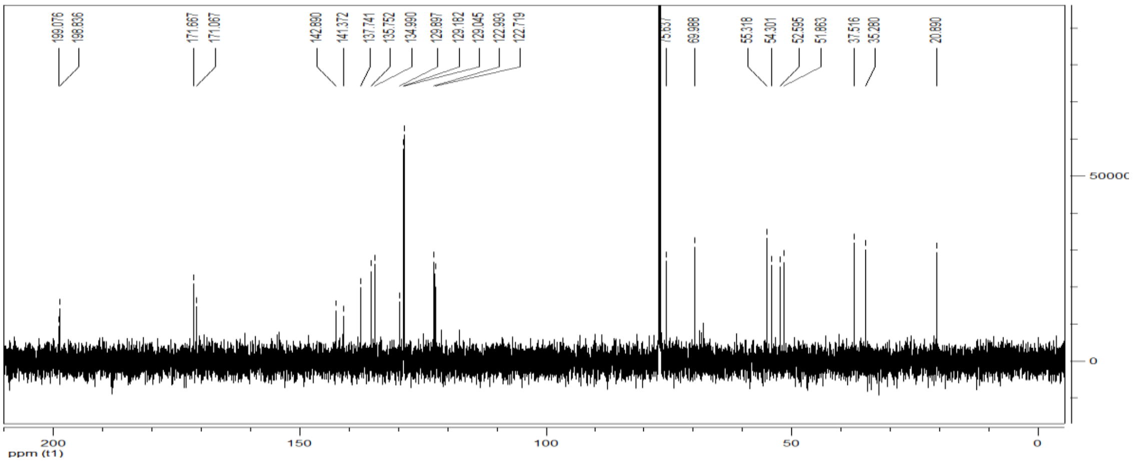

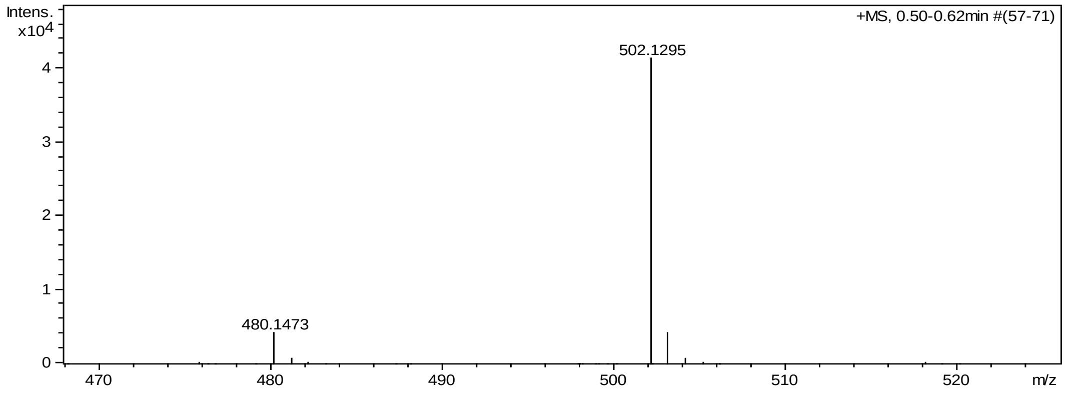

Methyl 7'-(4-bromophenyl)-5'-(2-methoxy-2-oxoethyl)-1,3-dioxo-1,3,3',5',7',7a'-hexahydro-1'H-spiro[indene-2,6'-pyrrolo[1,2-c]thiazole]-5'-carboxylate (1h): white solid, 77%, m.p. 118-120℃; ^{1}H NMR (600 MHz, $CDCl_3$) δ: 7.85 (d, J = 7.8 Hz, 1H, ArH), 7.77 (d, J = 7.2 Hz, 1H, ArH), 7.72 (t, J = 7.2 Hz, 1H, ArH), 7.69-7.67 (m, 1H, ArH), 7.22 (d, J = 9.0 Hz, 2H, ArH), 7.02 (d, J = 8.4 Hz, 2H, ArH), 4.88 (t, J = 8.4 Hz, 1H, CH), 4.54 (d, J = 12.0 Hz, 1H, CH), 4.20 (d, J = 12.6 Hz, 1H, CH), 3.65 (s, 3H, OCH_3), 3.58 (d, J = 3.6 Hz, 1H, CH), 3.56-3.55 (m, 4H, CH, OCH_3), 3.43 (d, J = 17.4 Hz, 1H, CH), 3.12-3.09 (m, 1H, CH), 2.70 (d, J = 12.0 Hz, 1H, CH); ^{13}C NMR (150 MHz, $CDCl_3$) δ: 198.8, 198.5, 171.6, 170.8, 142.9, 141.2, 136.1, 135.3, 132.2, 131.7, 131.0, 123.1, 122.8, 122.2, 75.8, 69.8, 68.0, 54.7, 54.2, 52.7, 51.9, 37.5, 35.2; IR(KBr) υ: 3007, 2931, 2865, 1744, 1706, 1592, 1488, 1442, 1403, 1353, 1353, 1235, 1174, 1085, 1004, 902, 826, 789, 746, 690 cm^{-1}; MS (m/z): HRMS (ESI) Calcd. for $C_{25}H_{22}BrNNaO_6S$($[M+Na]^+$): 566.0243, found: 566.0234.

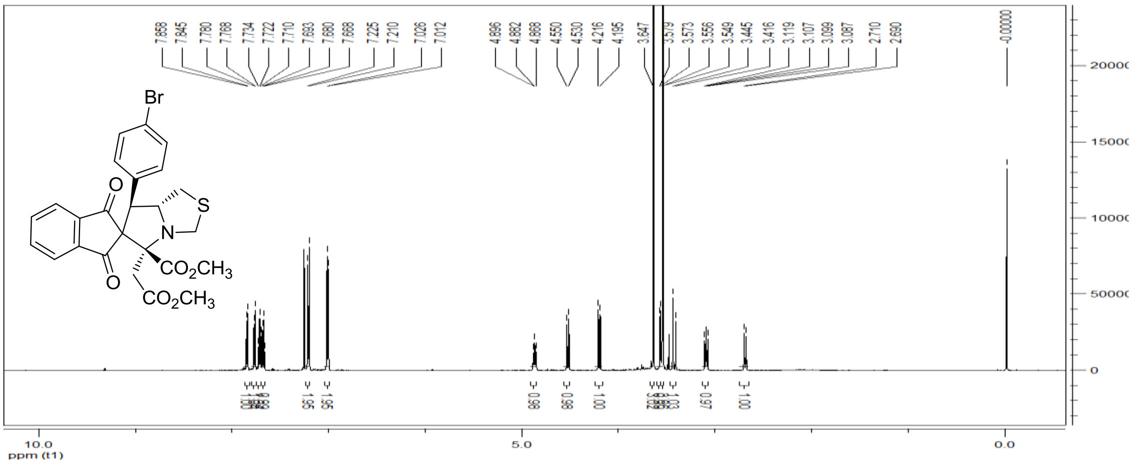

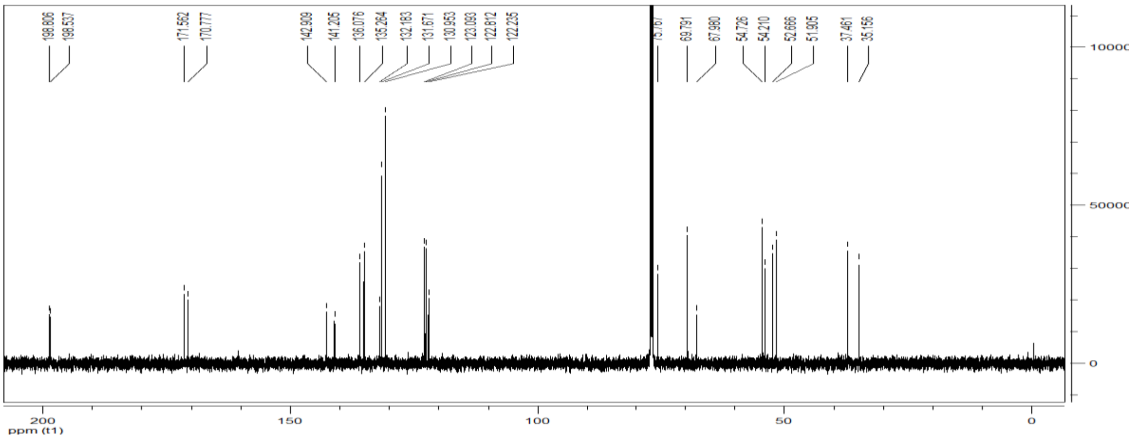

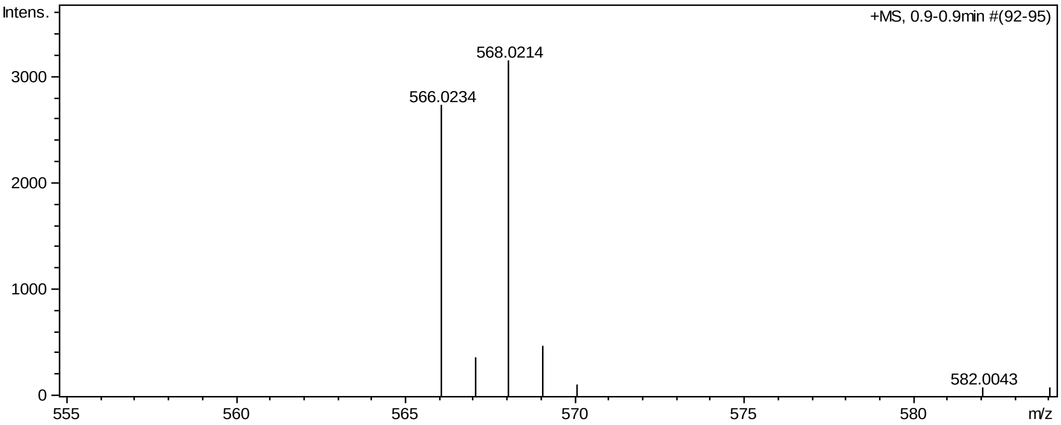

Ethyl 7'-(4-chlorophenyl)-5'-(2-ethoxy-2-oxoethyl)-1,3-dioxo-1,3,3',5',7',7a'-hexahydro-1'H-spiro[indene-2,6'-pyrrolo[1,2-c]thiazole]-5'-carboxylate (1i): white solid, 74%, m.p. 98-100℃; ^{1}H NMR (600 MHz, $CDCl_3$) δ: 7.86 (d, *J* = 7.2 Hz, 1H, ArH), 7.77 (d, *J* = 7.2 Hz, 1H, ArH), 7.72 (t, *J* = 7.2 Hz, 1H, ArH), 7.69-7.67 (m, 1H, ArH), 7.12-7.09 (m, 2H, ArH), 7.06 (d, *J* = 8.4 Hz, 2H, ArH), 4.92-4.89 (m, 1H, CH), 4.59 (d, *J* = 12.6 Hz, 1H, CH), 4.22 (d, *J* = 12.6 Hz, 1H, CH), 4.15-4.09 (m, 1H, CH), 4.08-4.02 (m, 1H, CH), 3.99 (q, *J* = 7.2 Hz, 2H, CH_2), 3.61-3.54 (m, 1H, CH), 3.42 (d, *J* = 17.4 Hz, 1H, CH), 3.13-3.10 (m, 1H, CH), 2.71 (d, *J* = 12.0 Hz, 1H, CH), 1.26-1.24 (m, 4H, CH, CH_3), 1.00 (t, *J* = 7.2 Hz, 3H, CH_3); ^{13}C NMR (150 MHz, $CDCl_3$) δ: 198.8, 198.5, 171.0, 170.1, 143.0, 141.3, 135.9, 135.2, 133.9, 131.7, 130.7, 128.7, 123.0, 122.8, 75.5, 69.8, 67.9, 61.8, 60.9, 54.7, 54.2, 37.6, 35.4, 14.1, 13.5; IR(KBr) υ: 2983, 2928, 2859, 1736, 1708, 1594, 1491, 1463, 1399, 1372, 1342, 1266, 1222, 1184, 1092, 1021, 909, 833, 786, 742 cm^{-1}; MS (*m/z*): HRMS (ESI) Calcd. for $C_{27}H_{27}ClNO_6S$($[M+H]^+$): 528.1242, found: 528.1246.

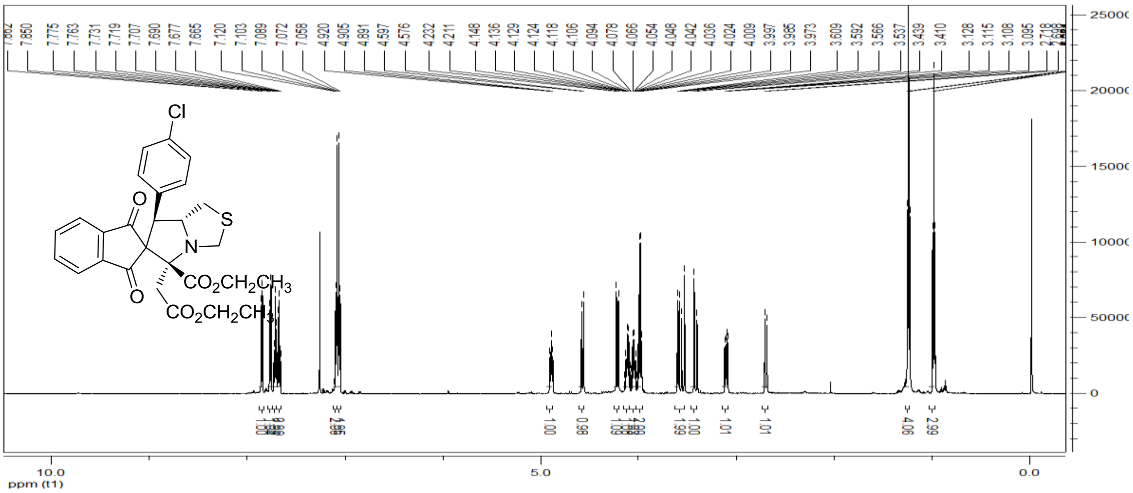

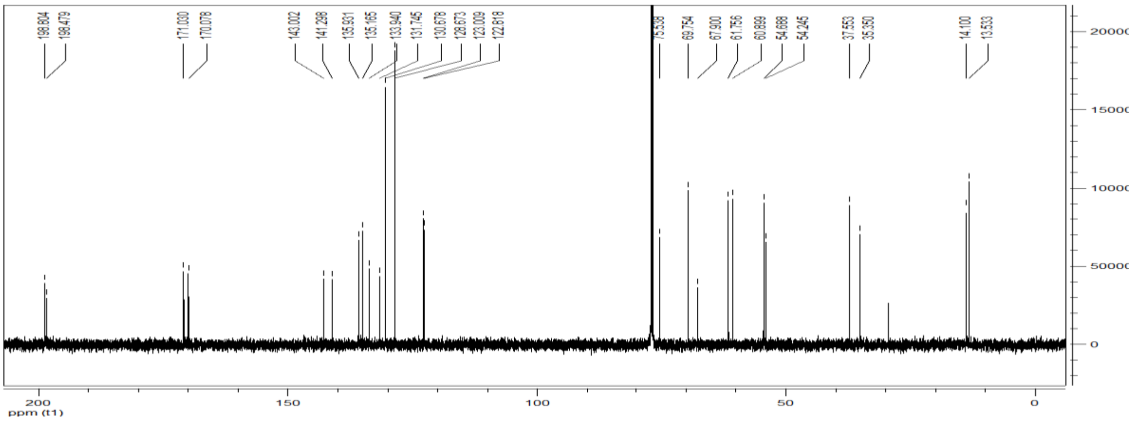

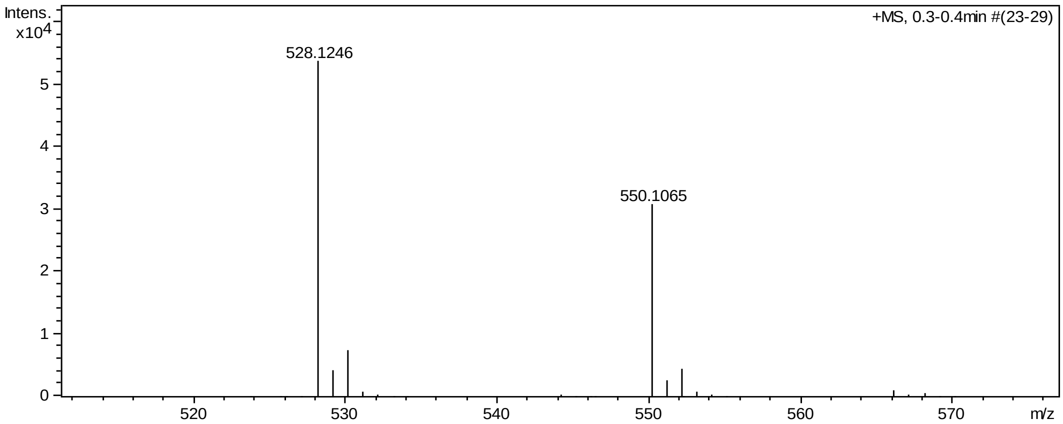

Methyl 4'-(4-chlorophenyl)-2'-(2-methoxy-2-oxoethyl)-1'-methyl-1,3-dioxo-1,3-dihydrospiro-[indene-2,3'-pyrrolidine]-2'-carboxylate (2a): white solid, 80%, m.p. 138-140℃; ^{1}H NMR (400 MHz, $CDCl_3$) δ: 7.82 (d, *J* = 7.6Hz, 1H, ArH), 7.65-7.61 (m, 1H, ArH), 7.56-7.53 (m, 1H, ArH), 7.48 (d, *J* = 7.6Hz, 1H, ArH), 6.94-6.92 (m, 2H, ArH), 6.91-6.89 (m, 2H, ArH), 4.39-4.35 (m, 1H, CH), 3.95 (s, 3H, OCH_3), 3.79-3.75 (m, 1H, CH), 3.52-3.47 (m, 1H, CH), 3.36 (s, 3H, OCH_3), 2.99 (d, *J* = 16.4Hz, 1H, CH), 2.91 (d, *J* = 16.4Hz, 1H, CH), 2.51 (s, 3H, CH_3); ^{13}C NMR (100 MHz, $CDCl_3$) δ: 198.6, 198.3, 171.4, 171.1, 142.1, 141.3, 135.0, 134.8, 133.9, 133.2, 130.2, 128.1, 122.7, 122.3, 74.0, 68.9, 58.4, 52.3, 51.7, 51.4, 37.3, 35.0; IR(KBr) υ: 3013, 2952, 2861, 2801, 1733, 1596, 1488, 1439, 1360, 1258, 1216, 1134, 1082, 1043, 1004, 891, 833, 769, 705 cm^{-1}; MS (*m*/*z*): HRMS (ESI) Calcd. for $C_{24}H_{23}ClNO_6$ ($[M+H]^+$): 456.1208, found: 456.1233.

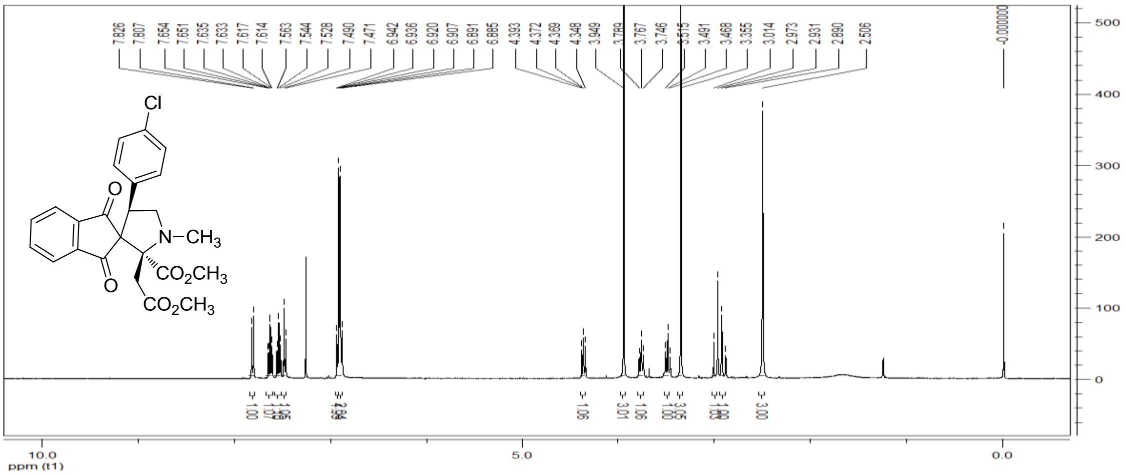

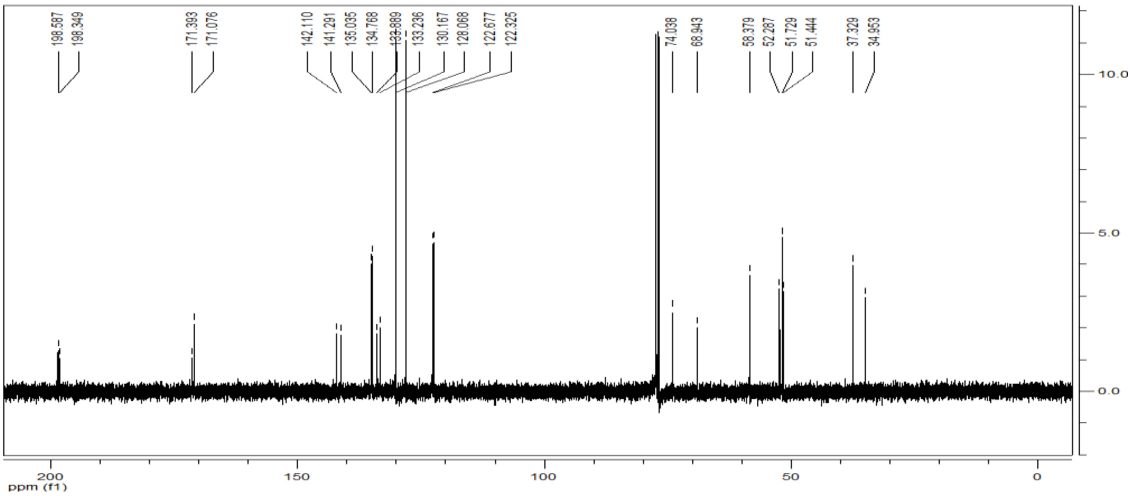

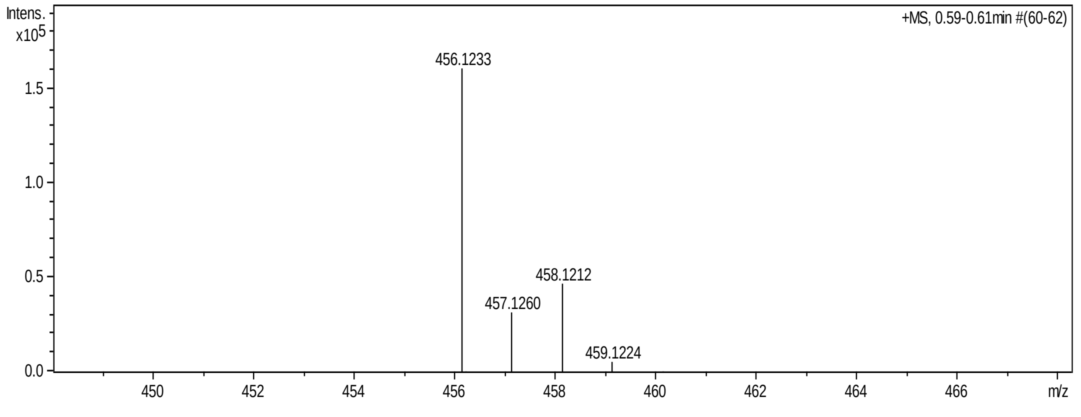

Methyl 4'-(4-bromophenyl)-2'-(2-methoxy-2-oxoethyl)-1'-methyl-1,3-dioxo-1,3-dihydrospiro-[indene-2,3'-pyrrolidine]-2'-carboxylate (2b): white solid, 79%, m.p. 136-138℃; ^{1}H NMR (600 MHz, $CDCl_3$) δ: 7.82 (d, *J* = 7.8 Hz, 1H, ArH), 7.65-7.63 (m, 1H, ArH), 7.57-7.54 (m, 1H, ArH), 7.49 (d, *J* = 7.2 Hz, 1H, ArH), 7.08 (d, *J* = 7.8 Hz, 2H, ArH), 6.84 (d, *J* = 7.8 Hz, 2H, ArH), 4.36 (t, *J* = 9.0 Hz, 1H, CH), 3.95 (s, 3H, OCH_3), 3.76 (brs, 1H, CH), 3.51-3.48 (m, 1H, CH), 3.36 (s, 3H, OCH_3), 3.01-2.90 (m, 2H, CH_2), 2.51 (s, 3H, CH_3); ^{13}C NMR (150 MHz, $CDCl_3$) δ: 198.6, 198.3, 171.4, 171.1, 142.2, 141.4, 135.0, 134.8, 134.5, 131.0, 130.6, 122.7, 122.4, 121.4, 74.1, 69.0, 58.4, 52.2, 51.8, 51.4, 37.4, 34.9; IR (KBr) υ: 3011, 2952, 2904, 2861, 2802, 1733, 1594, 1484, 1443, 1360, 1258, 1217, 1131, 1076, 1043, 1005, 976, 945, 891, 841, 769, 702 cm^{-1}; MS (*m*/*z*): HRMS (ESI) Calcd. for $C_{24}H_{23}BrNO_6$ ($[M+H]^+$): 500.0703, found: 500.0706.

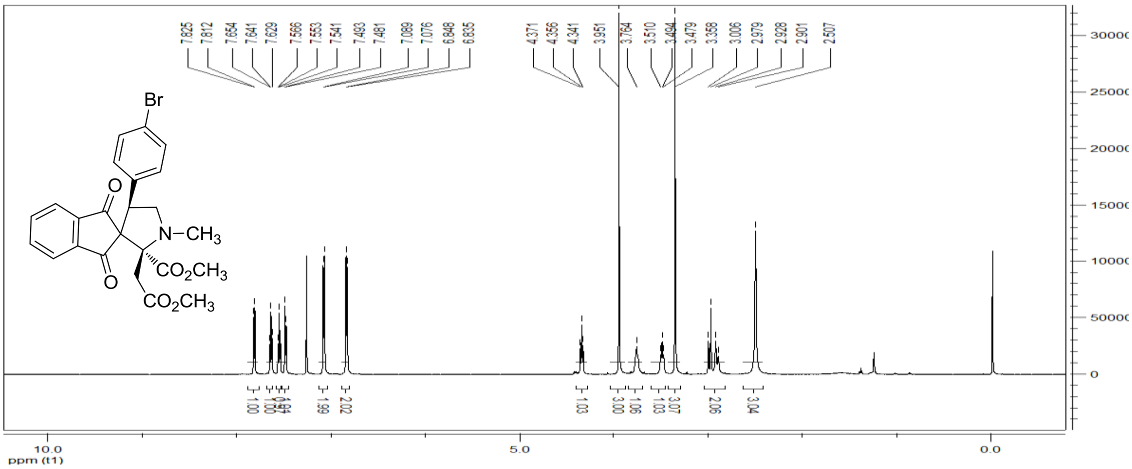

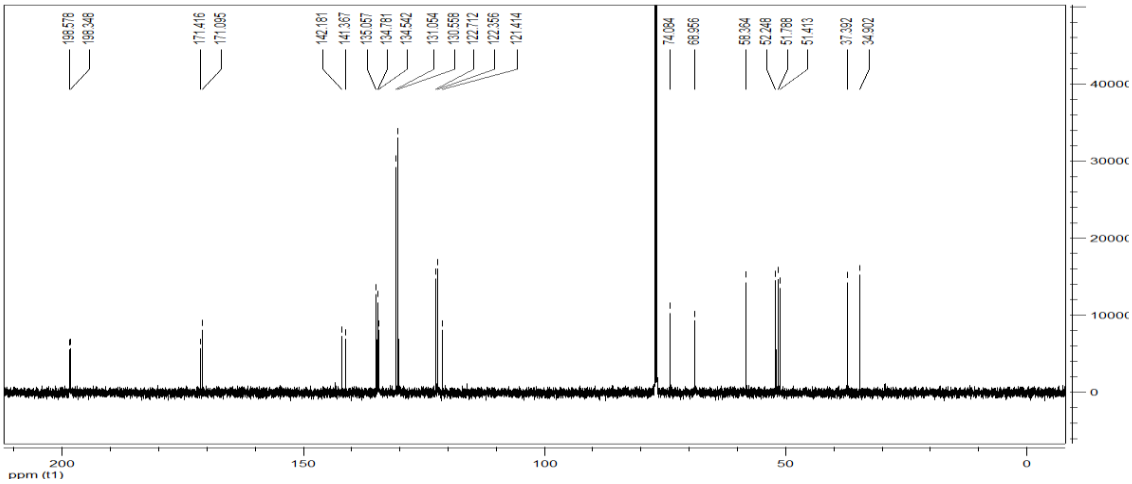

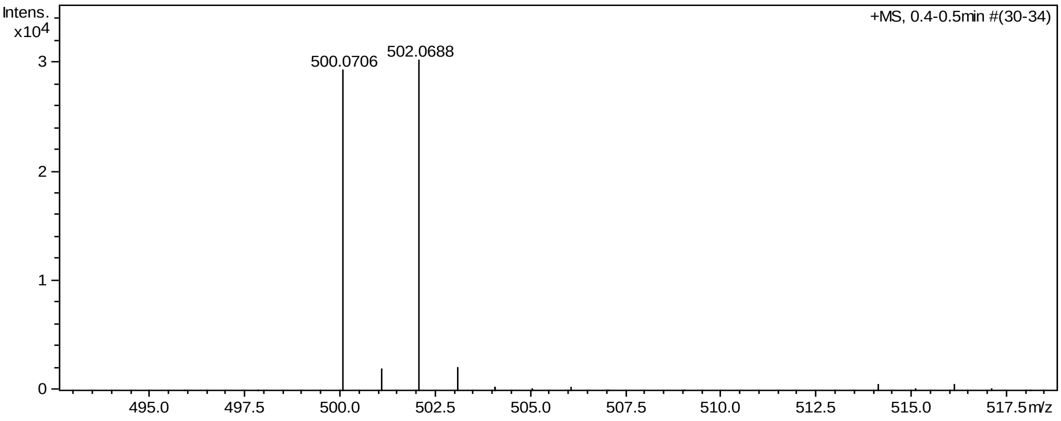

Methyl 2'-(2-methoxy-2-oxoethyl)-4'-(3-methoxyphenyl)-1'-methyl-1,3-dioxo-1,3-dihydro-spiro[indene-2,3'-pyrrolidine]-2'-carboxylate (2c): white solid, 72%, m.p. 156-158℃; ^{1}H NMR (600 MHz, $CDCl_3$) δ: 7.80 (d, *J* = 7.8 Hz, 1H, ArH), 7.59 (t, *J* = 7.2 Hz, 1H, ArH), 7.50 (t, *J* = 7.2 Hz, 1H, ArH), 7.46 (d, *J* = 7.2 Hz, 1H, ArH), 6.84 (t, *J* = 7.8 Hz, 1H, ArH), 6.54 (d, *J* = 7.2 Hz, 1H, ArH), 6.49-6.47 (m, 2H, ArH), 4.38 (t, *J* = 9.0 Hz, 1H, CH), 3.95 (s, 3H, OCH_3), 3.83-3.80 (m, 1H, CH), 3.60 (s, 3H, OCH_3), 3.49-3.46 (m, 1H, CH), 3.34 (s, 3H, OCH_3), 3.00-2.92 (m, 2H, CH_2), 2.50 (s, 3H, CH_3); ^{13}C NMR (100 MHz, $CDCl_3$) δ: 198.7, 198.5, 171.5, 171.1, 159.0, 142.3, 141.5, 136.8, 134.6, 134.4, 128.9, 122.6, 122.2, 121.3, 113.8, 113.7, 74.0, 69.1, 58.2, 55.1, 52.6, 52.2, 51.3, 37.3, 34.9; IR(KBr) υ: 3084, 2995, 2948, 2908, 2847, 2803, 1734, 1590, 1487, 1443, 1367, 1329, 1259, 1215, 1157, 1059, 1004, 977, 944, 882, 785, 758, 696 cm^{-1}; MS (*m*/*z*): HRMS (ESI) Calcd. for $C_{25}H_{26}NO_7$ ($[M+H]^+$): 452.1704, found: 452.1709.

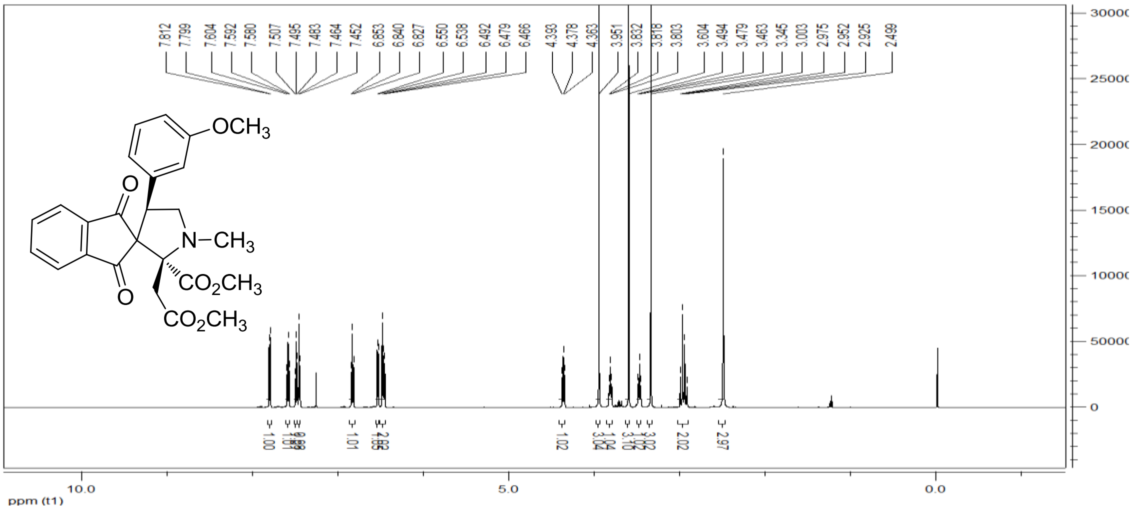

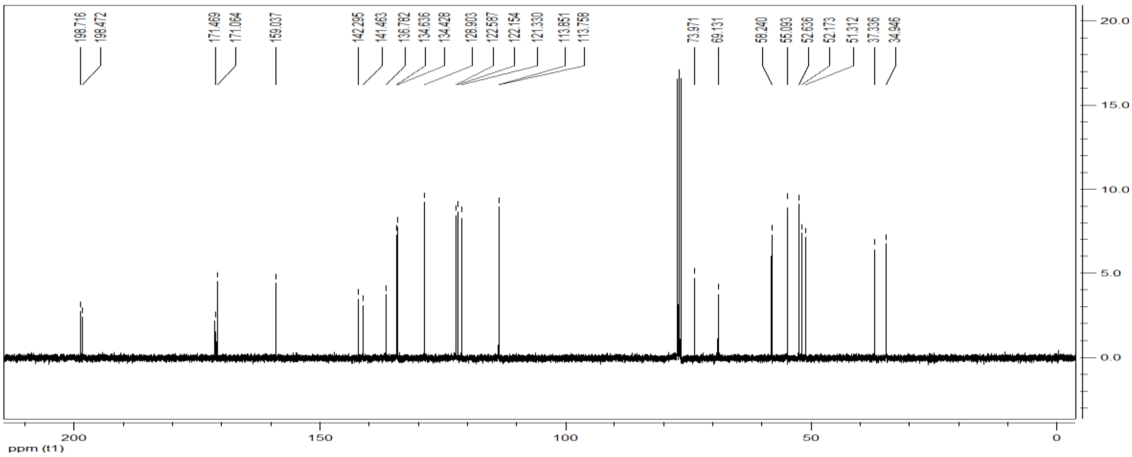

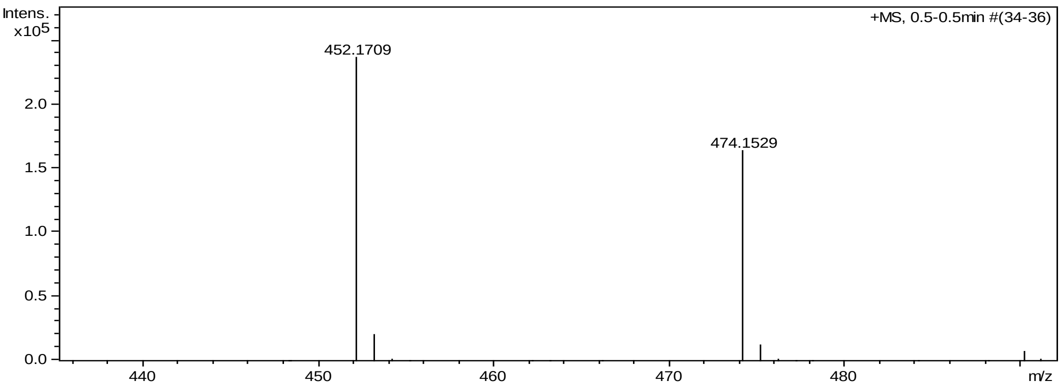

Methyl 2'-(2-methoxy-2-oxoethyl)-4'-(2-methoxyphenyl)-1'-methyl-1,3-dioxo-1,3-dihydro-spiro[indene-2,3'-pyrrolidine]-2'-carboxylate (2d): white solid, 69%, m.p. 150-152℃; ^{1}H NMR (400 MHz, $CDCl_3$) δ: 7.84 (d, *J* = 7.6Hz, 1H, ArH), 7.63-7.60 (m, 1H, ArH), 7.46-7.42 (m, 1H, ArH), 7.31-7.29 (m, 2H, ArH), 6.95-6.91 (m, 1H, ArH), 6.83-6.80 (m, 1H, ArH), 6.19 (d, *J* = 8.0Hz, 1H, ArH), 4.84-4.80 (m, 1H, CH), 3.96 (s, 3H, OCH_3), 3.79-3.73 (m, 1H, CH), 3.54-3.49 (m, 1H, CH), 3.34 (s, 3H, OCH_3), 3.30 (s, 3H, OCH_3), 3.03 (brs, 2H, CH_2), 2.50 (s, 3H, CH_3); ^{13}C NMR (100 MHz, $CDCl_3$) δ: 198.6, 197.2, 171.7, 171.1, 156.8, 142.3, 140.5, 134.0, 133.9, 129.3, 128.2, 124.1, 122.0, 121.6, 120.1, 108.6, 74.0, 68.4, 58.3, 54.1, 52.1, 51.3, 43.6, 37.2, 34.9; IR(KBr) υ: 3063, 2947, 2878, 2804, 1720, 1595, 1442, 1355, 1257, 1213, 1131, 1060, 1013, 936, 888, 841, 753 cm^{-1}; MS (*m/z*): HRMS (ESI) Calcd. for $C_{25}H_{26}NO_7$ ($[M+H]^+$): 452.1704, found: 452.1726.

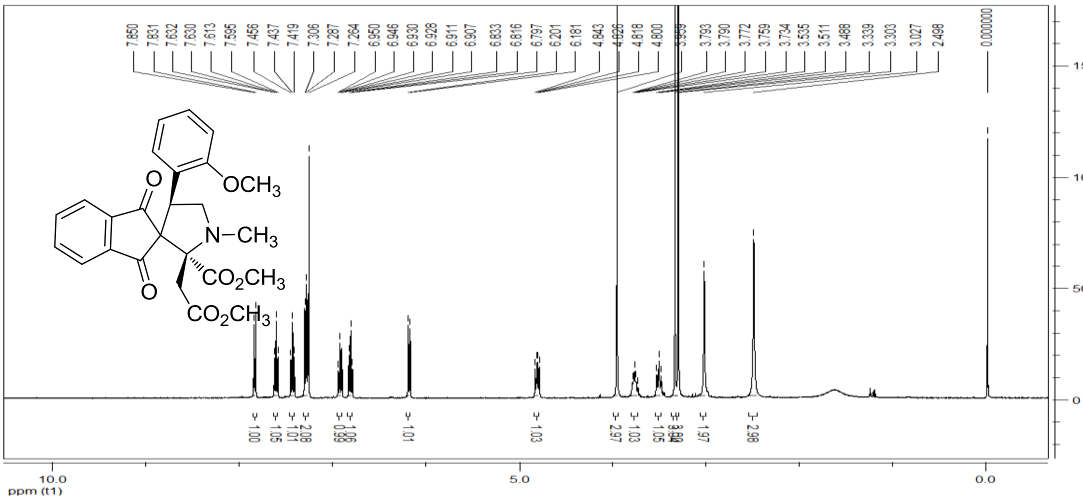

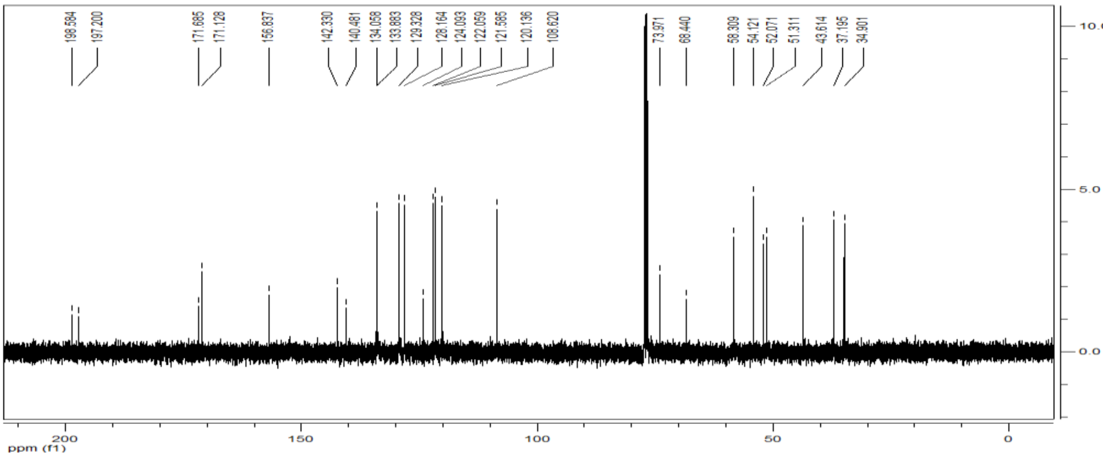

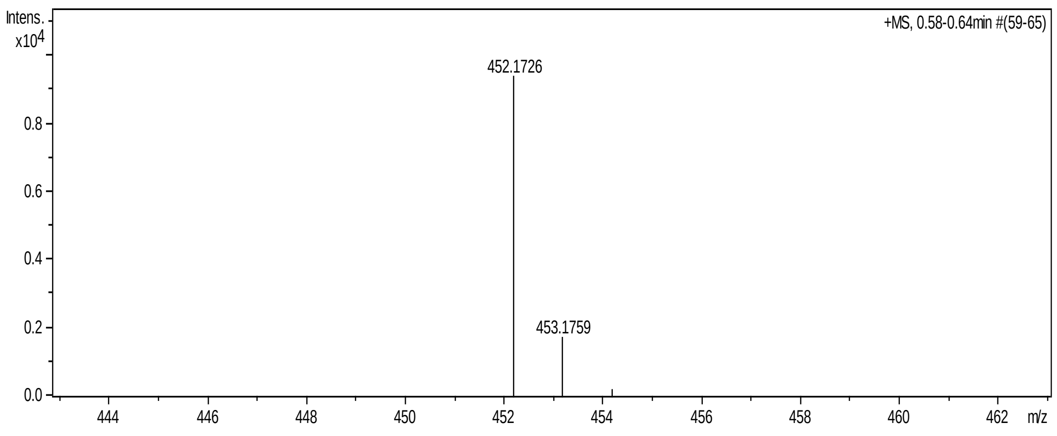

Ethyl 4'-(4-bromophenyl)-2'-(2-ethoxy-2-oxoethyl)-1'-methyl-1,3-dioxo-1,3-dihydrospiro-[indene-2,3'-pyrrolidine]-2'-carboxylate (2e): white solid, 75%, m.p. 146-148℃; ^{1}H NMR (400 MHz, $CDCl_3$) δ: 7.81 (d, J = 7.6Hz, 1H, ArH), 7.65-7.61 (m, 1H, ArH), 7.56-7.52 (m, 1H, ArH), 7.48 (d, J = 7.6Hz, 1H, ArH), 7.08 (d, J = 8.4Hz, 2H, ArH), 6.84 (d, J = 8.4Hz, 2H, ArH), 4.46-4.41 (m, 2H, CH_2), 4.38 (d, J = 9.2Hz, 1H, CH), 3.87-3.81 (m, 1H, CH), 3.79-3.72 (m, 2H, CH_2), 3.50 (t, J = 9.6Hz, 1H, CH), 2.97 (d, J = 16.4Hz, 1H, CH), 2.87 (d, J = 16.0Hz, 1H, CH), 2.50 (s, 3H, CH_3), 1.40 (t, J = 7.2Hz, 3H, CH_3), 1.02 (t, J = 7.2Hz, 3H, CH_3); ^{13}C NMR (100 MHz, $CDCl_3$) δ: 198.7, 198.2, 170.7, 170.5, 142.2, 141.3, 135.0, 134.7, 134.6, 131.0, 130.5, 122.6, 122.3, 121.3, 73.9, 68.9, 61.5, 60.3, 58.4, 51.8, 37.6, 34.9, 14.2, 13.8; IR(KBr) υ: 3043, 2987, 2947, 2864, 1729, 1592, 1486, 1454, 1409, 1379, 1338, 1259, 1216, 1137, 1070, 1023, 970, 870, 827, 773, 732, 702 cm^{-1}; MS (m/z): HRMS (ESI) Calcd. for $C_{26}H_{27}BrNO_6$ ([M+H]$^+$): 528.1022, found: 528.1027.

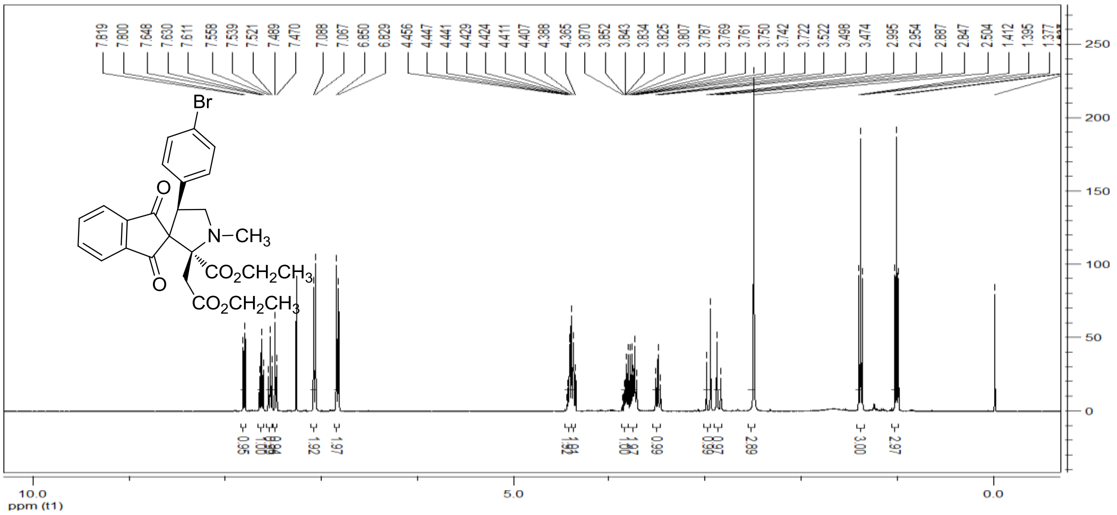

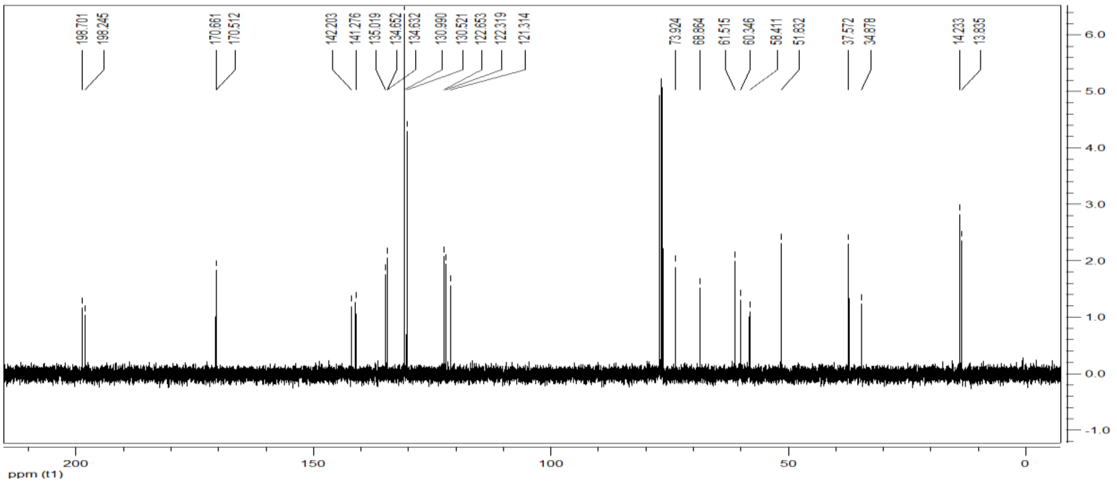

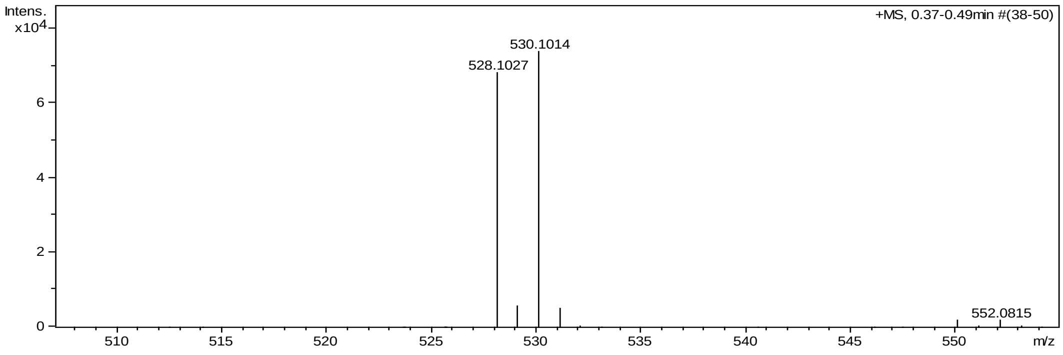

Dimethyl 2-(4'-(4-bromophenyl)-2'-(2-methoxy-2-oxoethyl)-2'-(methoxycarbonyl)-1,3-dioxo-1,3-dihydrospiro[indene-2,3'-pyrrolidin]-1'-yl)maleate (3a): white solid, 78%, m.p. 176-178℃; ^{1}H NMR (600 MHz, $CDCl_3$) δ: 7.76 (d, J = 7.2 Hz, 1H, ArH), 7.64-7.61 (m, 1H, ArH), 7.58-7.55 (m, 1H, ArH), 7.52 (d, J = 7.8 Hz, 1H, ArH), 7.08 (d, J = 8.4 Hz, 2H, ArH), 6.79 (d, J = 9.0 Hz, 2H, ArH), 4.63 (s, 1H, CH), 4.35-4.32 (m, 1H, CH), 4.28-4.24 (m, 1H, CH), 4.02 (s, 3H, OCH_3), 3.94 (s, 3H, OCH_3), 3.85-3.82 (m, 1H, CH), 3.66 (s, 3H, OCH_3), 3.55 (d, J = 16.8 Hz, 1H, CH), 3.37 (s, 3H, OCH_3), 3.06 (d, J = 17.4 Hz, 1H, CH); ^{13}C NMR (100 MHz, $CDCl_3$) δ: 197.8, 195.9, 170.4, 167.2, 165.3, 149.0, 141.9, 140.4, 135.4, 135.0, 131.4, 130.7, 129.8, 122.7, 122.6, 122.4, 89.7, 71.2, 68.4, 53.6, 53.1, 51.9, 51.8, 51.0, 49.8, 33.7; IR(KBr) υ: 3096, 3004, 2955, 2892, 2849, 1743, 1708, 1598, 1480, 1433, 1391, 1214, 1166, 1119, 1047, 1006, 961, 884, 844, 813, 774, 710 cm^{-1}; MS (m/z): HRMS (ESI) Calcd. for $C_{29}H_{26}BrNNaO_{10}$($[M+Na]^+$): 650.0632, found: 650.0630.

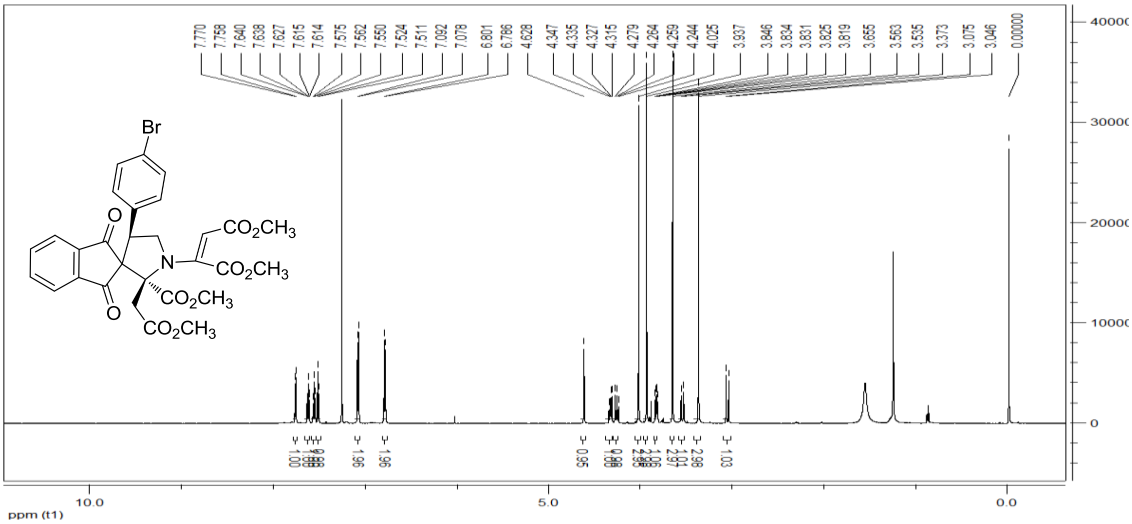

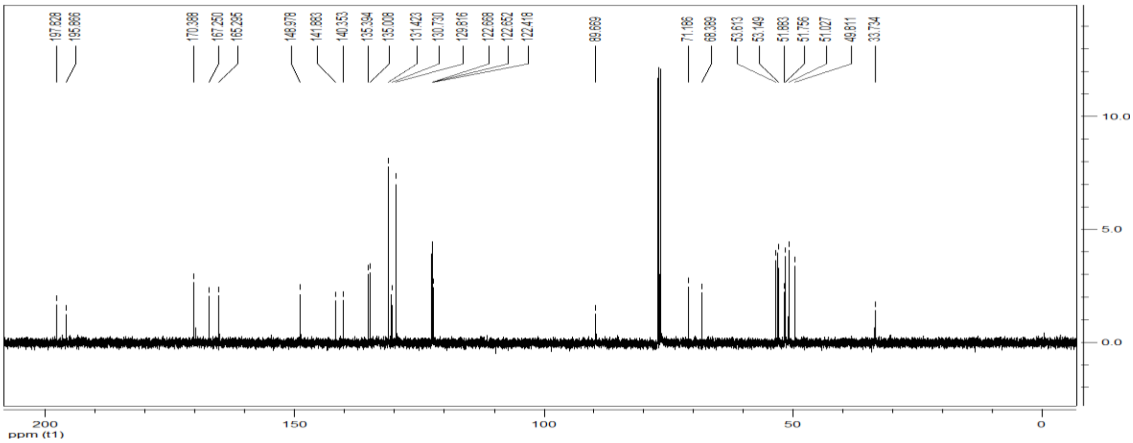

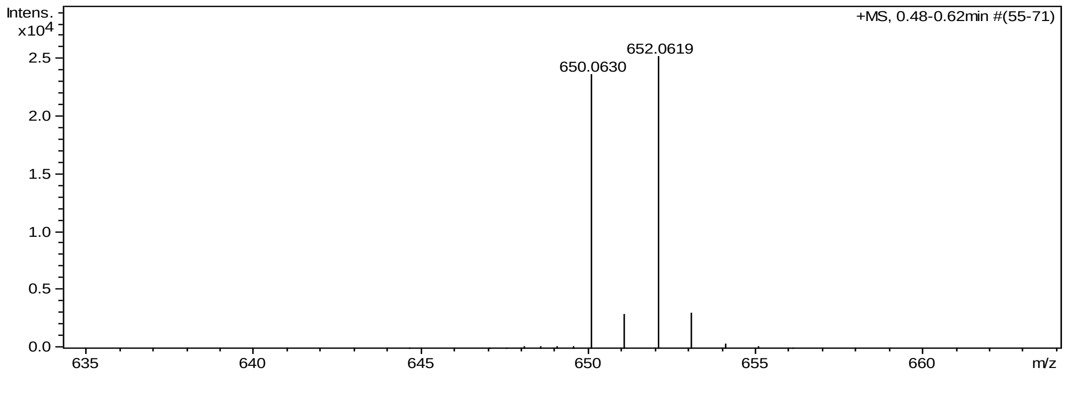

Diethyl 2-(4'-(4-bromophenyl)-2'-(2-ethoxy-2-oxoethyl)-2'-(ethoxycarbonyl)-1,3-dioxo-1,3-dihydrospiro[indene-2,3'-pyrrolidin]-1'-yl)maleate (3b): white solid, 72%, m.p. 194-196℃; ^{1}H NMR (600 MHz, $CDCl_3$) δ: 7.75 (d, *J* = 7.2 Hz, 1H, ArH), 7.63-7.60 (m, 1H, ArH), 7.56-7.53 (m, 1H, ArH), 7.52 (d, *J* = 7.2 Hz, 1H, ArH), 7.09-7.07 (m, 2H, ArH), 6.79 (d, *J* = 8.4 Hz, 2H, ArH), 4.63 (s, 1H, CH), 4.57-4.51 (m, 1H, CH), 4.49-4.45 (m, 1H, CH), 4.44-4.41 (m, 1H, CH), 4.39-4.35 (m, 2H, CH_2), 4.27-4.23 (m, 1H, CH), 4.11 (q, *J* = 7.2 Hz, 2H, CH_2), 3.86-3.83 (m, 1H, CH), 3.82-3.75 (m, 2H, CH_2), 3.58 (d, *J* = 17.4 Hz, 1H, CH), 3.04 (d, *J* = 17.4 Hz, 1H, CH), 1.44 (t, *J* = 7.2 Hz, 3H, CH_3), 1.39 (t, *J* = 7.2 Hz, 3H, CH_3), 1.25 (t, *J* = 7.2 Hz, 3H, CH_3), 1.04 (t, *J* = 7.2 Hz, 3H, CH_3); ^{13}C NMR (150 MHz, $CDCl_3$) δ: 198.0, 195.9, 170.1, 169.3, 166.8, 165.0, 149.2, 142.0, 140.4, 135.4, 134.9, 131.4, 131.0, 129.8, 122.7, 122.6, 122.4, 90.1, 71.2, 68.4, 63.0, 62.4, 60.8, 59.7, 51.8, 49.8, 34.1, 14.4, 14.1, 13.8; IR(KBr) υ: 3089, 2984, 2902, 1742, 1703, 1582, 1466, 1405, 1387, 1347, 1210, 1154, 1116, 1019, 954, 862, 811, 769, 701 cm^{-1}; MS (*m*/*z*): HRMS (ESI) Calcd. for $C_{33}H_{34}BrNNaO_{10}$($[M+Na]^+$): 706.1258, found: 706.1267.

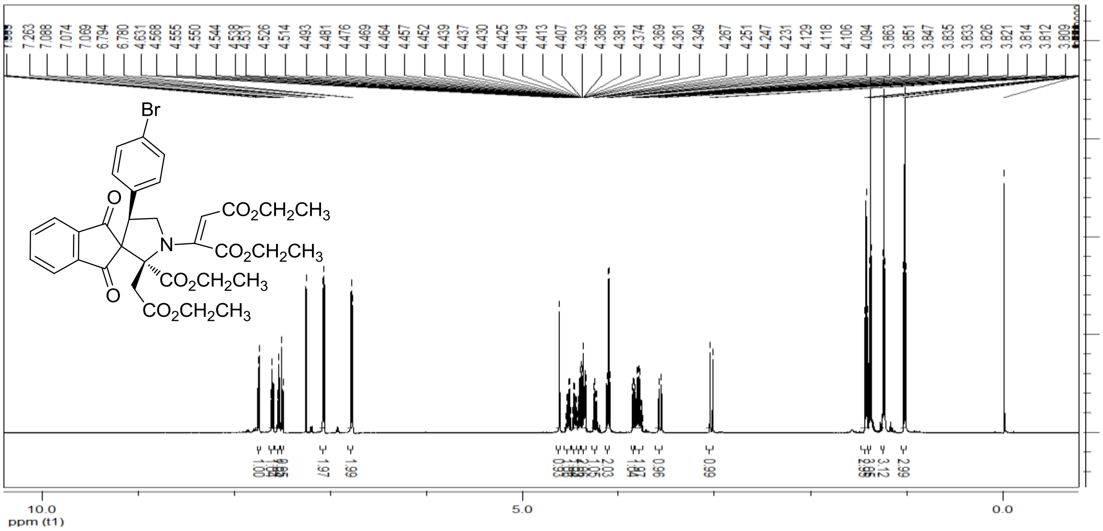

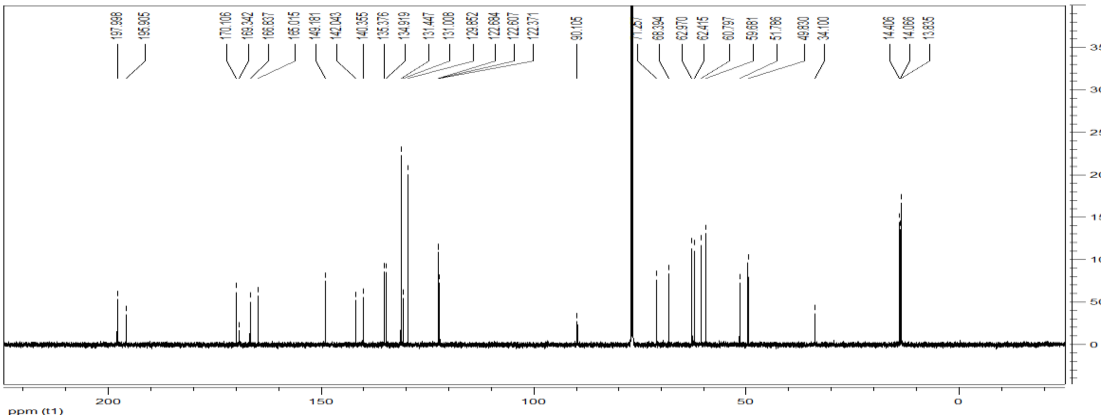

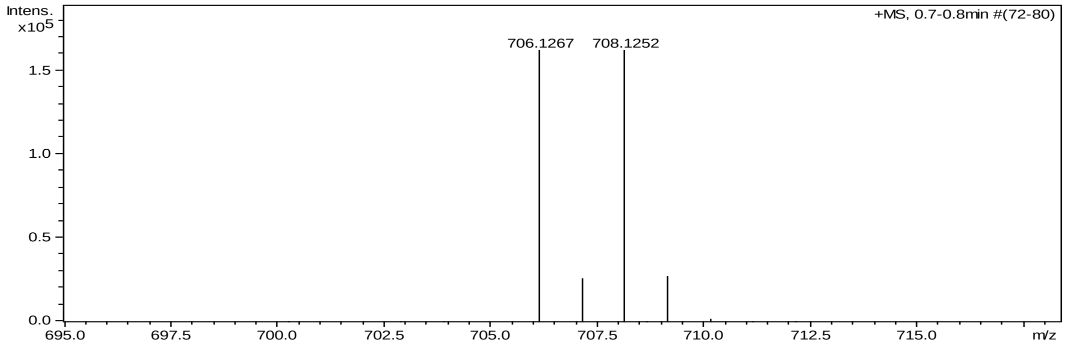

Diethyl 2-(2'-(2-ethoxy-2-oxoethyl)-2'-(ethoxycarbonyl)-4'-(3-methoxyphenyl)-1,3-dioxo-1,3-dihydrospiro[indene-2,3'-pyrrolidin]-1'-yl)maleate (3c): white solid, 68%, m.p. 180-182℃; ^{1}H NMR (400 MHz, $CDCl_3$) δ: 7.74 (d, J = 10.8 Hz, 1H, ArH), 7.57-7.56 (m, 1H, ArH), 7.55-7.50 (m, 2H, ArH), 6.85 (t, J = 11.4 Hz, 1H, ArH), 6.54-6.47 (m, 2H, ArH), 6.41 (s, 1H, ArH), 4.63 (s, 1H, CH), 4.57-4.53 (m, 1H, CH), 4.51-4.42 (m, 2H, CH_2), 4.40-4.36 (m, 2H, CH_2), 4.31-4.26 (m, 1H, CH), 4.14-4.09 (m, 2H, CH_2), 3.88-3.84 (m, 1H, CH), 3.83-3.78 (m, 2H, CH_2), 3.61-3.56 (m, 4H, CH, OCH_3), 3.08 (d, J = 17.2 Hz, 1H, CH), 1.45-1.38 (m, 5H, CH_2, CH_3), 1.27-1.19 (m, 4H, CH, CH_3), 1.05-1.01 (m, 3H, CH_3); ^{13}C NMR (100 MHz, $CDCl_3$) δ: 198.0, 196.0, 170.1, 169.4, 166.8, 165.0, 159.2, 149.3, 142.2, 140.5, 134.9, 134.5, 133.3, 129.3, 122.5, 122.4, 120.6, 114.1, 113.7, 89.8, 71.2, 68.6, 62.8, 62.3, 60.7, 59.6, 55.1, 51.8, 50.5, 34.1, 14.4, 14.0, 13.8; IR(KBr) υ: 3098, 2984, 2905, 2842, 1741, 1701, 1575, 1465, 1388, 1346, 1208, 1156, 1117, 1024, 869, 792, 692 cm^{-1}; MS (m/z): HRMS (ESI) Calcd. for $C_{34}H_{37}NNaO_{11}$ ($[M+Na]^+$): 658.2259, found: 658.2278.

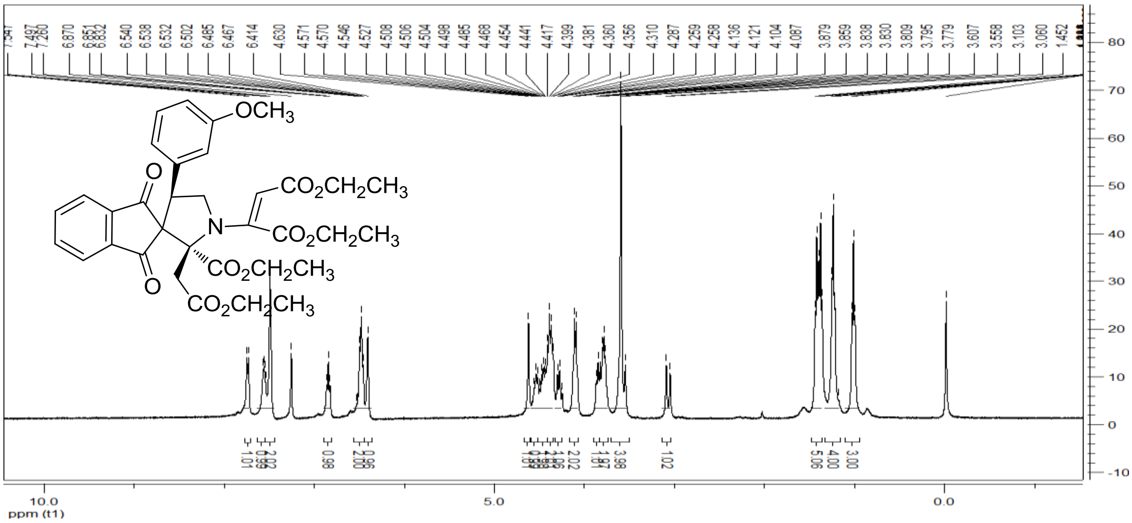

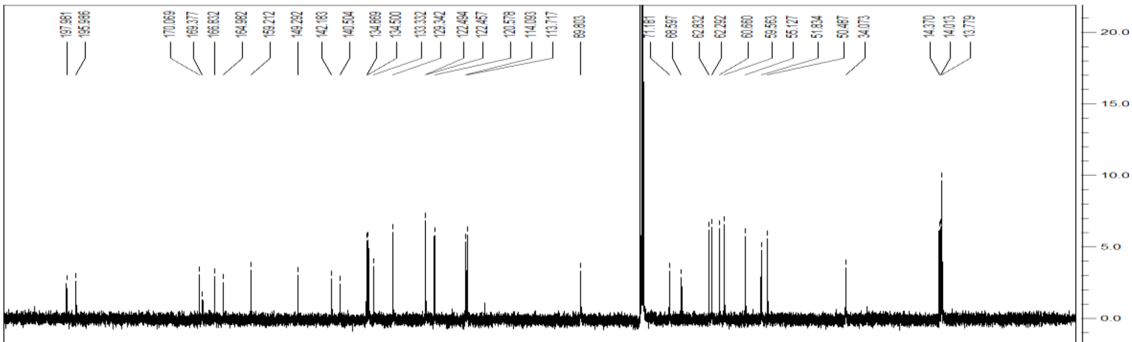

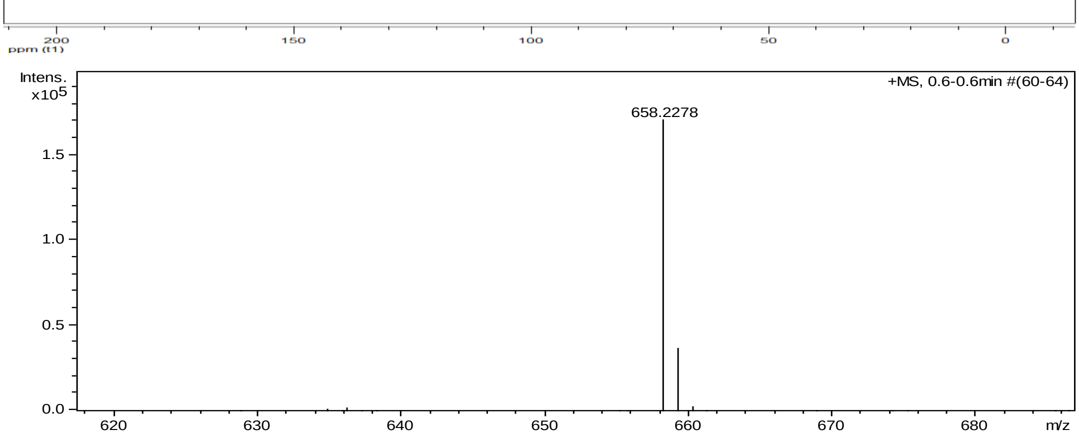

Dimethyl 2-(2'-(2-methoxy-2-oxoethyl)-2'-(methoxycarbonyl)-5'-methyl-1,3-dioxo-4'-(p-tolyl)-1,3-dihydrospiro[indene-2,3'-pyrrolidin]-1'-yl)maleate (3d): white solid, 69%, m.p. 188-190℃; ^{1}H NMR (400 MHz, $CDCl_3$) δ: 7.89 (d, *J* = 7.6 Hz, 1H, ArH), 7.82 (d, *J* = 7.2 Hz, 1H, ArH), 7.77-7.69 (m, 2H, ArH), 6.96 (d, *J* = 8.0 Hz, 2H, ArH), 6.88 (d, *J* = 8.0 Hz, 2H, ArH), 5.00 (s, 1H, CH), 4.91 (s, 1H, CH), 4.48 (q, *J* = 6.4 Hz, 1H, CH), 3.85 (s, 3H, OCH_3), 3.84 (s, 3H, OCH_3), 3.77 (s, 3H, OCH_3), 3.66 (s, 3H, OCH_3), 3.32 (d, *J* = 16.4 Hz, 1H, CH), 2.85 (d, *J* = 16.8 Hz, 1H, CH), 2.15 (s, 3H, CH_3), 1.20 (d, *J* = 6.4 Hz, 3H, CH_3); ^{13}C NMR (100 MHz, $CDCl_3$) δ: 199.5, 198.8, 170.1, 169.5, 166.8, 165.1, 148.1, 143.0, 141.4, 138.3, 136.2, 135.6, 131.0, 128.8, 128.3, 123.3, 123.1, 99.8, 73.8, 65.5, 62.5, 58.2, 52.7, 52.4, 52.1, 51.1, 36.6, 20.9, 16.4; IR(KBr) υ: 3014, 2950, 1737, 1707, 1590, 1516, 1437, 1358, 1258, 1208, 1155, 1100, 1063, 1010, 970, 899, 864, 817, 734 cm^{-1}; MS (*m/z*): HRMS (ESI) Calcd. for $C_{31}H_{31}NNaO_{10}$($[M+Na]^+$): 600.1840, found: 600.1854.

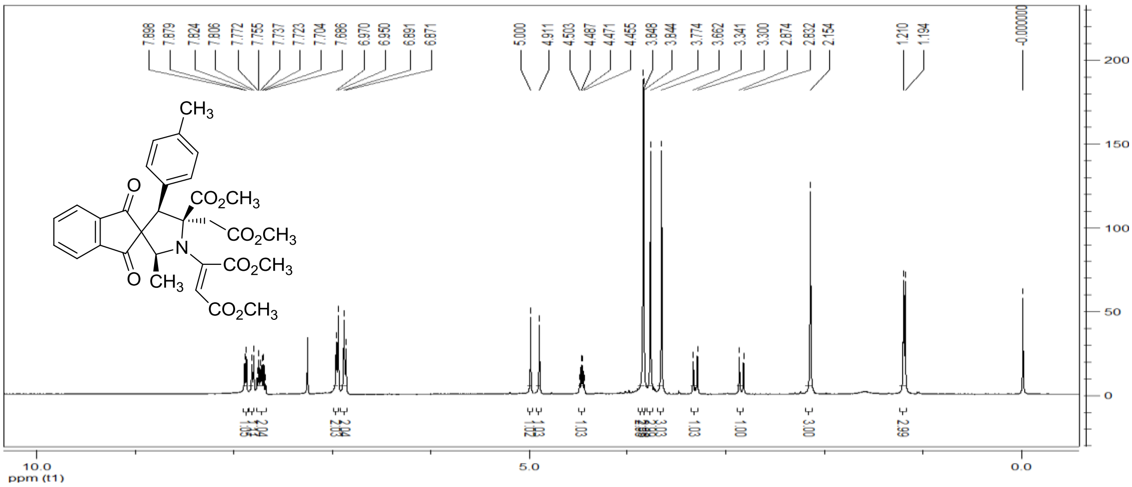

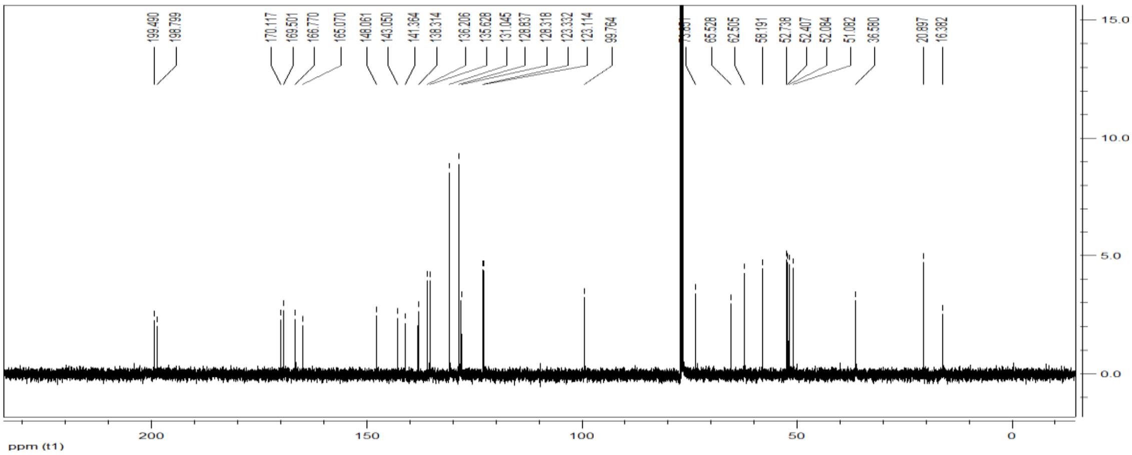

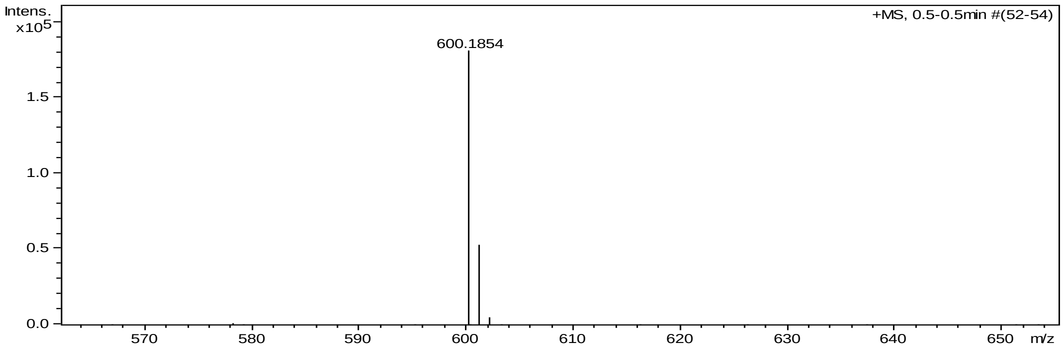

Dimethyl 2-(4'-(4-bromophenyl)-2'-(2-methoxy-2-oxoethyl)-2'-(methoxycarbonyl)-5'-methyl-1,3-dioxo-1,3-dihydrospiro[indene-2,3'-pyrrolidin]-1'-yl)maleate (3e): white solid, 76%, m.p. 210-212℃; ^{1}H NMR (600 MHz, $CDCl_3$) δ: 7.90 (d, J = 7.8 Hz, 1H, ArH), 7.84 (d, J = 7.2 Hz, 1H, ArH), 7.80 (t, J = 7.2 Hz, 1H, ArH), 7.75 (t, J = 7.2 Hz, 1H, ArH), 7.22 (d, J = 8.4 Hz, 2H, ArH), 6.99 (d, J = 8.4 Hz, 2H, ArH), 5.04 (s, 1H, CH), 4.97 (s, 1H, CH), 4.42 (q, J = 6.6 Hz, 1H, CH), 3.87 (s, 3H, OCH_3), 3.85 (s, 3H, OCH_3), 3.76 (s, 3H, OCH_3), 3.67 (s, 3H, OCH_3), 3.32 (d, J = 16.2 Hz, 1H, CH), 2.81 (d, J = 16.2 Hz, 1H, CH), 1.19 (d, J = 6.6 Hz, 3H, CH_3); ^{13}C NMR (150 MHz, $CDCl_3$) δ: 199.1, 198.9, 170.1, 169.4, 166.7, 165.0, 147.9, 142.9, 141.4, 136.5, 136.0, 133.0, 131.3, 130.7, 123.5, 123.2, 123.0, 100.5, 73.6, 65.2, 62.6, 57.2, 52.8, 52.6, 52.2, 51.2, 36.5, 16.5; IR(KBr) υ: 3094, 3012, 2949, 2846, 1739, 1706, 1587, 1491, 1438, 1357, 1267, 1216, 1152, 1104, 1006, 972, 901, 865, 805, 736 cm^{-1}; MS (m/z): HRMS (ESI) Calcd. for $C_{30}H_{28}BrNNaO_{10}$($[M+Na]^+$): 664.0789, found: 664.0801.

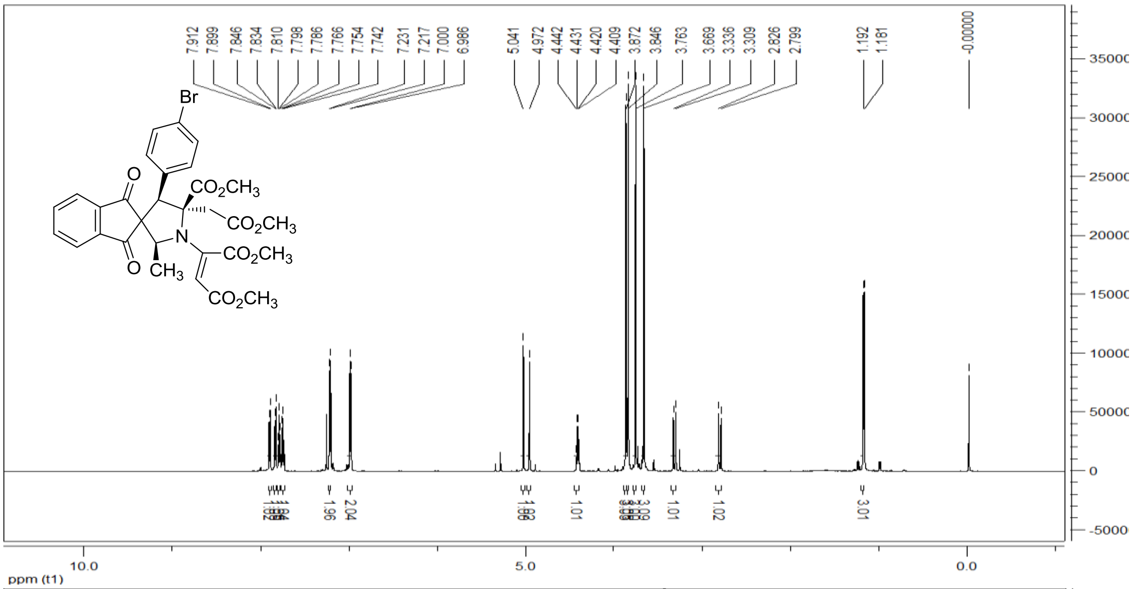

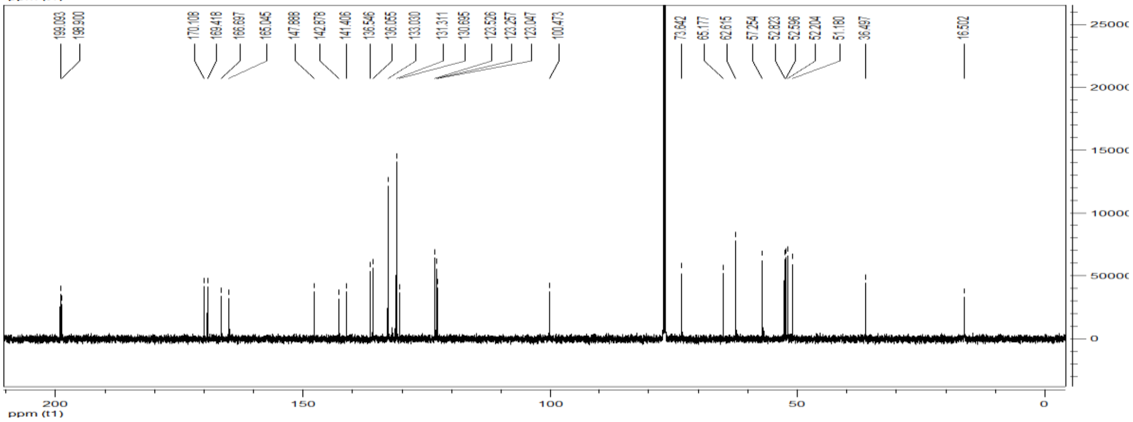

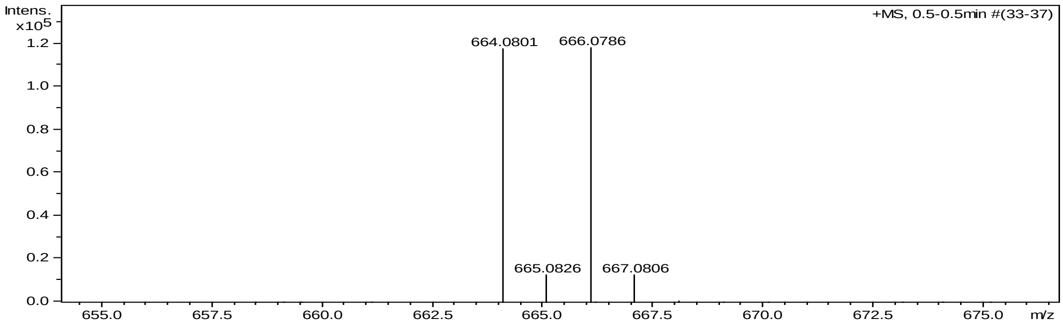

Dimethyl 2-(5'-benzyl-2'-(2-methoxy-2-oxoethyl)-2'-(methoxycarbonyl)-1,3-dioxo-4'-(p-tolyl)-1,3-dihydrospiro[indene-2,3'-pyrrolidin]-1'-yl)maleate (3f): white solid, 67%, m.p. 212-214℃; ^{1}H NMR (400 MHz, $CDCl_3$) δ: 7.62 (d, J = 7.6 Hz, 1H, ArH), 7.52-7.48 (m, 1H, ArH), 7.40-7.36 (m, 1H, ArH), 7.29 (d, J = 7.6 Hz, 1H, ArH), 6.88 (d, J = 8.4 Hz, 2H, ArH), 6.80 (d, J = 8.0 Hz, 2H, ArH), 6.75-6.73 (m, 2H, ArH), 6.72-6.68 (m, 2H, ArH), 6.67-6.63 (m, 1H, ArH), 5.22 (s, 1H, CH), 4.92-4.88 (m, 1H, CH), 4.78 (s, 1H, CH), 3.96 (s, 3H, OCH_3), 3.92 (s, 3H, OCH_3), 3.78 (s, 3H, OCH_3), 3.69 (s, 3H, OCH_3), 3.50-3.45 (m, 1H, CH), 3.27 (d, J = 16.4 Hz, 1H, CH), 3.22-3.15 (m, 1H, CH), 2.80 (d, J = 16.8 Hz, 1H, CH), 2.10 (s, 3H, CH_3); ^{13}C NMR (100 MHz, $CDCl_3$) δ: 199.8, 199.4, 170.3, 169.3, 166.8, 165.3, 148.0, 143.1, 140.1, 138.3, 135.2, 135.1, 134.7, 131.2, 129.6, 128.6, 127.8, 127.5, 126.5, 122.6, 122.1, 100.6, 73.8, 67.7, 63.4, 59.6, 52.9, 52.5, 52.1, 51.2, 36.7, 36.0, 20.8; IR(KBr) υ: 3026, 2952, 1741, 1710, 1592, 1432, 1358, 1230, 1159, 1110, 1069, 1039, 990, 876, 807, 745, 701 cm^{-1}; MS (m/z): HRMS (ESI) Calcd. for $C_{37}H_{35}NNaO_{10}$($[M+Na]^+$): 676.2153, found: 676.2160.

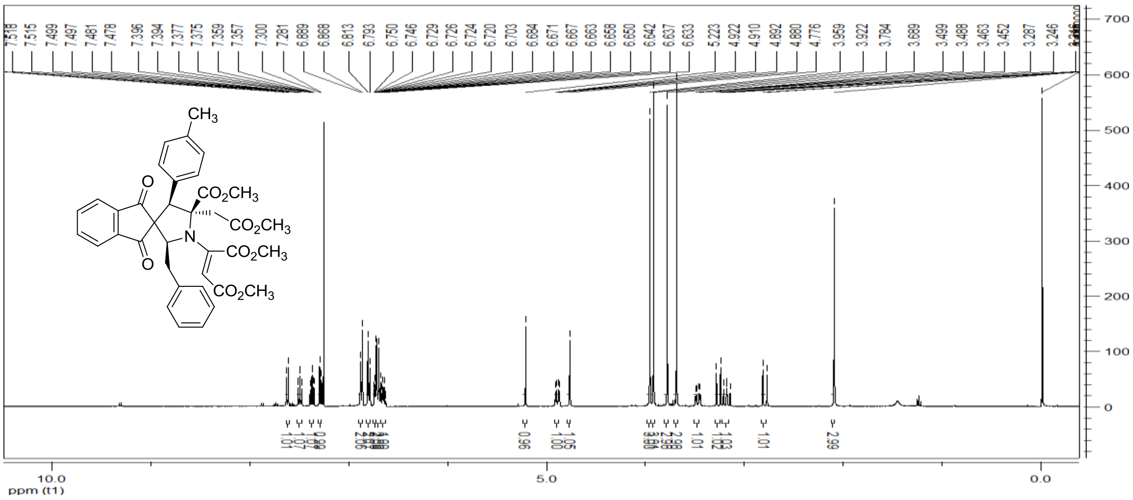

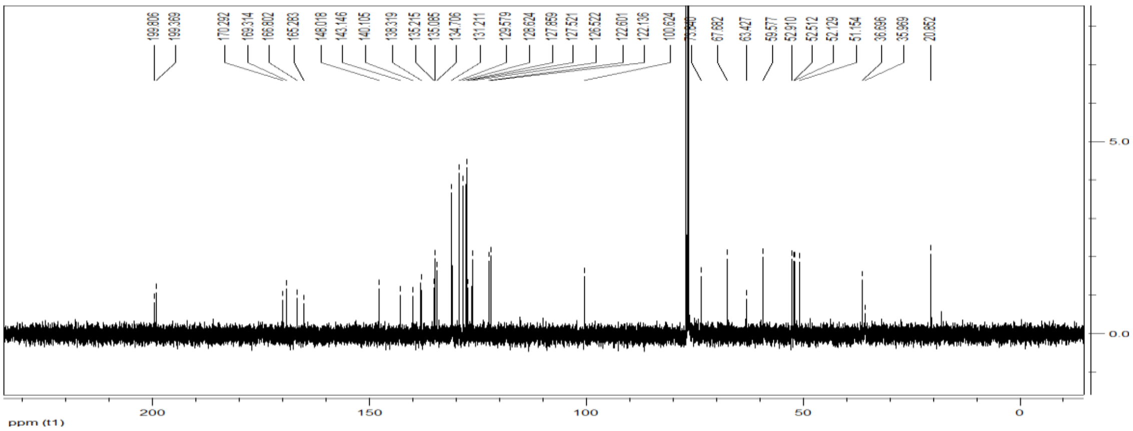

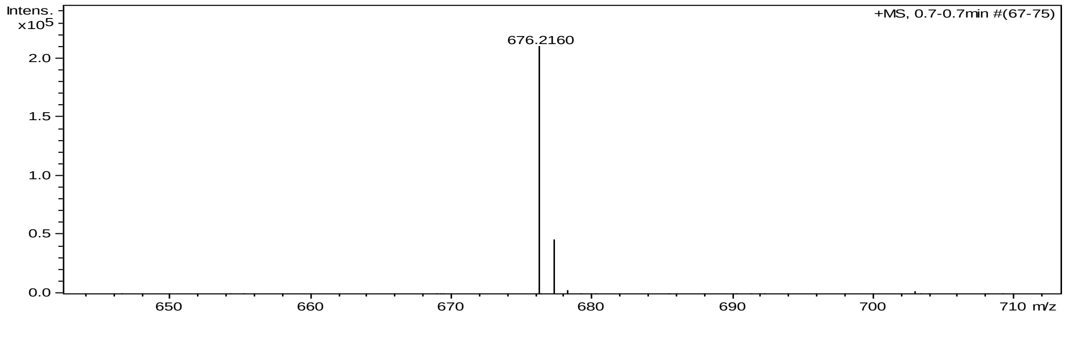

Dimethyl 2-(5'-benzyl-4'-(4-chlorophenyl)-2'-(2-methoxy-2-oxoethyl)-2'-(methoxycarbonyl)-1,3-dioxo-1,3-dihydrospiro[indene-2,3'-pyrrolidin]-1'-yl)maleate (3g): white solid, 74%, m.p. 182-184℃; ^{1}H NMR (400 MHz, $CDCl_3$) δ: 7.63 (d, J = 7.6 Hz, 1H, ArH), 7.55-7.51 (m, 1H, ArH), 7.43-7.40 (m, 1H, ArH), 7.30 (d, J = 7.6 Hz, 1H, ArH), 6.98 (brs, 4H, ArH), 6.72 (brs, 3H, ArH), 6.69-6.64 (m, 2H, ArH), 5.25 (s, 1H, CH), 4.88-4.84 (m, 2H, CH_2), 3.98 (s, 3H, OCH_3), 3.93 (s, 3H, OCH_3), 3.78 (s, 3H, OCH_3), 3.70 (s, 3H, OCH_3), 3.49-3.44 (m, 1H, CH), 3.27 (d, J = 16.4 Hz, 1H, CH), 3.18-3.11 (m, 1H, CH), 2.75 (d, J = 16.4 Hz, 1H, CH); ^{13}C NMR (100 MHz, $CDCl_3$) δ: 199.8, 199.0, 170.2, 169.2, 166.7, 165.2, 147.8, 143.0, 140.1, 135.3, 135.0, 135.0, 134.7, 132.8, 129.5, 129.3, 128.1, 127.9, 126.6, 122.7, 122.2, 101.2, 73.6, 67.7, 63.2, 58.6, 53.0, 52.7, 52.2, 51.2, 36.5; IR(KBr) υ: 3064, 3027, 2996, 2950, 1741, 1709, 1588, 1493, 1440, 1343, 1270, 1219, 1162, 1104, 1029, 883, 827, 747, 706 cm^{-1}; MS (m/z): HRMS (ESI) Calcd. for $C_{36}H_{32}ClNNaO_{10}$ ($[M+Na]^+$): 696.1607, found: 696.1609.

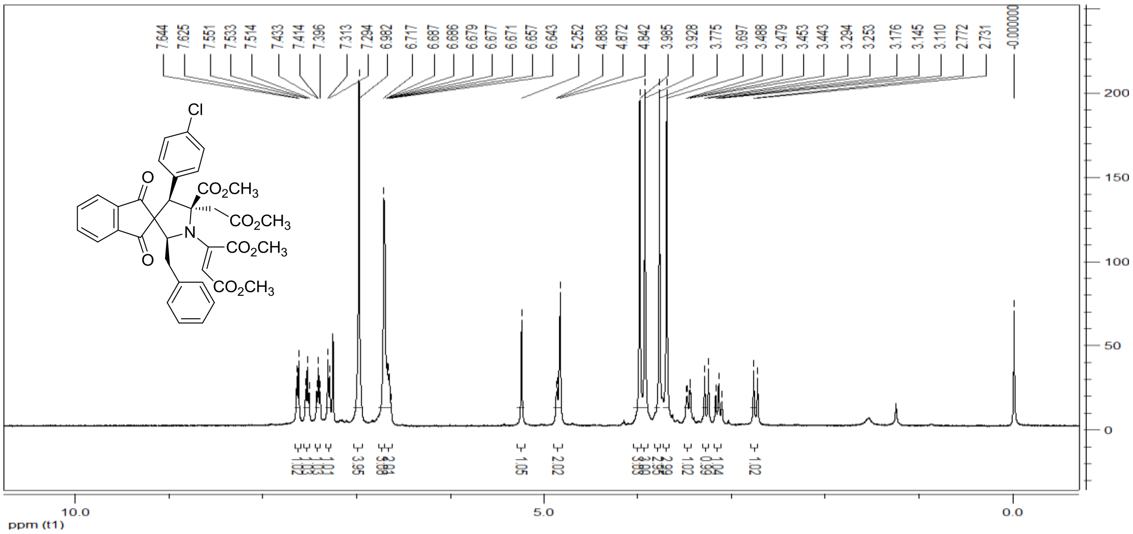

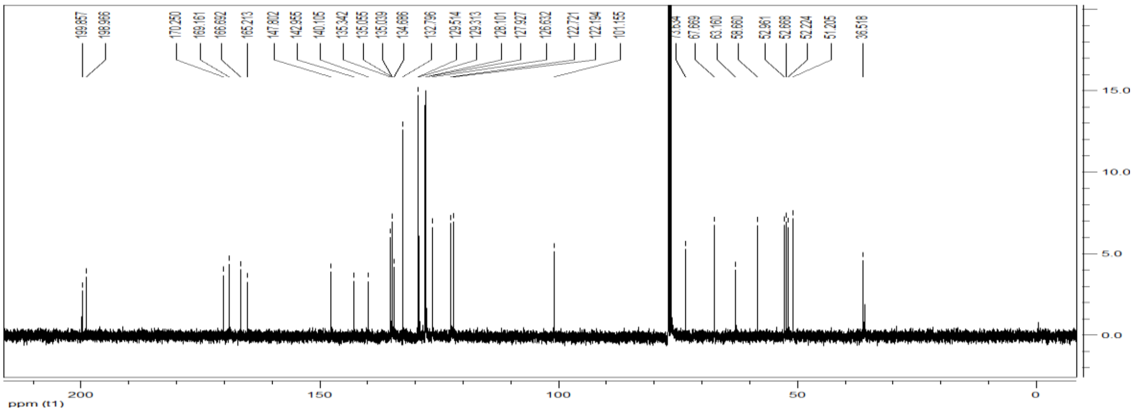

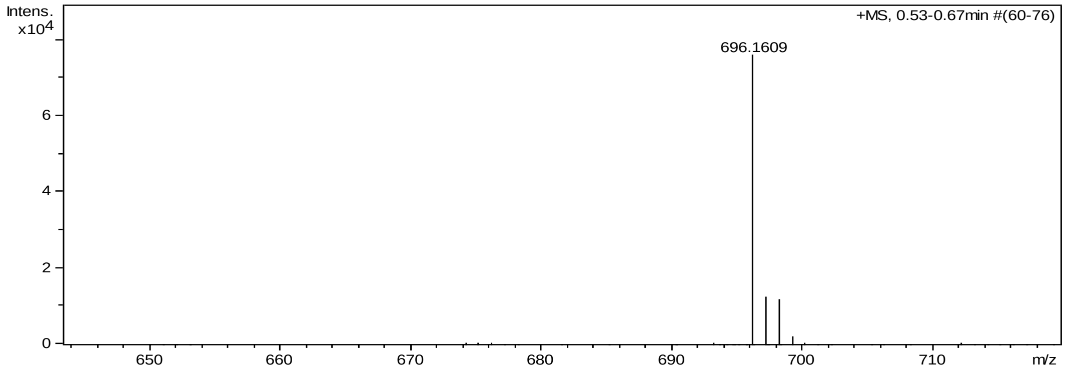

Dimethyl 2-(5'-benzyl-4'-(4-bromophenyl)-2'-(2-methoxy-2-oxoethyl)-2'-(methoxycarbonyl)-1,3-dioxo-1,3-dihydrospiro[indene-2,3'-pyrrolidin]-1'-yl)maleate (3h): white solid, 60%, m.p. 152-154℃; ^{1}H NMR (400 MHz, $CDCl_3$) δ: 7.64 (d, J = 7.6 Hz, 1H, ArH), 7.56-7.52 (m, 1H, ArH), 7.44-7.40 (m, 1H, ArH), 7.31 (d, J = 7.6 Hz, 1H, ArH), 7.16-7.13 (m, 2H, ArH), 6.93-6.89 (m, 2H, ArH), 6.72 (brs, 1H, ArH), 6.71-6.69 (m, 2H, ArH), 6.68-6.66 (m, 1H, ArH), 5.25 (s, 1H, CH), 4.88-4.85 (m, 1H, CH), 4.84 (s, 1H, CH), 3.99 (s, 3H, OCH_3), 3.93 (s, 3H, OCH_3), 3.78 (s, 3H, OCH_3), 3.70 (s, 3H, OCH_3), 3.49-3.44 (m, 1H, CH), 3.27 (d, J = 16.4 Hz, 1H, CH), 3.16-3.10 (m, 1H, CH), 2.75 (d, J = 16.4 Hz, 1H, CH); ^{13}C NMR (100 MHz, $CDCl_3$) δ: 199.8, 199.0, 170.2, 169.2, 166.7, 165.2, 147.8, 143.0, 140.1, 135.4, 135.1, 135.0, 133.1, 131.1, 129.8, 129.5, 127.9, 126.6, 123.0, 122.7, 122.2, 101.2, 73.6, 67.7, 63.1, 58.7, 53.0, 52.7, 52.2, 51.2, 36.5, 36.2; IR(KBr) υ: 3022, 2955, 2849, 1737, 1714, 1584, 1493, 1437, 1355, 1235, 1191, 1154, 1111, 1073, 1005, 967, 937, 862, 810, 747, 708 cm^{-1}; MS (m/z): HRMS (ESI) Calcd. for $C_{36}H_{32}BrNNaO_{10}$($[M+Na]^{+}$): 740.1102, found: 740.1102.

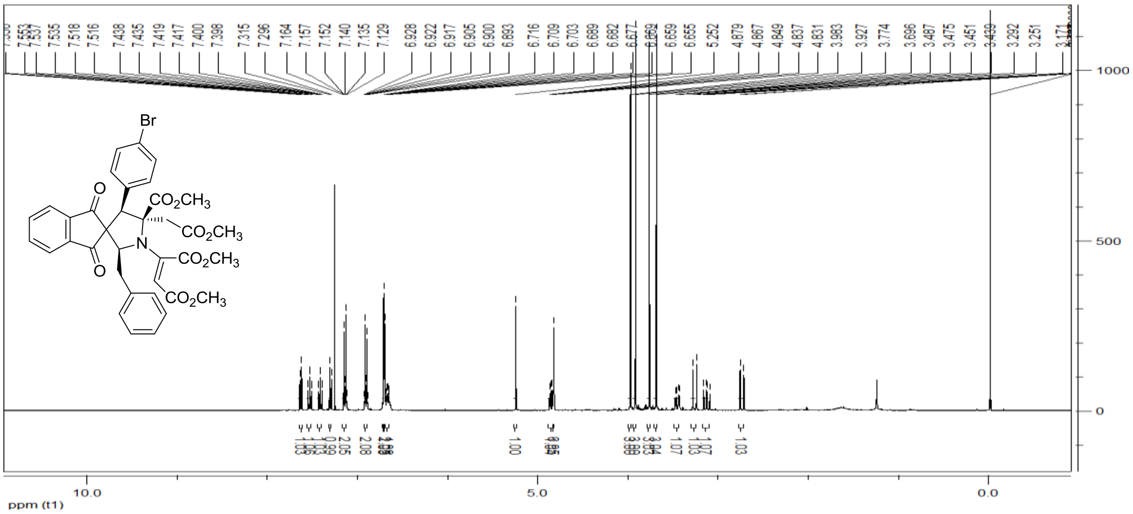

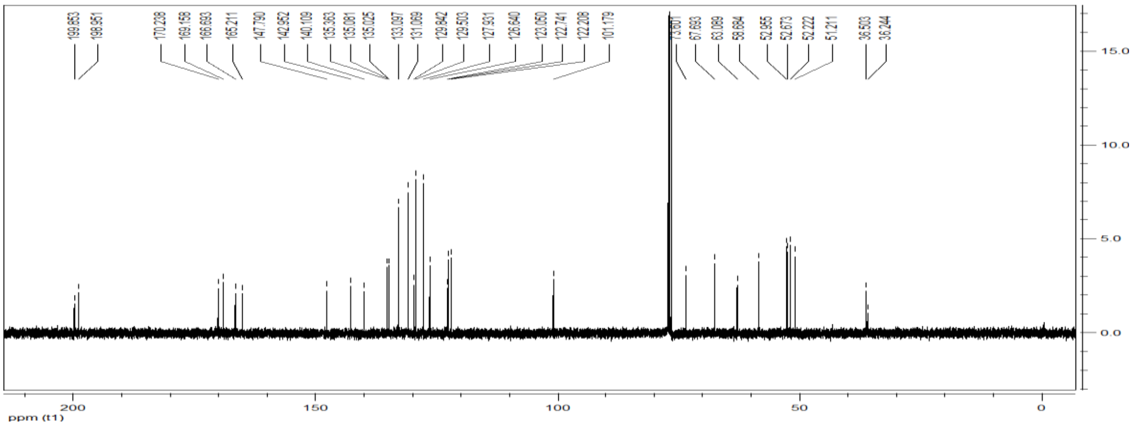

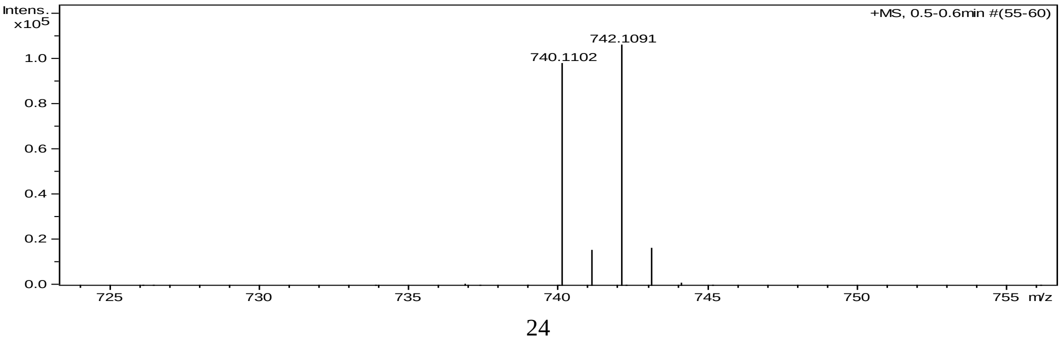

Methyl 5'-benzyl-4'-(4-bromophenyl)-2'-(2-methoxy-2-oxoethyl)-1,3-dioxo-1,3-dihydrospiro [indene-2,3'-pyrrolidine]-2'-carboxylate (4h): white solid, 19%, m.p. 146-148℃; ^{1}H NMR (400 MHz, $CDCl_3$) δ: 7.96 (d, *J* = 7.6 Hz, 1H, ArH), 7.76-7.73 (m, 1H, ArH), 7.59 (t, *J* = 7.2 Hz, 1H, ArH), 7.40 (d, *J* = 7.6 Hz, 1H, ArH), 7.23 (d, *J* = 8.4 Hz, 2H, ArH), 6.96-6.94 (m, 1H, ArH), 6.93-6.92 (m, 2H, ArH), 6.91-6.87 (m, 2H, ArH), 6.77 (d, *J* = 7.2 Hz, 2H, ArH), 4.36-4.33 (m, 1H, CH), 4.07 (s, 1H, CH), 3.79 (s, 3H, OCH_3), 3.64 (s, 3H, OCH_3), 3.59 (d, *J* = 16.4 Hz, 1H, CH), 3.09-3.04 (m, 1H, CH), 2.65 (d, *J* = 16.4 Hz, 1H, CH), 2.56-2.50 (m, 1H, CH); ^{13}C NMR (100 MHz, $CDCl_3$) δ: 201.0, 198.6, 171.7, 142.6, 142.0, 136.1, 135.7, 135.5, 132.3, 132.2, 131.5, 128.9, 128.0, 126.5, 123.0, 122.6, 122.4, 110.0, 67.4, 67.1, 66.9, 60.2, 52.8, 51.7, 41.8, 37.0; IR(KBr) υ: 3026, 2950, 2852, 1740, 1700, 1582, 1495, 1413, 1352, 1265, 1211, 1119, 1079, 1042, 1007, 908, 830, 781, 746, 703 cm^{-1}; MS (*m/z*): HRMS (ESI) Calcd. for $C_{30}H_{27}BrNO_6$($[M+H]^+$): 576.1016, found: 576.1024.

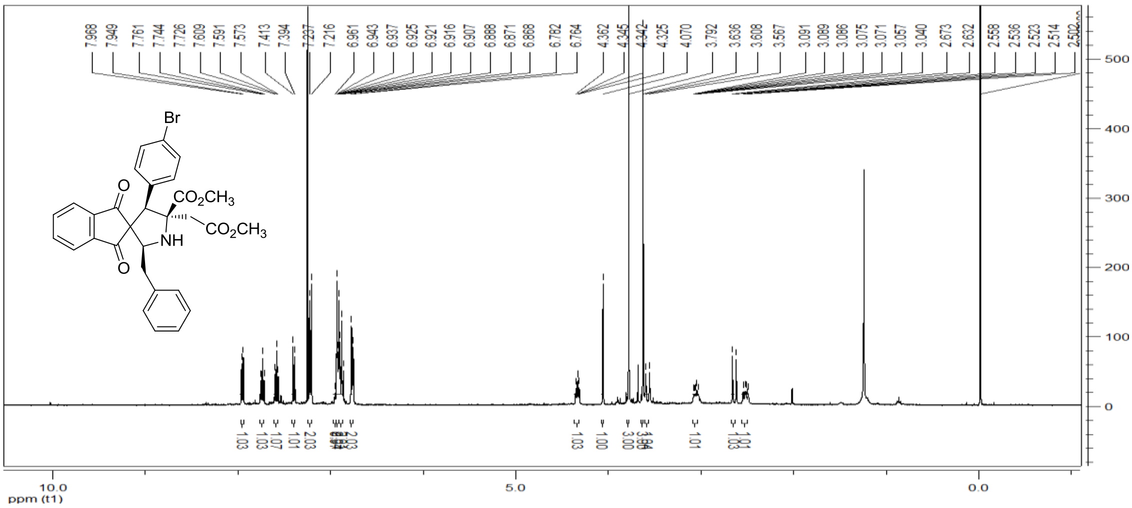

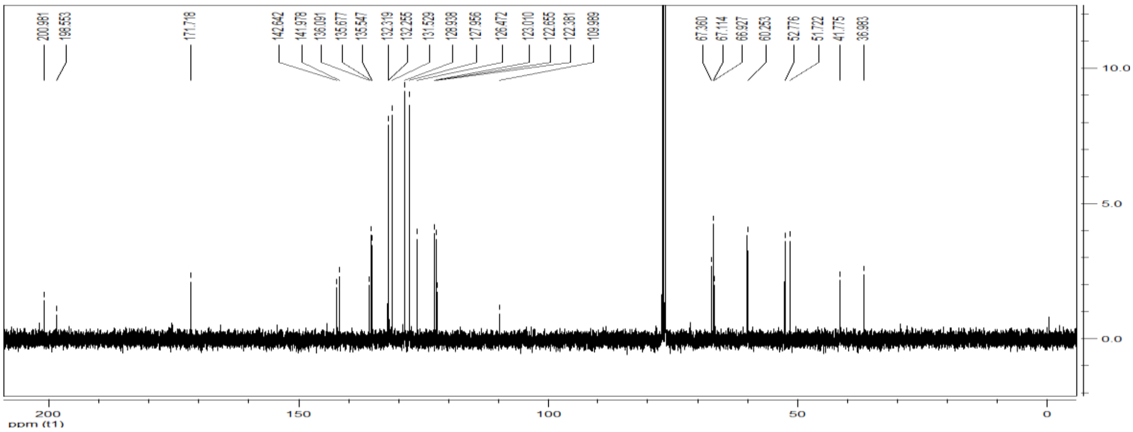

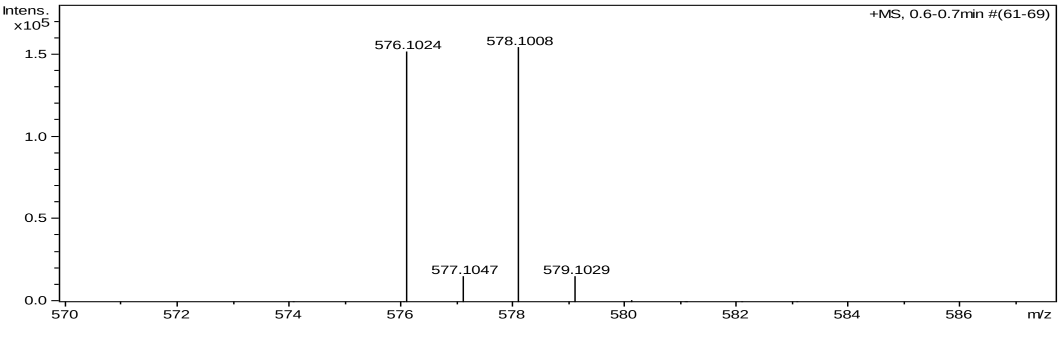

Diethyl 2-(2'-benzyl-4'-(4-bromophenyl)-5'-(2-ethoxy-2-oxoethyl)-5'-(ethoxycarbonyl)-1,3-dioxo-1,3-dihydrospiro[indene-2,3'-pyrrolidin]-1'-yl)maleate (3i): white solid, 55%, m.p. 178-180℃; ^{1}H NMR (400 MHz, $CDCl_3$) δ: 7.76 (d, J = 8.0Hz, 1H, ArH), 7.63-7.59 (m, 1H, ArH), 7.48 (t, J = 7.6Hz, 1H, ArH), 7.32 (d, J = 7.6Hz, 1H, ArH), 7.13 (d, J = 8.0Hz, 2H, ArH), 6.94 (d, J = 8.0Hz, 2H, ArH), 6.76-6.73 (m, 3H, ArH), 6.70-6.67 (m, 2H, ArH), 5.43 (s, 1H, CH), 4.77-4.73 (m, 2H, CH, CH), 4.43-4.35 (m, 2H, CH_2), 4.24-4.19 (m, 2H, CH_2), 4.17-4.12 (m, 2H, CH_2), 3.88-3.84 (m, 1H, CH), 3.82-3.78 (m, 2H, CH_2), 3.42-3.35 (m, 2H, CH_2), 2.95-2.88 (m, 1H, CH), 1.41 (t, J = 7.2Hz, 3H, CH_3), 1.28-1.23 (m, 6H, CH_3, CH_3), 1.14 (t, J = 7.2Hz, 3H, CH_3); ^{13}C NMR (100 MHz, $CDCl_3$) δ: 200.09, 199.39, 169.99, 168.69, 166.3, 164.8, 147.9, 142.9, 140.0, 135.3, 135.2, 135.0, 133.3, 132.1, 131.5, 130.9, 123.0, 129.5, 127.9, 126.6, 122.9, 122.7, 122.2, 100.9, 100.8, 73.6, 67.6, 62.9, 62.2, 62.1, 61.3, 59.8, 58.5, 58.4, 36.4, 14.4, 14.0, 13.9, 13.8; IR(KBr) υ: 2985, 1739, 1697, 1578, 1486, 1459, 1368, 1256, 1203, 1155, 1013, 950, 891, 847, 745 cm^{-1}; MS (m/z): HRMS (ESI) Calcd. for $C_{40}H_{40}BrNNaO_{10}$ ($[M+H]^+$): 796.1733, found: 796.1729.

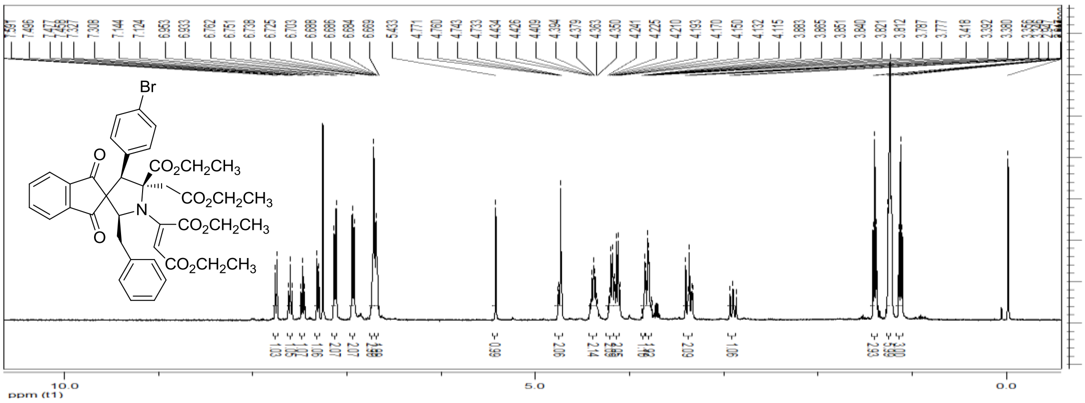

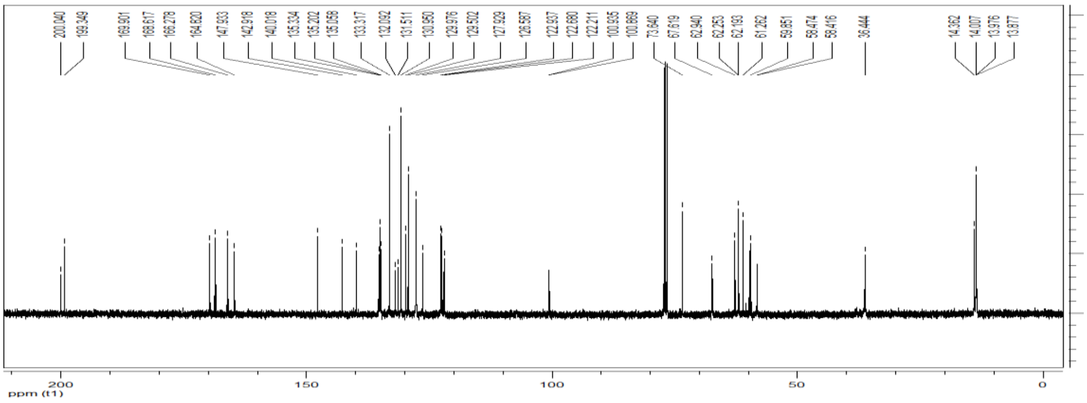

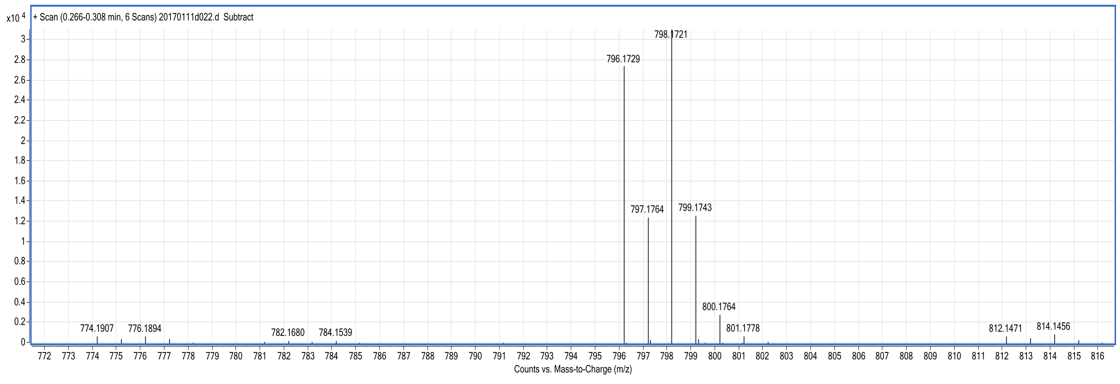

Ethyl 5'-benzyl-4'-(4-bromophenyl)-2'-(2-ethoxy-2-oxoethyl)-1,3-dioxo-1,3-dihydrospiro-[indene-2,3'-pyrrolidine]-2'-carboxylate (4i): white solid, 16%, m.p. 168-170℃; ^{1}H NMR (400 MHz, $CDCl_3$) δ: 7.96 (d, *J* = 8.0 Hz, 1H, ArH), 7.76-7.72 (m, 1H, ArH), 7.62-7.58 (m, 1H, ArH), 7.44 (d, *J* = 7.6 Hz, 1H, ArH), 7.23 (d, *J* = 8.4 Hz, 2H, ArH), 6.98-6.94 (m, 3H, ArH), 6.92-6.88 (m, 2H, ArH), 6.80 (d, *J* = 6.8 Hz, 2H, ArH), 4.69-4.67 (m, 1H, NH), 4.31-4.28 (m, 1H, CH), 4.23 (q, *J* = 7.2 Hz, 2H, CH_2), 4.08 (q, *J* = 7.2 Hz, 2H, CH_2), 4.05 (s, 1H, CH), 3.47 (d, *J* = 16.4 Hz, 1H, CH), 2.94-2.89 (m, 1H, CH), 2.58 (d, *J* = 16.4 Hz, 1H, CH), 2.48-2.42 (m, 1H, CH), 1.26-1.20 (m, 6H, CH_3, CH_3); ^{13}C NMR (100 MHz, $CDCl_3$) δ: 201.0, 198.8, 175.2, 171.3, 142.7, 142.0, 136.4, 135.6, 135.5, 132.7, 132.4, 132.2, 131.4, 131.3, 129.3, 128.9, 128.0, 126.4, 123.0, 122.6, 122.2, 67.2, 67.1, 67.0, 61.5, 60.6, 60.2, 42.2, 37.1, 14.1, 14.0; IR(KBr) υ: 3036, 2979, 2857, 1737, 1699, 1632, 1565, 1490, 1412, 1344, 1306, 1268, 1205, 1158, 1120, 1081, 1012, 945, 921, 844, 778, 744 cm^{-1}; MS (*m/z*): HRMS (ESI) Calcd. for $C_{32}H_{31}BrNO_6$($[M+H]^+$): 604.1329, found: 604.1330.

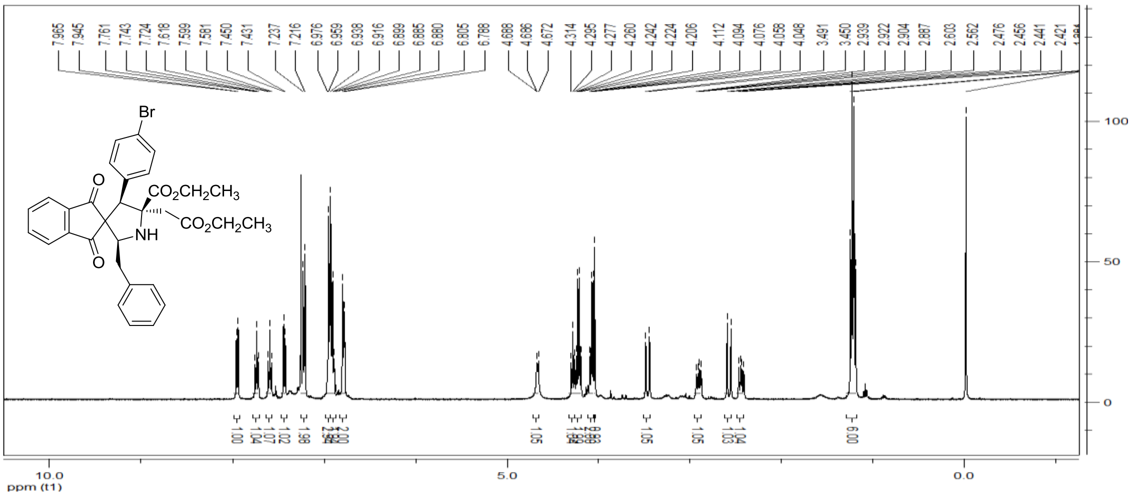

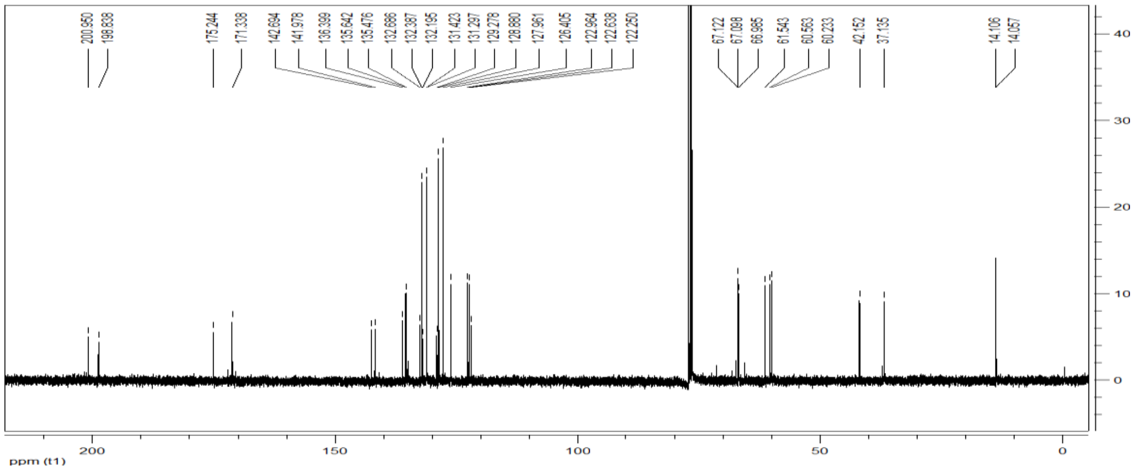

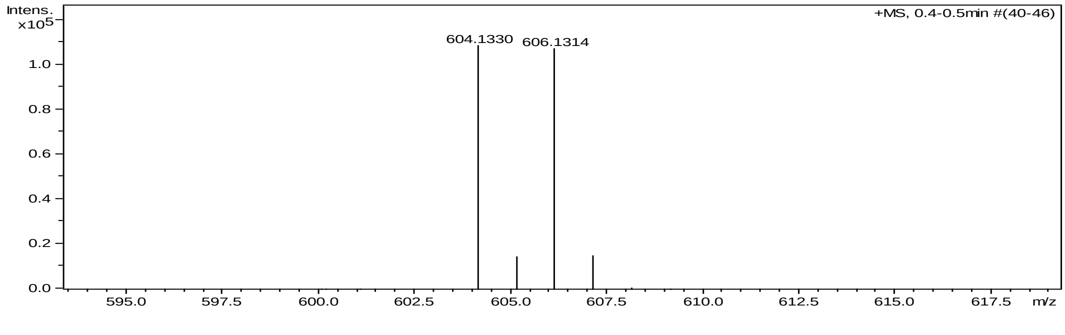

Diethyl 2-(2'-benzyl-5'-(2-ethoxy-2-oxoethyl)-5'-(ethoxycarbonyl)-1,3-dioxo-4'-(p-tolyl)-1,3-dihydrospiro[indene-2,3'-pyrrolidin]-1'-yl)maleate (3j): white solid, 72%, m.p. 186-188℃; ^{1}H NMR (400 MHz, $CDCl_3$) δ: 7.66 (d, *J* = 8.0Hz, 1H, ArH), 7.49 (t, *J* = 7.2Hz, 1H, ArH), 7.37 (t, *J* = 7.2Hz, 1H, ArH), 7.26 (d, *J* = 7.6Hz, 1H, ArH), 6.68 (brs, 1H, ArH), 6.66-6.64 (m, 3H, ArH), 6.63-6.58 (m, 1H, ArH), 5.14 (brs, 1H, CH), 4.80-4.76 (m, 1H, CH), 4.55 (brs, 1H, CH), 4.35 (t, *J* = 6.8Hz, 1H, CH), 3.83-3.78 (m, 1H, CH), 3.37-3.33 (m, 1H, CH), 3.22 (d, *J* = 14.8Hz, 1H, CH), 3.06-2.99 (m, 1H, CH), 2.02 (s, 3H, CH_3), 1.36 (t,*J* = 7.2Hz, 3H, CH_3), 1.21-1.14 (m, 7H, CH,CH_3,CH_3), 1.12 (t, *J* = 7.2Hz, 3H, CH_3); ^{13}C NMR (100 MHz, $CDCl_3$) δ: 199.7, 198.5, 169.2, 167.9, 165.4, 163.9, 147.5, 142.0, 139.3, 137.2, 134.3, 134.1, 129.2, 128.6, 128.1, 127.2, 126.8, 125.5, 121.7, 121.5, 101.3, 73.9, 66.8, 63.2, 61.2, 61.1, 60.5, 59.9, 58.8, 37.0, 35.9, 19.8, 13.3, 13.0, 12.9, 12.8; IR(KBr) υ: 2987, 2932, 2870, 1739, 1696, 1573, 1506, 1456, 1365, 1255, 1202, 1153, 1032, 952, 891, 844, 794, 746 cm^{-1}; MS (*m/z*): HRMS (ESI) Calcd. for $C_{41}H_{44}NO_{10}$ ($[M+H]^+$): 710.2965, found: 710.2979.

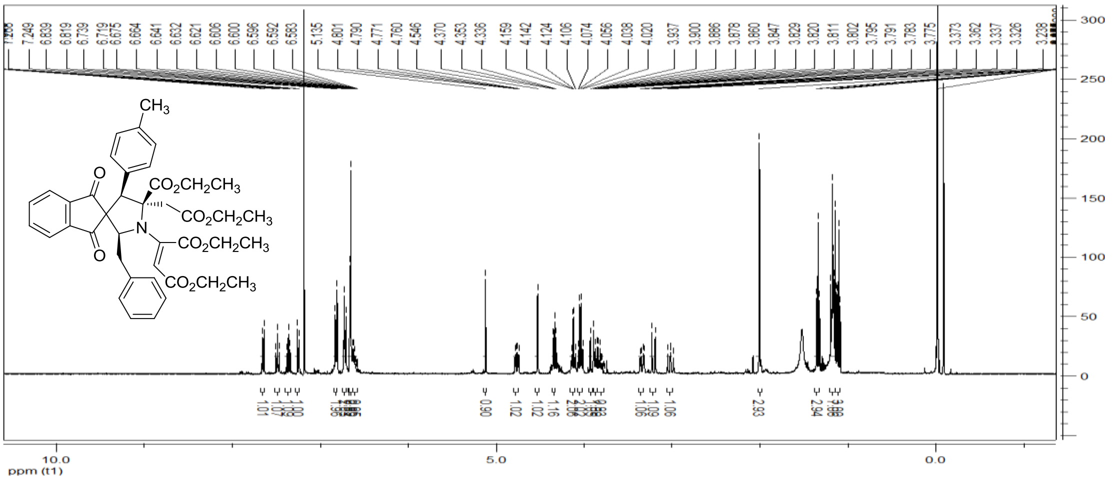

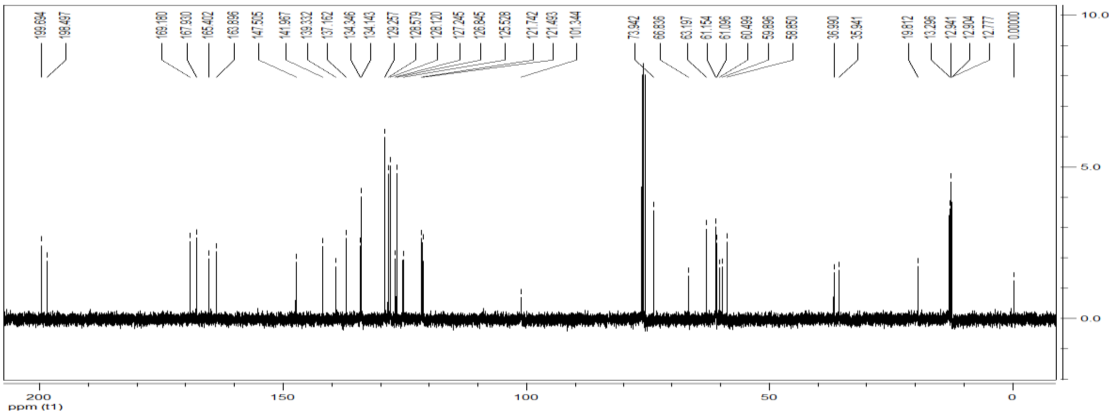

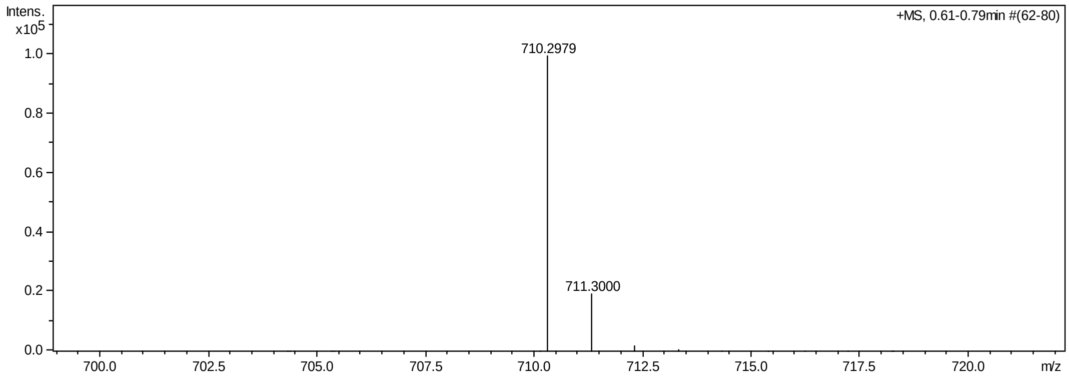

Dimethyl 2-(2'-isobutyl-5'-(2-methoxy-2-oxoethyl)-5'-(methoxycarbonyl)-1,3-dioxo-4'-(p-tolyl)-1,3-dihydrospiro[indene-2,3'-pyrrolidin]-1'-yl)maleate (3k): white solid, 74%, m.p. 136-138℃; ^{1}H NMR (400 MHz, $CDCl_3$) δ: 7.95 (d, *J* = 7.6Hz, 1H, ArH), 7.81-7.76 (m, 2H, ArH), 7.74-7.70 (m, 1H, ArH), 6.91 (d, *J* = 7.6Hz, 2H, ArH), 6.83 (d, *J* = 8.0Hz, 2H, ArH), 5.08 (s, 1H, CH), 4.58 (s, 1H, CH), 4.50-4.46 (m, 1H, CH), 3.89 (s, 3H, OCH_3), 3.84 (d, *J* = 15.2Hz, 1H, CH), 3.71 (s, 3H, OCH_3), 3.65 (s, 3H, OCH_3), 3.47 (s, 3H, OCH_3), 3.31 (d, *J* = 15.2Hz, 1H, CH), 2.12 (s, 3H, CH_3), 2.07-2.00 (m, 1H, CH), 1.65-1.61 (m, 1H, CH), 0.65-0.59 (m,6H, CH_3, CH_3); ^{13}C NMR (100 MHz, $CDCl_3$) δ: 200.3, 200.0, 170.4, 169.5, 166.9, 165.1, 148.6, 142.8, 140.4, 138.3, 136.3, 136.1, 130.1, 129.2, 128.1, 123.5, 123.0, 101.2, 101.1, 74.7, 65.3, 64.5, 62.1, 52.7, 51.8, 51.3, 51.2, 39.3, 37.6, 25.6, 23.8, 20.8, 20.6; IR(KBr) υ: 3080, 2955, 2873, 1745, 1704, 1588, 1516, 1438, 1389, 1359, 1256, 1218, 1164, 1070, 1049, 1006, 966, 868, 833, 789, 749, 731 cm^{-1}; MS (*m*/*z*): HRMS (ESI) Calcd. for $C_{34}H_{37}NNaO_{10}$ ($[M+Na]^+$): 642.2315, found: 642.2307.

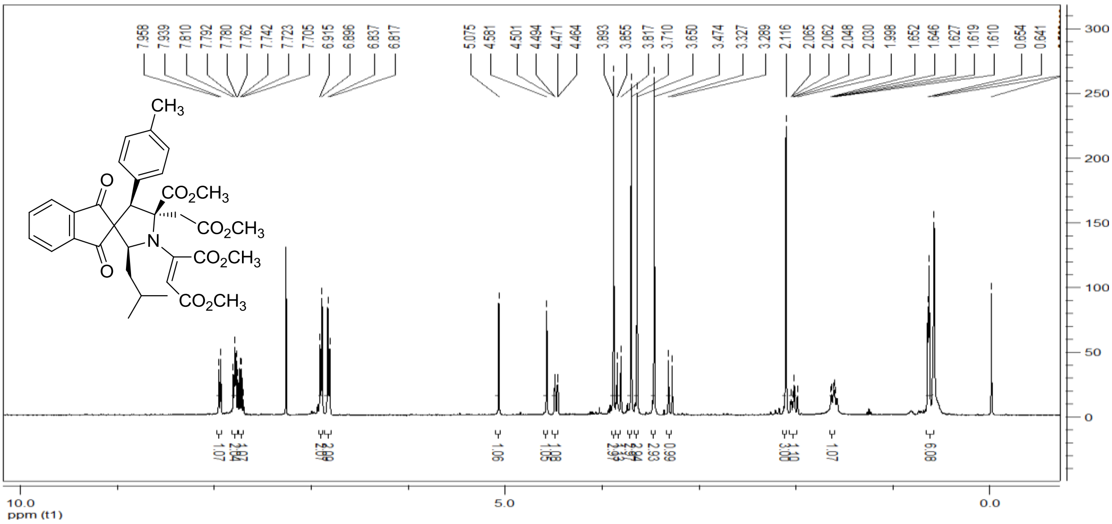

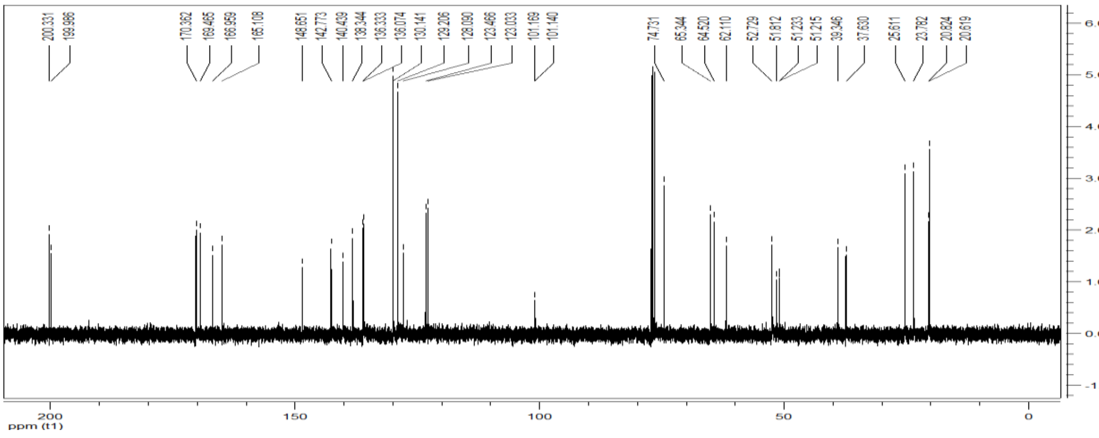

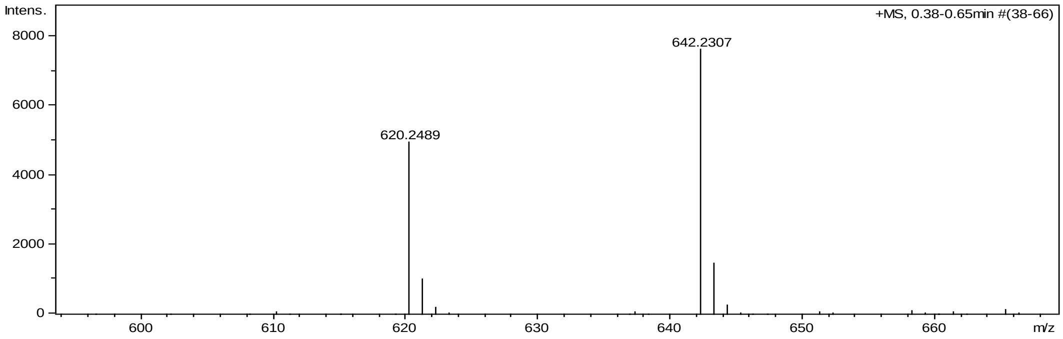

Dimethyl 2-(4'-(4-bromophenyl)-2'-isobutyl-5'-(2-methoxy-2-oxoethyl)-5'-(methoxycarbonyl)-1,3-dioxo-1,3-dihydrospiro[indene-2,3'-pyrrolidin]-1'-yl)maleate (3l): white solid, 62%, m.p. 150-152℃; ^{1}H NMR (400 MHz, $CDCl_3$) δ: 7.85 (d, *J* = 7.2Hz, 1H, ArH), 7.81-7.76 (m, 2H, ArH), 7.74-7.70 (m, 1H, ArH), 7.18 (d, *J* = 8.4Hz, 2H, ArH), 6.94 (d, *J* = 8.4Hz, 2H, ArH), 5.10 (s, 1H, CH), 4.90 (s, 1H, CH), 4.54-4.50 (m, 1H, CH), 3.94 (s, 3H, OCH_3), 3.87 (s, 3H, OCH_3), 3.74 (s, 3H, OCH_3), 3.68 (s, 3H, OCH_3), 3.22 (d, *J* = 16.4Hz, 1H, CH), 2.69 (d, *J* = 16.4Hz, 1H, CH), 2.20-2.13 (m, 1H, CH), 1.68-1.61 (m, 1H, CH), 0.62 (d, *J* = 6.4Hz, 3H, CH_3), 0.58 (d, *J* = 6.4Hz, 3H, CH_3); ^{13}C NMR (100 MHz, $CDCl_3$) δ: 199.7, 199.5, 170.3, 169.2, 166.8, 164.9, 148.0, 142.7, 140.2, 136.4, 136.0, 133.1, 131.1, 129.8, 123.5, 123.1, 122.8, 100.6, 73.2, 65.2, 63.6, 59.1, 52.8, 52.7, 52.3, 51.2, 39.6, 36.3, 25.7, 23.8, 20.6; IR(KBr) υ: 3070, 2957, 2871, 1740, 1597, 1434, 1339, 1274, 1213, 1164, 1084, 1014, 871, 829, 790, 739 cm^{-1}; MS (*m/z*): HRMS (ESI) Calcd. for $C_{33}H_{35}BrNO_{10}$ ($[M+H]^+$): 684.1444, found: 684.1443.

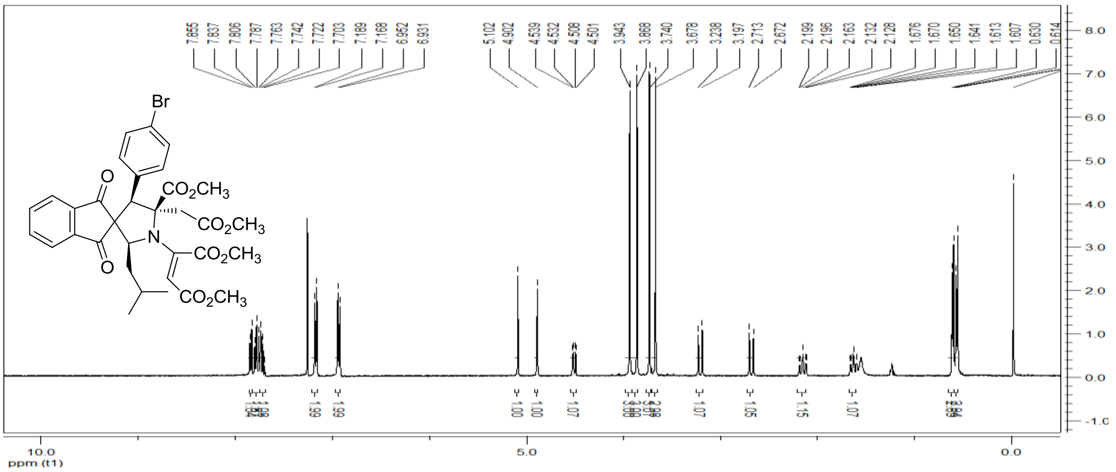

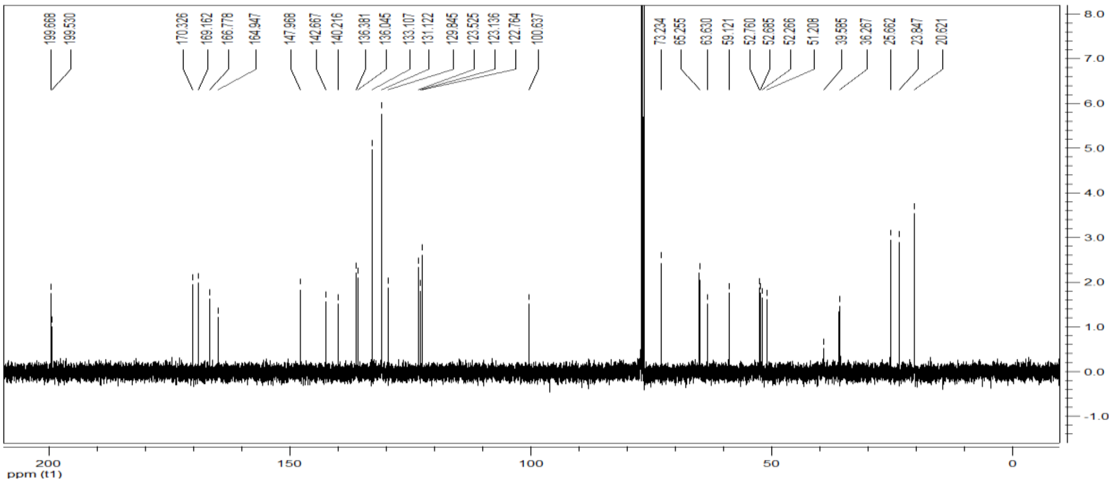

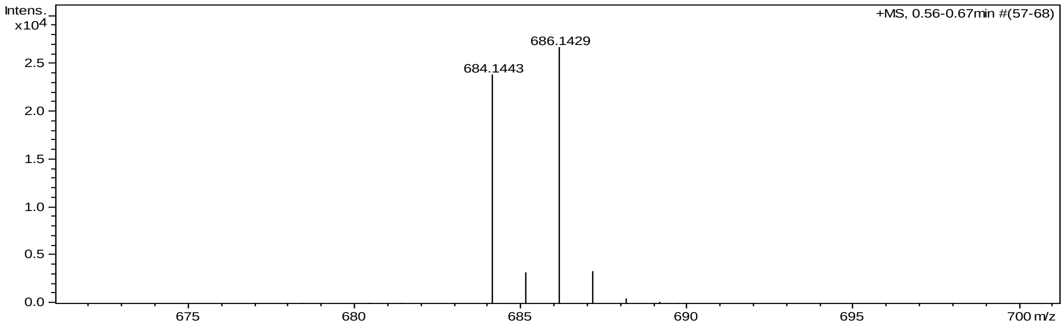

Methyl 4'-(4-bromophenyl)-2'-isobutyl-5'-(2-methoxy-2-oxoethyl)-1,3-dioxo-1,3-dihydro-spiro[indene-2,3'-pyrrolidine]-5'-carboxylate (4l): white solid, 15%, m.p. 138-140℃; ^{1}H NMR (400 MHz, $CDCl_3$) δ: 8.01 (d, J = 7.6Hz, 1H, ArH), 7.88-7.86 (m, 1H, ArH), 7.84-7.80 (m, 2H, ArH), 7.77 (d, J = 7.2Hz, 1H, ArH), 7.24 (brs, 1H, ArH), 7.01-6.96 (m, 2H, ArH), 4.08 (s, 1H, CH), 3.93-3.90 (m, 1H, CH), 3.80 (s, 3H, OCH_3), 3.68 (brs, 1H, CH), 3.63 (s, 3H, OCH_3), 3.46 (d, J = 16.4Hz,, 1H, CH), 2.61 (d, J = 16.8Hz, 1H, CH), 1.53-1.46 (m, 1H, CH), 1.30-1.25 (m, 1H, CH), 0.79-0.76 (m, 6H, CH_3, CH_3); ^{13}C NMR (100 MHz, $CDCl_3$) δ: 201.2, 199.4, 175.9, 171.9, 142.6, 142.0, 136.2, 136.0, 132.8, 132.3, 131.5, 123.2, 123.1, 122.3, 67.9, 67.0, 64.7, 59.4, 52.8, 51.7, 41.9, 38.9, 25.5, 23.3, 21.2; IR(KBr) υ: 3352, 2954, 2876, 1744, 1703, 1592, 1487, 1435, 1352, 1260, 1209, 1077, 1004, 926, 868, 803, 767, 718 cm^{-1}; MS (m/z): HRMS (ESI) Calcd. for $C_{27}H_{29}BrNO_6$ ($[M+H]^+$): 542.1178, found: 542.1199.

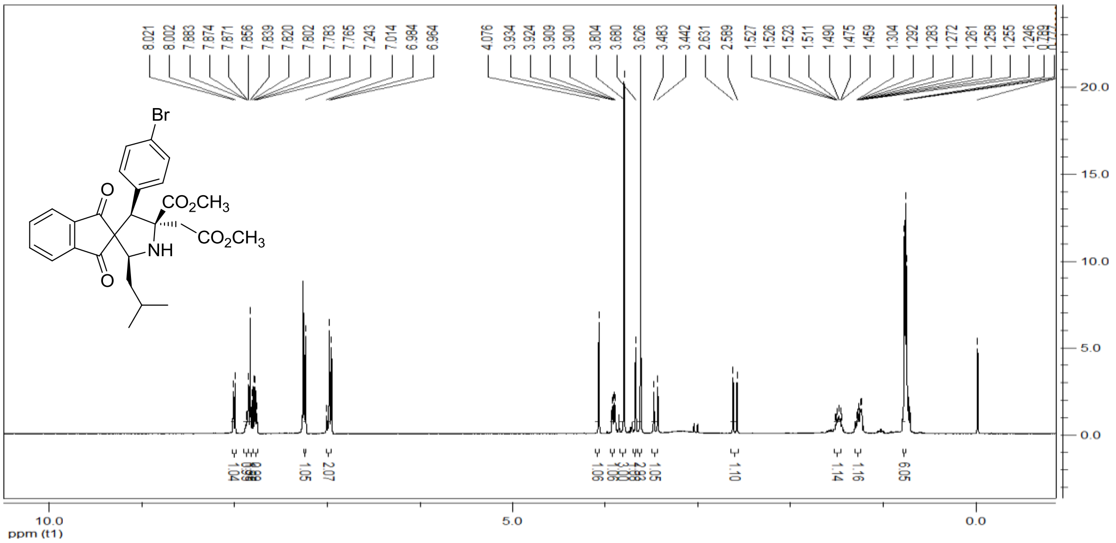

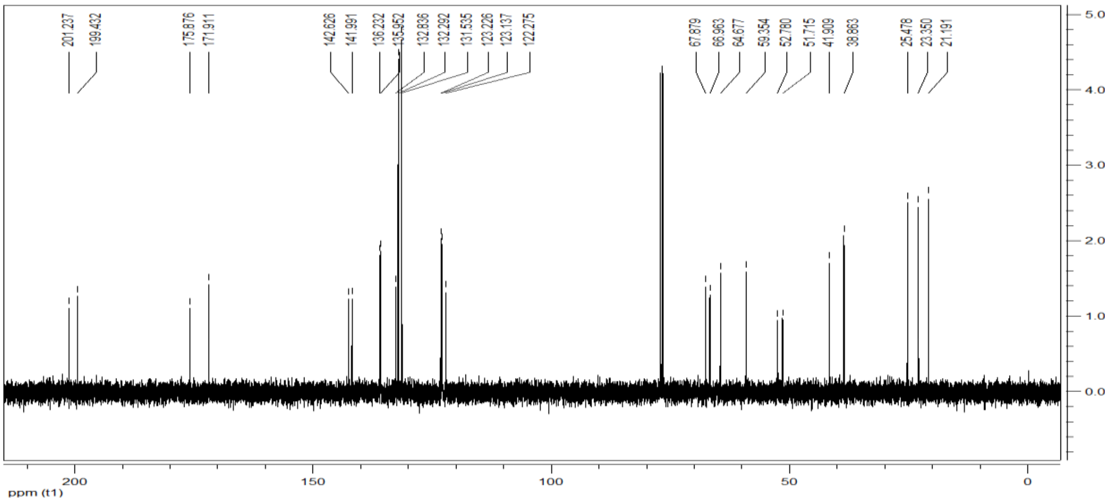

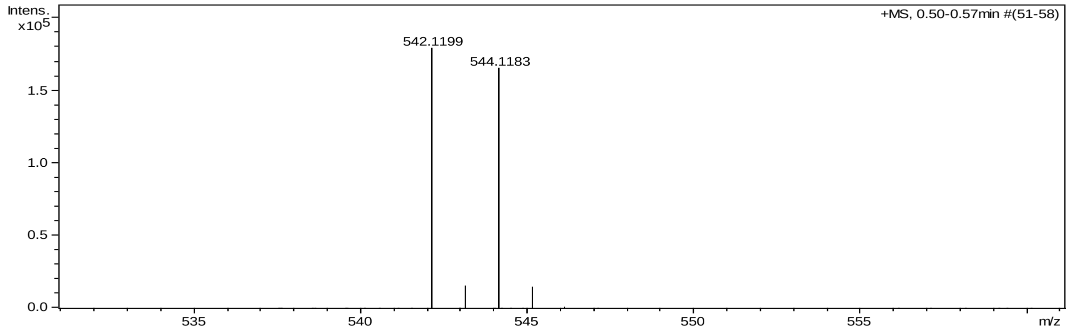

Dimethyl 2-(4'-(3-chlorophenyl)-2'-isobutyl-5'-(2-methoxy-2-oxoethyl)-5'-(methoxycarbonyl)-1,3-dioxo-1,3-dihydrospiro[indene-2,3'-pyrrolidin]-1'-yl)maleate (3m): white solid, 78%, m.p. 156-158℃; ^{1}H NMR (400 MHz, $CDCl_3$) δ: 7.86 (d, J = 7.2Hz, 1H, ArH), 7.81-7.77 (m, 1H, ArH), 7.75-7.69 (m, 2H, ArH), 7.07-7.06 (m, 2H, ArH), 7.00-6.93 (m, 2H, ArH), 5.10 (s, 1H, CH), 4.90 (s, 1H, CH), 4.57-4.53 (m, 1H, CH), 3.93 (s, 3H, OCH_3), 3.87 (s, 3H, OCH_3), 3.75 (s, 3H, OCH_3), 3.68 (s, 3H, OCH_3), 3.24 (d, J = 16.4Hz, 1H, CH), 2.72 (d, J = 16.4Hz, 1H, CH), 2.32-2.16 (m, 1H, CH), 1.69-1.62 (m, 1H, CH), 0.64 (d, J = 6.0Hz, 3H, CH_3), 0.59 (d, J = 6.4Hz, 3H, CH_3); ^{13}C NMR (100 MHz, $CDCl_3$) δ: 199.6, 199.3, 170.2, 169.2, 166.8, 165.0, 148.0, 142.7, 140.1, 136.3, 135.9, 133.7, 132.8, 131.5, 129.6, 129.1, 128.8, 123.5, 122.8, 100.8, 73.4, 65.2, 63.7, 59.3, 52.8, 52.7, 52.3, 51.2, 39.5, 36.3, 25.7, 23.9, 20.6; IR(KBr) υ: 3072, 3002, 2953, 2880, 1743, 1709, 1594, 1436, 1362, 1268, 1218, 1162, 1097, 1035,1006, 956, 913, 869, 853, 788, 746 cm^{-1}; MS (m/z): HRMS (ESI) Calcd. for $C_{33}H_{34}ClNNaO_{10}$ ($[M+Na]^+$): 662.1769, found: 662.1761.

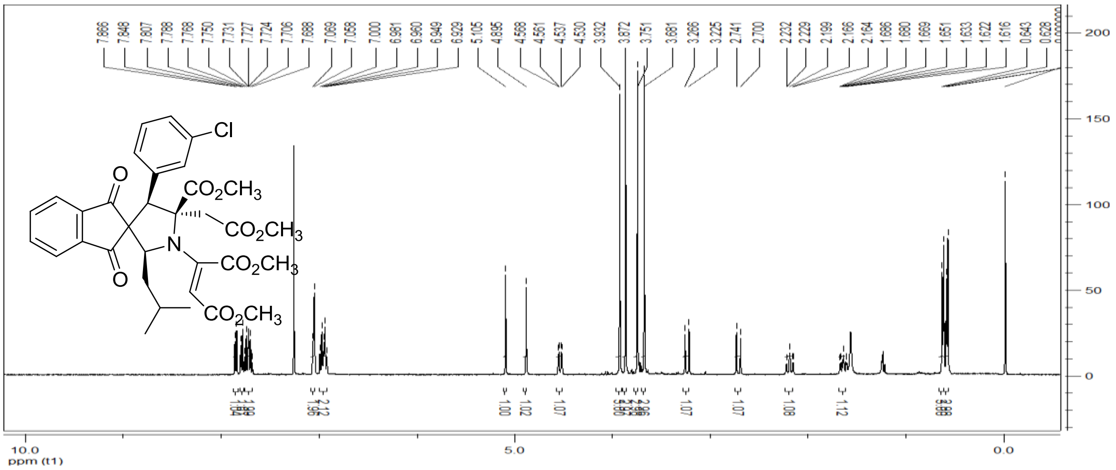

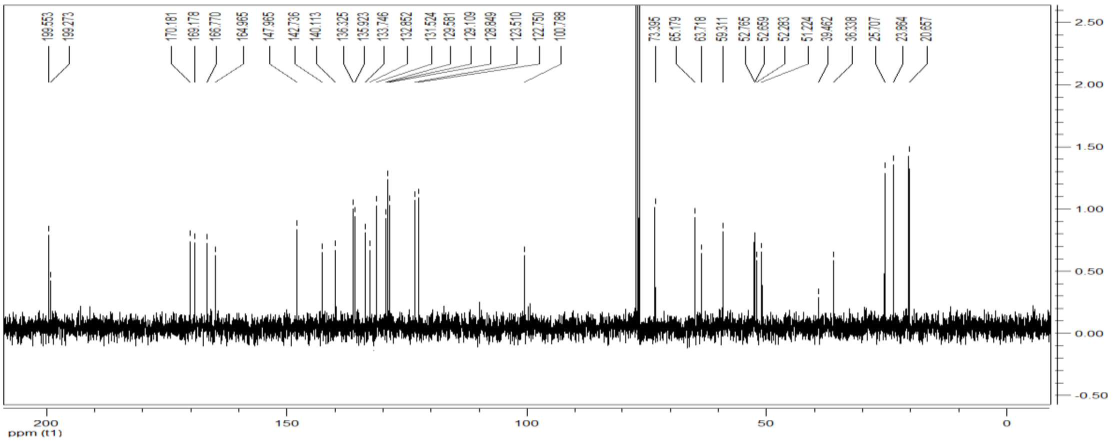

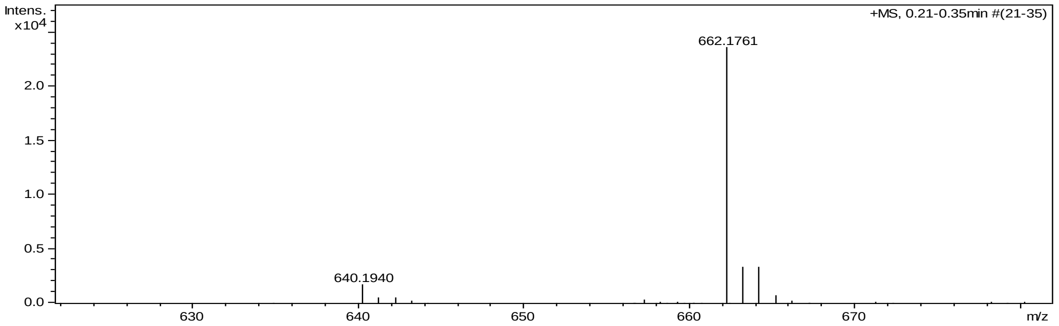

Dimethyl 2-(2'-isobutyl-5'-(2-methoxy-2-oxoethyl)-5'-(methoxycarbonyl)-4'-(4-methoxyphenyl)-1,3-dioxo-1,3-dihydrospiro[indene-2,3'-pyrrolidin]-1'-yl)maleate (3n): white solid, 58%, m.p. 134-136℃; ^{1}H NMR (400 MHz, $CDCl_3$) δ: 7.94 (d, *J* = 7.6Hz, 1H, ArH), 7.82-7.78 (m, 2H, ArH), 7.75-7.71 (m, 1H, ArH), 6.95 (d, *J* = 8.8Hz, 2H, ArH), 6.55 (d, *J* = 8.4Hz, 2H, ArH), 5.07 (s, 1H, CH), 4.57 (s, 1H, CH), 4.50-4.46 (m, 1H, CH), 3.90 (s, 3H, OCH_3), 3.83 (d, *J* = 14.8Hz, 1H, CH), 3.71 (s, 3H, OCH_3), 3.65 (s, 3H, OCH_3), 3.63 (s, 3H, OCH_3), 3.50 (s, 3H, OCH_3), 3.32 (d, *J* = 15.2Hz, 1H, CH), 2.07-2.00 (m, 1H, CH), 1.65-1.61 (m, 1H, CH), 0.66-0.64 (m, 3H, CH_3), 0.60-0.59 (m, 3H, CH_3); ^{13}C NMR (100 MHz, $CDCl_3$) δ: 200.4, 200.1, 170.4, 169.5, 167.0, 165.1, 159.4, 148.6, 142.8, 140.5, 136.3, 136.1, 131.5, 123.4, 123.1, 123.0, 113.8, 101.4, 101.3, 101.2, 74.6, 65.2, 64.7, 61.8, 61.7, 55.0, 54.9, 52.8, 52.7, 52.6, 51.9, 51.8, 51.3, 39.4, 37.6, 29.6, 25.6, 23.8, 20.6; IR(KBr) υ: 3078, 2956, 2861, 1744, 1702, 1588, 1514, 1441, 1394, 1361, 1256, 1214, 1162, 1029, 964, 876, 835, 773, 730 cm^{-1}; MS (*m/z*): HRMS (ESI) Calcd. for $C_{34}H_{37}NNaO_{11}$ ($[M+Na]^+$): 658.2264, found: 658.2279.

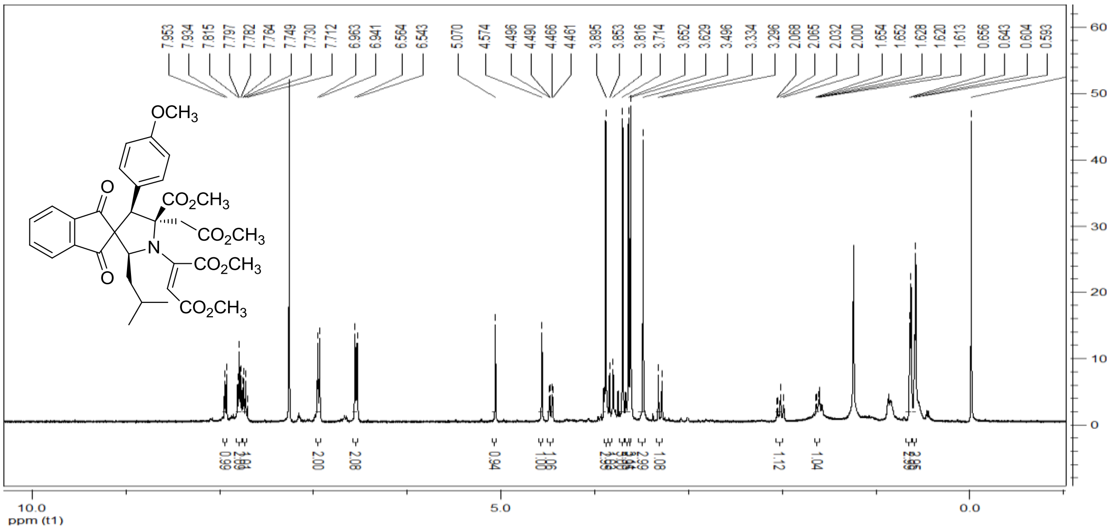

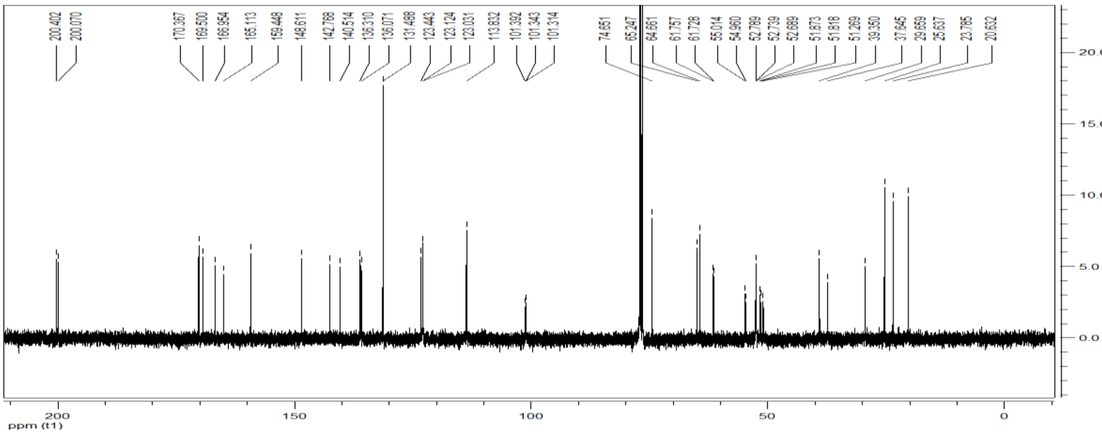

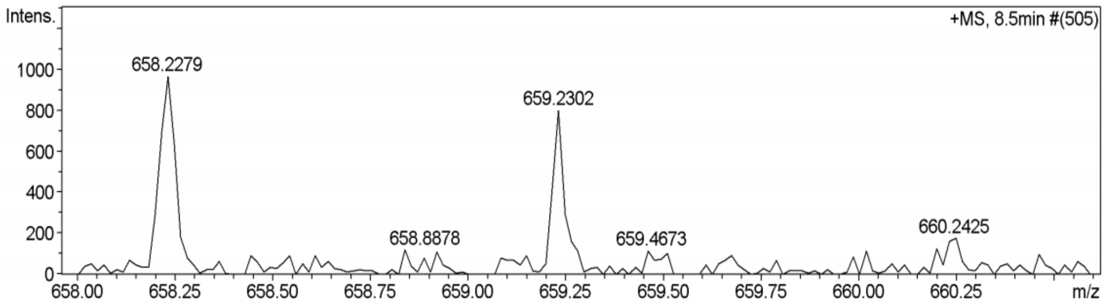

Methyl 2'-isobutyl-5'-(2-methoxy-2-oxoethyl)-4'-(4-methoxyphenyl)-1,3-dioxo-1,3-dihydro-spiro[indene-2,3'-pyrrolidine]-5'-carboxylate (4n): white solid, 12%, m.p. 132-134℃; ^{1}H NMR (400 MHz, $CDCl_3$) δ: 8.01 (d, *J* = 7.2Hz, 1H, ArH), 7.84-7.79 (m, 2H, ArH), 7.77-7.74 (m, 1H, ArH), 7.00 (d, *J* = 8.8Hz, 2H, ArH), 6.64 (d, *J* = 8.8Hz, 2H, ArH), 4.05 (s, 1H, CH), 3.96-3.92 (m, 1H, CH), 3.80 (s, 3H, OCH_3), 3.69-3.67 (m, 4H, CH,OCH_3), 3.63 (s, 3H, OCH_3), 3.48 (d, *J* = 16.8Hz,, 1H, CH), 2.63 (d, *J* = 16.8Hz, 1H, CH), 1.52-1.46 (m, 1H, CH), 1.34-1.29 (m, 1H, CH), 0.80-0.77 (m, 6H, CH_3, CH_3); ^{13}C NMR (100 MHz, $CDCl_3$) δ: 201.5, 199.9, 176.2, 172.2, 159.0, 142.8, 142.1, 136.0, 135.7, 131.8, 125.6, 123.0, 113.7, 110.0, 68.0, 67.1, 64.4, 59.7, 55.0, 52.7, 51.7, 42.0, 38.9, 25.5, 23.3, 21.2; IR(KBr) υ: 3339, 2955, 2850, 1742, 1699, 1600, 1516, 1444, 1353, 1254, 1211, 1104, 1030, 1001, 872, 833, 805, 769, 707 cm^{-1};MS (*m/z*): HRMS (ESI) Calcd. for $C_{28}H_{32}NO_7$ ([M+H]$^+$): 494.2179, found: 494.2208.

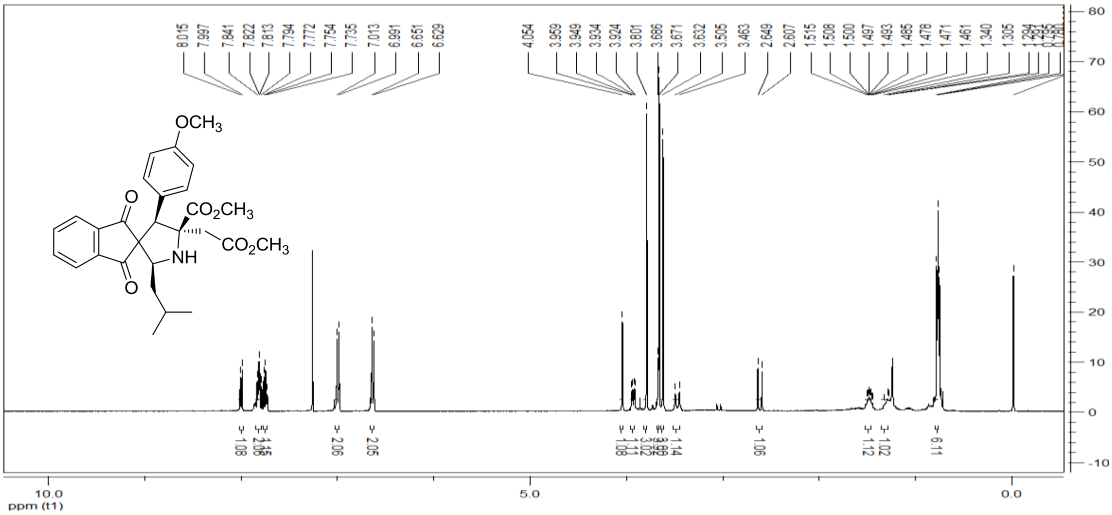

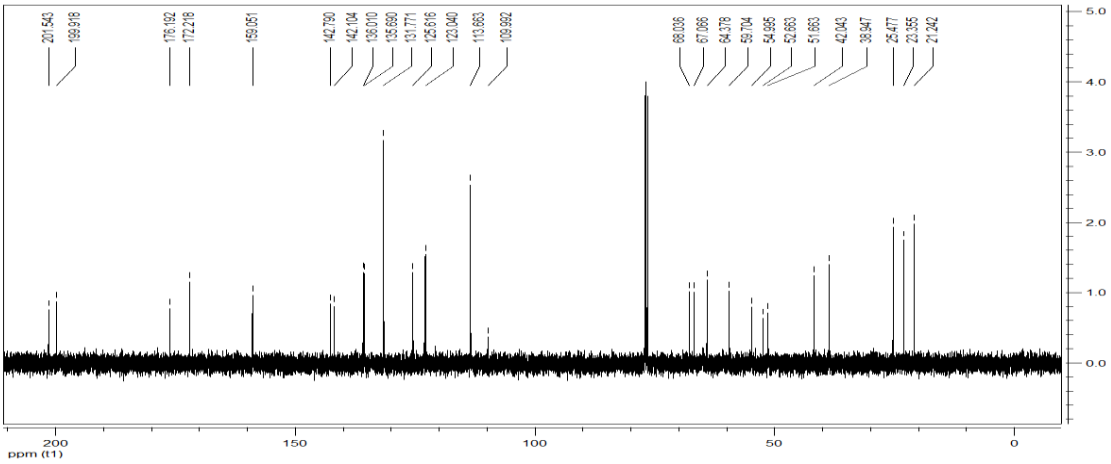

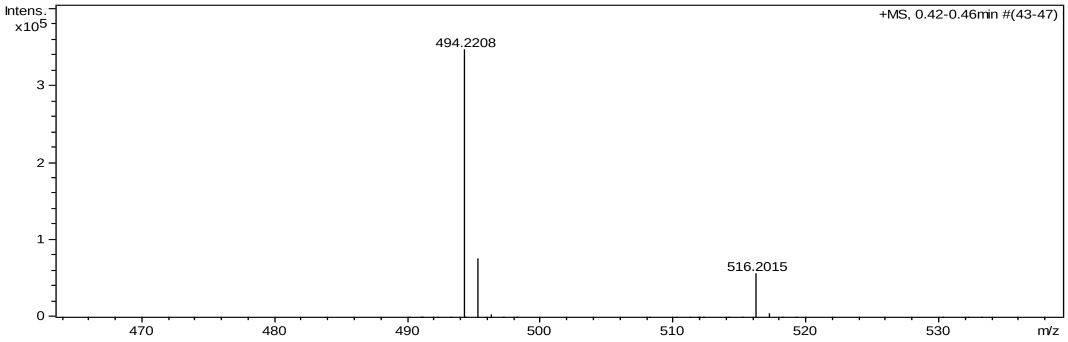

Supplement: Supplementary file 1 — Supplementary Information [file 41598_2017_12361_MOESM1_ESM.pdf]
